# Supplementary material for: Connexins evolved after early chordates lost innexin diversity
Source: eLife. 2022 Jan 19;11:e74422. doi: 10.7554/eLife.74422 (PMC8769644; doi:10.7554/eLife.74422)
Supplement: Figure 1—source data 1. [file elife-74422-fig1-data1.zip › Figure 1source data 1.docx]

**Figure 1–source data 1. A list of the identified N-glycosylation sites (NGS) in the extracellular loops of innexins in non-chordate species.**

| **Taxonomic rank** | **Species** | **Innexin**  **ID** | **Database** | **Accession**  **ID** | **Predicted NGS** | |
| --- | --- | --- | --- | --- | --- | --- |
|  |  |  |  |  | **EL1** | **EL2** |
|  |  |  |  |  |  |  |
| **Ctenophores** | *Pleurobrachia bachei* | Ct_Pba_01 | Ctenophora Genomes | 2663906 |  | N251 |
|  |  | Ct_Pba_02 | Ctenophora Genomes | 2647758 |  |  |
|  |  | Ct_Pba_03 | Ctenophora Genomes | 2665840 |  |  |
|  |  | Ct_Pba_04 | Ctenophora Genomes | 2663329 | N112 |  |
|  |  | Ct_Pba_05 | Ctenophora Genomes | 2667905 |  | N211 |
|  | *Beroe abyssicola* | Ct_Bab_01 | Ctenophora Genomes | 12132532 | N131 | N340 |
|  |  | Ct_Bab_02 | Ctenophora Genomes | 12139961 |  | N257 |
|  |  | Ct_Bab_03 | Ctenophora Genomes | 12139962 |  | N259 |
|  |  | Ct_Bab_04 | Ctenophora Genomes | 12125868 |  | N258 |
|  | *Bolinopsis infundibulum* | Ct_Bin_01 | Ctenophora Genomes | 12228714 |  | N262 |
|  |  | Ct_Bin_02 | Ctenophora Genomes | 12216757 |  |  |
|  | *Dryodora glandiformis* | Ct_Dgl_01 | Ctenophora Genomes | 306622 |  | N228 |
|  |  | Ct_Dgl_02 | Ctenophora Genomes | 306068 |  | N250 |
|  | *Euplokamis dunlapae* | Ct_Edu_01 | Ctenophora Genomes | 10651747 |  |  |
|  |  | Ct_Edu_02 | Ctenophora Genomes | 10651741 |  | N228 |
|  |  | Ct_Edu_03 | Ctenophora Genomes | 10635300 | N53 | N262 |
|  | *Vallicula multiformis* | Ct_Vmu_01 | Ctenophora Genomes | 445178 |  | N249 |
|  |  | Ct_Vmu_02 | Ctenophora Genomes | 470312 |  | N259 |
|  |  | Ct_Vmu_03 | Ctenophora Genomes | 445166 |  | N251 |
|  | *Pleurobrachia bachei pileus* | Ct_Pbp_01 | Ctenophora Genomes | 12713923 | N112 |  |
|  |  | Ct_Pbp_02 | Ctenophora Genomes | 12681783 |  | N228 |
|  |  | Ct_Pbp_03 | Ctenophora Genomes | 12713172 |  | N220 |
|  |  | Ct_Pbp_04 | Ctenophora Genomes | 12728891 |  | N269 |
|  | *Hormiphora californensis* | Ct_Hca_01 | NCBI TSA | GGLO01022876.1 |  | 237 |
|  |  | Ct_Hca_02 | NCBI TSA | GGLO01032427.1 | 79 |  |
|  |  | Ct_Hca_03 | NCBI TSA | GGLO01040902.1 |  |  |
|  |  | Ct_Hca_04 | NCBI TSA | GGLO01044036.1 |  | 228 |
|  |  | Ct_Hca_05 | NCBI TSA | GGLO01055378.1 | 112 | 269 |
|  |  | Ct_Hca_06 | NCBI TSA | GGLO01061621.1 |  | 249 |
|  |  | Ct_Hca_07 | NCBI TSA | GGLO01065756.1 |  | 250 |
|  |  | Ct_Hca_08 | NCBI TSA | GGLO01068976.1 |  | 255 |
|  |  | Ct_Hca_09 | NCBI TSA | GGLO01006002.1 | 45 | 254 |
|  | *Mnemiopsis leidyi* | Ct_Mle_01 | NCBI TSA | GFAT01025049.1 |  | 227 |
|  |  | Ct_Mle_02 | NCBI TSA | GFAT01117950.1 |  |  |
|  |  | Ct_Mle_03 | NCBI TSA | GFAT01120318.1 |  | 255 |
|  |  | Ct_Mle_04 | NCBI TSA | GFAT01094561.1 | 45 | 254 |
|  |  | Ct_Mle_05 | NCBI TSA | GFAT01105573.1 | 113 | 268 |
|  |  | Ct_Mle_06 | NCBI TSA | GFAT01121941.1 | 113 | 250 |
|  |  | Ct_Mle_07 | NCBI TSA | GFAT01021321.1 |  |  |
|  |  | Ct_Mle_08 | NCBI TSA | GFAT01080557.1 |  |  |
|  |  | Ct_Mle_09 | NCBI TSA | GFAT01054502.1 |  |  |
|  |  | Ct_Mle_10 | NCBI TSA | GFAT01043162.1 |  |  |
|  | *Beroe forskalii* | Ct_Bfo_01 | NCBI TSA | GHXY01066206.1 |  |  |
|  |  | Ct_Bfo_02 | NCBI TSA | GHXY01167744.1 |  |  |
|  |  | Ct_Bfo_03 | NCBI TSA | GHXY01095479.1 | 45 | 254 |
|  |  | Ct_Bfo_04 | NCBI TSA | GHXY01306105.1 |  |  |
|  |  | Ct_Bfo_05 | NCBI TSA | GHXY01122284.1 |  | 265 |
|  |  | Ct_Bfo_06 | NCBI TSA | GHXY01246062.1 | 96 |  |
|  |  | Ct_Bfo_07 | NCBI TSA | GHXY01206238.1 | 37 | 242 |
|  |  | Ct_Bfo_08 | NCBI TSA | GHXY01236032.1 |  |  |
|  |  | Ct_Bfo_09 | NCBI TSA | GHXY01235258.1 |  |  |
|  |  | Ct_Bfo_10 | NCBI TSA | GHXY01019817.1 |  |  |

| **Cnidarians** | *Hydra vulgaris* | Cn_Hvu_01 | UniProt | A0A0H5FNA2 |  | N248 |
| --- | --- | --- | --- | --- | --- | --- |
|  |  | Cn_Hvu_02 | UniProt | A0A5B8ICA4 | N81 |  |
|  |  | Cn_Hvu_03 | UniProt | A0A5B8HTC1 |  |  |
|  |  | Cn_Hvu_04 | UniProt | A0A0H5FN97 | N82 |  |
|  |  | Cn_Hvu_05 | UniProt | A0A5B8IM55 |  | N236 |
|  |  | Cn_Hvu_06 | UniProt | A0A5B8IDH4 | N84 N119 | N221 |
|  |  | Cn_Hvu_07 | UniProt | A0A5B8HUL2 |  |  |
|  |  | Cn_Hvu_08 | UniProt | A0A5B8IFJ1 |  | N242 |
|  |  | Cn_Hvu_09 | UniProt | A0A5B8IM45 | N83 | N237 |
|  |  | Cn_Hvu_10 | UniProt | A0A0H5FMF0 |  |  |
|  |  | Cn_Hvu_11 | UniProt | A0A5B8IE58 | N60 |  |
|  |  | Cn_Hvu_12 | UniProt | A0A5B8IC97 |  |  |
|  |  | Cn_Hvu_13 | UniProt | A0A5B8IM66 | N87 | N242 |
|  |  | Cn_Hvu_14 | UniProt | A0A5B8HVL4 | N81 | N232 |
|  |  | Cn_Hvu_15 | UniProt | A0A5B8HTC8 |  |  |
|  |  | Cn_Hvu_16 | UniProt | A0A5B8HVK5 | N102 N108 |  |
|  |  | Cn_Hvu_17 | UniProt | A0A5B8IFV2 | N51 | N250 |
|  |  | Cn_Hvu_18 | UniProt | A0A5B8IE64 |  |  |
|  |  | Cn_Hvu_19 | UniProt | Q2EMV6 | N49 |  |
|  | *Clytia hemisphaerica* | Cn_Che_01 | Marimba genome database | TCONS_00000247 |  |  |
|  |  | Cn_Che_02 | Marimba genome database | TCONS_00001207 |  |  |
|  |  | Cn_Che_03 | Marimba genome database | TCONS_00003109 | N53 |  |
|  |  | Cn_Che_04 | Marimba genome database | TCONS_00003336 |  |  |
|  |  | Cn_Che_05 | Marimba genome database | TCONS_00010894 | N102 |  |
|  |  | Cn_Che_06 | Marimba genome database | TCONS_00011573 |  |  |
|  |  | Cn_Che_07 | Marimba genome database | TCONS_00011578 |  |  |
|  |  | Cn_Che_08 | Marimba genome database | TCONS_00017253 |  |  |
|  |  | Cn_Che_09 | Marimba genome database | TCONS_00017255 |  |  |
|  |  | Cn_Che_10 | Marimba genome database | TCONS_00018944 |  |  |
|  |  | Cn_Che_11 | Marimba genome database | TCONS_00024463 |  |  |
|  |  | Cn_Che_12 | Marimba genome database | TCONS_00024465 |  |  |
|  |  | Cn_Che_13 | Marimba genome database | TCONS_00025865 |  |  |
|  |  | Cn_Che_14 | Marimba genome database | TCONS_00029525 | N87 |  |
|  |  | Cn_Che_15 | Marimba genome database | TCONS_00029535 | N91 |  |
|  |  | Cn_Che_16 | Marimba genome database | TCONS_00031259 |  |  |
|  |  | Cn_Che_17 | Marimba genome database | TCONS_00031261 |  | N285 |
|  |  | Cn_Che_18 | Marimba genome database | TCONS_00044548 |  |  |
|  |  | Cn_Che_19 | Marimba genome database | TCONS_00058825 |  |  |
|  |  | Cn_Che_20 | Marimba genome database | TCONS_00059268 |  |  |
|  |  | Cn_Che_21 | Marimba genome database | TCONS_00062505 | N44 |  |
|  |  | Cn_Che_22 | Marimba genome database | TCONS_00062506 |  |  |
|  |  | Cn_Che_23 | Marimba genome database | TCONS_00062507 |  |  |
|  |  | Cn_Che_24 | Marimba genome database | TCONS_00062512 |  |  |
|  |  | Cn_Che_25 | Marimba genome database | TCONS_00069173 |  |  |
|  |  | Cn_Che_26 | Marimba genome database | TCONS_00069175 |  |  |
|  |  | Cn_Che_27 | Marimba genome database | TCONS_00072805 |  |  |
|  | *Nematostella vectensis* | Cn_Nve_01 | UniProt | A7SWW1 | N62 |  |
|  |  | Cn_Nve_02 | UniProt | A7SYJ2 | N75 | N221 N235 |
|  | *Exaiptasia diaphana* | Cn_Edi_01 | NCBI | XP_020904565.1 | N53 N58 |  |
|  |  | Cn_Edi_02 | NCBI | XP_020900435.1 | N74 |  |
|  | *Actinia tenebrosa* | Cn_Ate_01 | UniProt | A0A6P8HQY0 | N80 |  |
|  |  | Cn_Ate_02 | UniProt | A0A6P8HHL7 | N61 N82 |  |
|  | *Pocillopora damicornis* | Cn_Pda_01 | UniProt | A0A3M6UTQ1 | N57 |  |
|  | *Siderastrea siderea* | Cn_Ssi_01 | NCBI TSA | GIYO011393513.1 |  |  |
|  |  | Cn_Ssi_02 | NCBI TSA | GIYO011129280.1 |  |  |
|  | *Eleutherobia rubra* | Cn_Eru_01 | NCBI TSA | GHFI01133402.1 |  |  |
|  |  | Cn_Eru_02 | NCBI TSA | GHFI01002934.1 |  | N239 |
|  | *Craterolophus convolvulus* | Cn_Cco_01 | NCBI TSA | HAGZ01035836.1 |  |  |
|  |  | Cn_Cco_02 | NCBI TSA | HAGZ01056096.1 |  |  |
|  | *Heliopora coerulea* | Cn_Hco_01 | NCBI TSA | GFVH01062719.1 |  | N211 |
|  |  | Cn_Hco_02 | NCBI TSA | GFVH01011767.1 |  |  |
|  | Edwardsiella carnea | Cn_Eca_01 | NCBI TSA | GGGD01237239.1 |  | N167 |
|  |  | Cn_Eca_02 | NCBI TSA | GGGB01116366.1 |  | N250 |
|  | Millepora complanata | Cn_Mco_01 | NCBI TSA | GFGT01166911.1 |  |  |
|  |  | Cn_Mco_02 | NCBI TSA | GFGT01165632.1 |  |  |
|  |  | Cn_Mco_03 | NCBI TSA | GIXC01109936.1 |  |  |
|  |  | Cn_Mco_04 | NCBI TSA | GFGT01268751.1 |  | N237 |
|  |  | Cn_Mco_05 | NCBI TSA | GIXC01116098.1 | N42 |  |
|  |  | Cn_Mco_06 | NCBI TSA | GFGT01088465.1 | N95 |  |
|  |  | Cn_Mco_07 | NCBI TSA | GIXC01078866.1 |  | N232 N237 |
|  |  | Cn_Mco_08 | NCBI TSA | GFGT01270854.1 |  |  |
|  |  | Cn_Mco_09 | NCBI TSA | GFGT01258618.1 |  | N237 |
|  |  | Cn_Mco_10 | NCBI TSA | GIXC01049302.1 |  |  |
|  | Millepora alcicornis | Cn_Mal_01 | NCBI TSA | GFAS01149004.1 |  |  |
|  |  | Cn_Mal_02 | NCBI TSA | GFAS01300238.1 |  |  |
|  |  | Cn_Mal_03 | NCBI TSA | GFAS01266418.1 |  |  |
|  |  | Cn_Mal_04 | NCBI TSA | GFAS01264651.1 |  |  |
|  |  | Cn_Mal_05 | NCBI TSA | GFAS01264650.1 |  |  |
|  |  | Cn_Mal_06 | NCBI TSA | GFAS01150958.1 | N42 |  |
|  |  | Cn_Mal_07 | NCBI TSA | GFAS01158523.1 | N95 |  |
|  |  | Cn_Mal_08 | NCBI TSA | GFAS01265729.1 |  | N237 |
|  |  | Cn_Mal_09 | NCBI TSA | GFAS01367969.1 |  | N240 |

| **Molluscs** | *Lottia gigantea* | Mo_Lgi_01 | UniProt | V3ZUL3 |  |  |
| --- | --- | --- | --- | --- | --- | --- |
|  |  | Mo_Lgi_02 | UniProt | V4BES3 |  |  |
|  |  | Mo_Lgi_03 | UniProt | V3Z652 |  |  |
|  |  | Mo_Lgi_04 | UniProt | V3ZZ22 |  |  |
|  |  | Mo_Lgi_05 | UniProt | V4CFP8 |  |  |
|  |  | Mo_Lgi_06 | UniProt | V3ZB67 |  |  |
|  |  | Mo_Lgi_07 | UniProt | V4B596 | N43 |  |
|  |  | Mo_Lgi_08 | UniProt | V4B6S1 |  |  |
|  |  | Mo_Lgi_09 | UniProt | V4AP14 | N79 | N201 |
|  |  | Mo_Lgi_10 | UniProt | V4B7P6 | N80 |  |
|  |  | Mo_Lgi_11 | UniProt | V3ZQ73 |  | N189 |
|  | *Elysia chlorotica* | Mo_Ech_01 | UniProt | A0A433U5C1 |  |  |
|  |  | Mo_Ech_02 | UniProt | A0A3S1HEP5 |  |  |
|  |  | Mo_Ech_03 | UniProt | A0A433TMI6 |  |  |
|  |  | Mo_Ech_04 | UniProt | A0A433SJ02 |  |  |
|  |  | Mo_Ech_05 | UniProt | A0A3S1AXY1 |  |  |
|  |  | Mo_Ech_06 | UniProt | A0A433SLC1 | N103 |  |
|  |  | Mo_Ech_07 | UniProt | A0A3S0ZM93 | N60 |  |
|  | *Arion vulgaris* | Mo_Avu_01 | UniProt | A0A0B7AXD1 |  |  |
|  |  | Mo_Avu_02 | UniProt | A0A0B6ZJX8 |  |  |
|  |  | Mo_Avu_03 | UniProt | A0A0B7A580 | N96 | N286 |
|  |  | Mo_Avu_04 | UniProt | A0A6C0X763 |  |  |
|  | *Lymnaea stagnalis* | Mo_Lst_01 | UniProt | A0A6C0X6W2 |  |  |
|  |  | Mo_Lst_02 | UniProt | A0A6C0X6V8 | N90 |  |
|  |  | Mo_Lst_03 | UniProt | A0A6C0X6W3 | N63 |  |
|  |  | Mo_Lst_04 | UniProt | A0A6C0X756 |  |  |
|  |  | Mo_Lst_05 | UniProt | A0A6C0X7M7 |  |  |
|  |  | Mo_Lst_06 | UniProt | A0A6C0XAN5 |  |  |
|  |  | Mo_Lst_07 | UniProt | A0A6C0X6X8 |  |  |
|  | *Biomphalaria glabrata* | Mo_Bgl_01 | UniProt | A0A2C9JXW7 | N90 |  |
|  |  | Mo_Bgl_02 | UniProt | A0A2C9K9P9 |  | N270 |
|  |  | Mo_Bgl_03 | UniProt | A0A2C9K256 | N63 |  |
|  |  | Mo_Bgl_04 | UniProt | A0A2C9K9P1 |  | N252 |
|  |  | Mo_Bgl_05 | UniProt | A0A2C9K7D5 |  |  |
|  |  | Mo_Bgl_06 | UniProt | A0A2C9K7D2 |  |  |
|  |  | Mo_Bgl_07 | UniProt | A0A2C9JTY8 |  |  |
|  |  | Mo_Bgl_08 | UniProt | A0A2C9K7H8 |  |  |
|  |  | Mo_Bgl_09 | UniProt | A0A2C9K9V1 |  | N250 |
|  |  | Mo_Bgl_10 | UniProt | A0A2C9K9N7 |  | N251 |
|  |  | Mo_Bgl_11 | UniProt | A0A2C9K9P0 |  | N270 |
|  |  | Mo_Bgl_12 | UniProt | A0A2C9JQ58 |  |  |
|  |  | Mo_Bgl_13 | UniProt | A0A2C9JTY6 |  | N280 |
|  |  | Mo_Bgl_14 | UniProt | A0A2C9JQ78 |  |  |
|  |  | Mo_Bgl_15 | UniProt | A0A2C9K9P4 |  | N262 |
|  |  | Mo_Bgl_16 | UniProt | A0A2C9JTY7 |  | N274 |
|  | *Aplysia californica* | Mo_Aca_01 | UniProt | Q29ZM7 |  | N277 |
|  |  | Mo_Aca_02 | UniProt | Q29ZM8 |  |  |
|  |  | Mo_Aca_03 | UniProt | Q4VTM8 |  |  |
|  |  | Mo_Aca_04 | UniProt | Q4VTM7 |  |  |
|  |  | Mo_Aca_05 | UniProt | Q2VTF0 |  |  |
|  |  | Mo_Aca_06 | UniProt | Q2VTE9 |  |  |
|  | *Pomacea canaliculata* | Mo_Pca_01 | UniProt | A0A2T7PX60 |  |  |
|  |  | Mo_Pca_02 | UniProt | A0A2T7Q090 |  |  |
|  |  | Mo_Pca_03 | UniProt | A0A2T7PCW5 | N86 N91 |  |
|  |  | Mo_Pca_04 | UniProt | A0A2T7PCW7 | N87 N92 |  |
|  | *Mizuhopecten yessoensis* | Mo_Mye_01 | UniProt | A0A210QU16 |  |  |
|  |  | Mo_Mye_02 | UniProt | A0A210QKE5 |  |  |
|  |  | Mo_Mye_03 | UniProt | A0A210QP80 |  |  |
|  |  | Mo_Mye_04 | UniProt | A0A210Q0W3 |  |  |
|  |  | Mo_Mye_05 | UniProt | A0A210QKJ5 |  |  |
|  |  | Mo_Mye_06 | UniProt | A0A210Q013 |  |  |
|  |  | Mo_Mye_07 | UniProt | A0A210PLR5 |  | N220 N259 |
|  |  | Mo_Mye_08 | UniProt | A0A210PFY8 |  |  |
|  |  | Mo_Mye_09 | UniProt | A0A210PLM9 | N47 | N208 N223 N262 |
|  |  | Mo_Mye_10 | UniProt | A0A210QY59 |  |  |
|  |  | Mo_Mye_11 | UniProt | A0A210QKF8 |  |  |
|  |  | Mo_Mye_12 | UniProt | A0A210Q038 |  |  |
|  |  | Mo_Mye_13 | UniProt | A0A210Q015 |  |  |
|  |  | Mo_Mye_14 | UniProt | A0A210Q0V7 |  |  |
|  |  | Mo_Mye_15 | UniProt | A0A210Q0T3 |  |  |
|  |  | Mo_Mye_16 | UniProt | A0A210Q0Y0 |  |  |
|  |  | Mo_Mye_17 | UniProt | A0A210QKF3 |  |  |
|  |  | Mo_Mye_18 | UniProt | A0A210Q018 |  |  |
|  |  | Mo_Mye_19 | UniProt | A0A210R591 |  | N267 N282 N321 |
|  | *Mytilus coruscus* | Mo_Mco_01 | UniProt | A0A6J8A5F4 | N496 |  |
|  |  | Mo_Mco_02 | UniProt | A0A6J8A5X3 |  |  |
|  |  | Mo_Mco_03 | UniProt | A0A6J8ATL8 |  |  |
|  |  | Mo_Mco_04 | UniProt | A0A6J8AI89 |  | N228 |
|  |  | Mo_Mco_05 | UniProt | A0A6J8A2Y9 |  |  |
|  |  | Mo_Mco_06 | UniProt | A0A6J8EIE0 | N44 N58 | N229 N257 |
|  |  | Mo_Mco_07 | UniProt | A0A6J8DQI7 |  |  |
|  |  | Mo_Mco_08 | UniProt | A0A6J8AU36 |  |  |
|  |  | Mo_Mco_09 | UniProt | A0A6J8E211 |  |  |
|  |  | Mo_Mco_10 | UniProt | A0A6J8BWJ7 |  |  |
|  |  | Mo_Mco_11 | UniProt | A0A6J8A627 | N89 |  |
|  |  | Mo_Mco_12 | UniProt | A0A6J8EAI6 |  | N228 |
|  |  | Mo_Mco_13 | UniProt | A0A6J8A621 |  |  |
|  |  | Mo_Mco_14 | UniProt | A0A6J8EAY9 |  |  |
|  |  | Mo_Mco_15 | UniProt | A0A6J8AV31 |  |  |
|  |  | Mo_Mco_16 | UniProt | A0A6J8AWM1 |  |  |
|  | *Crassostrea gigas* | Mo_Cgi_01 | UniProt | K1PJW3 |  |  |
|  |  | Mo_Cgi_02 | UniProt | K1QCA5 |  |  |
|  |  | Mo_Cgi_03 | UniProt | K1Q8X2 |  |  |
|  |  | Mo_Cgi_04 | UniProt | K1Q160 |  |  |
|  |  | Mo_Cgi_05 | UniProt | K1Q539 |  |  |
|  |  | Mo_Cgi_06 | UniProt | K1Q8H1 |  |  |
|  |  | Mo_Cgi_07 | UniProt | K1QFN4 |  |  |
|  |  | Mo_Cgi_08 | UniProt | K1PRS3 |  |  |
|  |  | Mo_Cgi_09 | UniProt | K1PTR0 |  |  |
|  |  | Mo_Cgi_10 | UniProt | K1QY06 | N95 |  |
|  |  | Mo_Cgi_11 | UniProt | K1PIY5 |  |  |
|  |  | Mo_Cgi_12 | UniProt | K1PRW3 |  |  |
|  |  | Mo_Cgi_13 | UniProt | K1RGD8 |  |  |
|  |  | Mo_Cgi_14 | UniProt | K1P371 |  |  |
|  | *Octopus bimaculoides* | Mo_Obi_01 | UniProt | A0A0L8HEL8 |  |  |
|  |  | Mo_Obi_02 | UniProt | A0A0L8G1Q2 |  |  |
|  |  | Mo_Obi_03 | UniProt | A0A0L8HEP5 |  |  |
|  |  | Mo_Obi_04 | UniProt | A0A0L8HEJ9 |  |  |
|  |  | Mo_Obi_05 | UniProt | A0A0L8GUL7 |  |  |
|  |  | Mo_Obi_06 | UniProt | A0A0L8HEI3 |  |  |
|  |  | Mo_Obi_07 | UniProt | A0A0L8HQL9 |  | N240 |
|  | *Octopus vulgaris* | Mo_Ovu_01 | UniProt | A0A6P7U9Y9 |  |  |
|  |  | Mo_Ovu_02 | UniProt | A0A6P7UB50 |  |  |
|  |  | Mo_Ovu_03 | UniProt | A0A6P7U709 |  |  |
|  |  | Mo_Ovu_04 | UniProt | A0A6P7U5G9 |  |  |
|  |  | Mo_Ovu_05 | UniProt | A0A6P7U5G7 |  |  |
|  |  | Mo_Ovu_06 | UniProt | A0A6P7U1Q6 |  |  |
|  |  | Mo_Ovu_07 | UniProt | A0A6P7U1Q1 |  |  |
|  |  | Mo_Ovu_08 | UniProt | A0A6P7U3T0 |  |  |
|  |  | Mo_Ovu_09 | UniProt | A0A6P7TWM1 |  |  |
|  |  | Mo_Ovu_10 | UniProt | A0A6P7TWL7 |  |  |
|  |  | Mo_Ovu_11 | UniProt | A0A6P7TWL4 |  |  |
|  |  | Mo_Ovu_12 | UniProt | A0A6P7U686 |  |  |
|  |  | Mo_Ovu_13 | UniProt | A0A6P7THX3 |  |  |
|  |  | Mo_Ovu_14 | UniProt | A0A6P7TEU0 |  |  |
|  |  | Mo_Ovu_15 | UniProt | A0A6P7UAA3 |  |  |
|  |  | Mo_Ovu_16 | UniProt | A0A6P7U2Y4 |  |  |
|  |  | Mo_Ovu_17 | UniProt | A0A6P7T9V3 |  |  |
|  |  | Mo_Ovu_18 | UniProt | A0A6P7TSX3 |  |  |
|  |  | Mo_Ovu_19 | UniProt | A0A6P7TS66 |  | N242 |
|  |  | Mo_Ovu_20 | UniProt | A0A6P7U9N6 |  |  |
|  |  | Mo_Ovu_21 | UniProt | A0A6P7TBX9 |  |  |

| **Annelids** | *Helobdella robusta* | An_Hro_01 | UniProt | T1FVB5 | N79 | N267 |
| --- | --- | --- | --- | --- | --- | --- |
|  |  | An_Hro_02 | UniProt | T1EE00 |  | N273 |
|  |  | An_Hro_03 | UniProt | T1ED09 |  |  |
|  |  | An_Hro_04 | UniProt | T1G9L4 |  |  |
|  |  | An_Hro_05 | UniProt | T1G942 |  |  |
|  |  | An_Hro_06 | UniProt | T1FF42 |  | N229 |
|  |  | An_Hro_07 | UniProt | T1G065 |  |  |
|  |  | An_Hro_08 | UniProt | T1FAN8 |  |  |
|  |  | An_Hro_09 | UniProt | T1EFQ7 |  |  |
|  |  | An_Hro_10 | UniProt | T1FN93 |  | N276 |
|  |  | An_Hro_11 | UniProt | T1FHL5 |  |  |
|  |  | An_Hro_12 | UniProt | T1F3K5 |  |  |
|  |  | An_Hro_13 | UniProt | T1G9C5 |  |  |
|  |  | An_Hro_14 | UniProt | T1ELD9 |  |  |
|  |  | An_Hro_15 | UniProt | T1G4P9 |  | N266 |
|  |  | An_Hro_16 | UniProt | T1G1G0 | N90 | N247 |
|  |  | An_Hro_17 | UniProt | T1FMB3 |  |  |
|  |  | An_Hro_18 | UniProt | T1EHZ6 |  |  |
|  |  | An_Hro_19 | UniProt | T1EHZ5 | N59 | N209 |
|  | *Hirudo medicinalis* | An_Hme_01 | UniProt | Q38HR5 | N78 N93 |  |
|  |  | An_Hme_02 | UniProt | Q38HR8 |  | N284 |
|  |  | An_Hme_03 | UniProt | Q38HR7 |  |  |
|  |  | An_Hme_04 | UniProt | Q38HR0 |  |  |
|  |  | An_Hme_05 | UniProt | Q8I6U2 |  |  |
|  |  | An_Hme_06 | UniProt | Q38HR6 |  | N236 |
|  |  | An_Hme_07 | UniProt | Q38HR2 |  |  |
|  |  | An_Hme_08 | UniProt | Q8I6U1 |  |  |
|  |  | An_Hme_09 | UniProt | Q38HQ9 |  |  |
|  | *Hirudo verbana* | An_Hve_01 | UniProt | H9C4Q5 |  | N272 N390 N401 |
|  |  | An_Hve_02 | UniProt | H9C4Q4 | N78 N93 |  |
|  |  | An_Hve_03 | UniProt | H9C4Q1 |  | N284 |
|  |  | An_Hve_04 | UniProt | H9C4R1 |  |  |
|  |  | An_Hve_05 | UniProt | H9C4R3 | N83 |  |
|  |  | An_Hve_06 | UniProt | H9C4R4 |  |  |
|  |  | An_Hve_07 | UniProt | H9C4R0 |  |  |
|  |  | An_Hve_08 | UniProt | H9C4Q2 |  |  |
|  |  | An_Hve_09 | UniProt | H9C4R8 |  |  |
|  |  | An_Hve_10 | UniProt | H9C4Q8 |  |  |
|  |  | An_Hve_11 | UniProt | H9C4P9 |  |  |
|  |  | An_Hve_12 | UniProt | H9C4R9 |  |  |
|  |  | An_Hve_13 | UniProt | H9C4R6 |  |  |
|  |  | An_Hve_14 | UniProt | H9C4Q3 |  | N236 |
|  |  | An_Hve_15 | UniProt | H9C4R7 |  |  |
|  |  | An_Hve_16 | UniProt | H9C4Q7 |  |  |
|  |  | An_Hve_17 | UniProt | H9C4Q6 |  | N269 |
|  |  | An_Hve_18 | UniProt | H9C4R5 |  | N270 |
|  |  | An_Hve_19 | UniProt | H9C4Q9 |  |  |
|  |  | An_Hve_20 | UniProt | H9C4Q0 |  |  |
|  |  | An_Hve_21 | UniProt | H9C4R2 |  | N242 |
|  | *Capitella teleta* | An_Cte_01 | UniProt | R7VAX0 | N74 | N248 |
|  |  | An_Cte_02 | UniProt | R7TDU4 | N73 | N228 |
|  |  | An_Cte_03 | UniProt | R7T8F9 |  |  |
|  |  | An_Cte_04 | UniProt | R7TLR9 |  |  |
|  |  | An_Cte_05 | UniProt | R7TQK9 |  |  |
|  |  | An_Cte_06 | UniProt | R7T7A0 |  |  |
|  |  | An_Cte_07 | UniProt | R7UJV0 |  |  |
|  |  | An_Cte_08 | UniProt | R7VBV6 |  |  |
|  |  | An_Cte_09 | UniProt | R7TBJ6 |  |  |
|  |  | An_Cte_10 | UniProt | R7UTT6 |  |  |
|  |  | An_Cte_11 | UniProt | R7VJQ0 |  |  |
|  |  | An_Cte_12 | UniProt | R7VBF2 |  | N237 |
|  |  | An_Cte_13 | UniProt | R7UVR0 |  |  |
|  |  | An_Cte_14 | UniProt | R7TBW5 |  |  |
|  |  | An_Cte_15 | UniProt | R7ULH1 |  |  |
|  |  | An_Cte_16 | UniProt | R7V9G1 |  |  |
|  |  | An_Cte_17 | UniProt | R7VJN8 | N66 | N237 |
|  |  | An_Cte_18 | UniProt | R7VBR0 |  | N237 |
|  | *Dimorphilus gyrociliatus* | An_Dgy_01 | NCBI | CAD5119303.1 |  |  |
|  |  | An_Dgy_02 | NCBI | CAD5113738.1 |  |  |
|  |  | An_Dgy_03 | NCBI | CAD5124943.1 |  |  |
|  |  | An_Dgy_04 | NCBI | CAD5113458.1 |  |  |
|  |  | An_Dgy_05 | NCBI | CAD5126531.1 |  |  |
|  |  | An_Dgy_06 | NCBI | CAD5119206.1 | N74 |  |
|  |  | An_Dgy_07 | NCBI | CAD5112293.1 |  |  |
|  |  | An_Dgy_08 | NCBI | CAD5126052.1 |  |  |
|  |  | An_Dgy_09 | NCBI | CAD5111819.1 |  |  |
|  |  | An_Dgy_10 | NCBI | CAD5118918.1 |  |  |
|  |  | An_Dgy_11 | NCBI | CAD5124621.1 |  |  |
|  |  | An_Dgy_12 | NCBI | CAD5124521.1 |  |  |
|  |  | An_Dgy_13 | NCBI | CAD5126051.1 |  | N236 |
|  |  | An_Dgy_14 | NCBI | CAD5113443.1 |  |  |
|  |  | An_Dgy_15 | NCBI | CAD5111855.1 |  |  |
|  |  | An_Dgy_16 | NCBI | CAD5111258.1 |  |  |
|  |  | An_Dgy_17 | NCBI | CAD5118919.1 |  | N226 |
|  |  | An_Dgy_18 | NCBI | CAD5120958.1 |  |  |
|  |  | An_Dgy_19 | NCBI | CAD5123465.1 |  |  |
|  |  | An_Dgy_20 | NCBI | CAD5116783.1 |  |  |

| **Plathyhelminthes** | *Clonorchis sinensis* | Pl_Csi_01 | UniProt | A0A3R7H257 |  |  |
| --- | --- | --- | --- | --- | --- | --- |
|  |  | Pl_Csi_02 | UniProt | G7Y9E1 |  | N230 |
|  |  | Pl_Csi_03 | UniProt | G7YWN9 |  |  |
|  |  | Pl_Csi_04 | UniProt | A0A3R7CWM9 |  |  |
|  |  | Pl_Csi_05 | UniProt | G7YU10 |  |  |
|  |  | Pl_Csi_06 | UniProt | H2KV19 |  | N238 |
|  |  | Pl_Csi_07 | UniProt | G7Y5Y1 |  |  |
|  |  | Pl_Csi_08 | UniProt | G7YTE6 |  |  |
|  |  | Pl_Csi_09 | UniProt | A0A419PPW6 |  |  |
|  |  | Pl_Csi_10 | UniProt | G7YNX9 |  |  |
|  |  | Pl_Csi_11 | UniProt | H2KP29 | N78 | N262 |
|  |  | Pl_Csi_12 | UniProt | H2KP32 | N84 |  |
|  |  | Pl_Csi_13 | UniProt | G7YGP3 |  |  |
|  | *Opisthorchis felineus* | Pl_Ofe_01 | UniProt | A0A4S2MGE2 |  |  |
|  |  | Pl_Ofe_02 | UniProt | A0A4S2LLY4 |  | N234 |
|  |  | Pl_Ofe_03 | UniProt | A0A4S2MFV9 |  |  |
|  |  | Pl_Ofe_04 | UniProt | A0A4S2LNS4 |  | N220 |
|  |  | Pl_Ofe_05 | UniProt | A0A4S2M9Y6 |  |  |
|  |  | Pl_Ofe_06 | UniProt | A0A4S2LZ48 |  |  |
|  |  | Pl_Ofe_07 | UniProt | A0A4S2LMZ1 |  |  |
|  |  | Pl_Ofe_08 | UniProt | A0A4S2LTJ3 | N84 |  |
|  |  | Pl_Ofe_09 | UniProt | A0A4S2LXE0 |  | N228 N283 |
|  |  | Pl_Ofe_10 | UniProt | A0A4S2LT02 | N78 | N262 |
|  |  | Pl_Ofe_11 | UniProt | A0A4S2LM37 |  |  |
|  |  | Pl_Ofe_12 | UniProt | A0A4S2LMY6 |  |  |
|  |  | Pl_Ofe_13 | UniProt | A0A4S2LVS8 |  | N220 |
|  |  | Pl_Ofe_14 | UniProt | A0A4S2LXD4 |  | N491 |
|  |  | Pl_Ofe_15 | UniProt | A0A4S2M067 |  | N238 |
|  |  | Pl_Ofe_16 | UniProt | A0A4S2MA48 |  |  |
|  |  | Pl_Ofe_17 | UniProt | A0A4S2M8B5 |  |  |
|  | *Opisthorchis viverrini* | Pl_Ovi_01 | UniProt | A0A075AFI6 |  |  |
|  |  | Pl_Ovi_02 | UniProt | A0A074Z8T4 |  |  |
|  |  | Pl_Ovi_03 | UniProt | A0A074ZIA2 | N78 | N262 |
|  |  | Pl_Ovi_04 | UniProt | A0A074Z5D4 |  |  |
|  |  | Pl_Ovi_05 | UniProt | A0A074ZMI3 |  | N220 |
|  |  | Pl_Ovi_06 | UniProt | A0A1S8WYC1 |  |  |
|  |  | Pl_Ovi_07 | UniProt | A0A075ACT2 |  |  |
|  |  | Pl_Ovi_08 | UniProt | A0A074ZTZ3 |  | N220 |
|  |  | Pl_Ovi_09 | UniProt | A0A074ZHH4 |  | N419 |
|  |  | Pl_Ovi_10 | UniProt | A0A1S8WVZ6 |  |  |
|  |  | Pl_Ovi_11 | UniProt | A0A075A080 |  | N234 |
|  |  | Pl_Ovi_12 | UniProt | A0A1S8X698 | N84 |  |
|  |  | Pl_Ovi_13 | UniProt | A0A074ZK86 |  | N238 |
|  | *Echinostoma caproni* | Pl_Eca_01 | UniProt | A0A3P8HMG7 |  | N189 |
|  |  | Pl_Eca_02 | UniProt | A0A183B3W3 |  |  |
|  |  | Pl_Eca_03 | UniProt | A0A183AM14 |  | N220 |
|  | *Fasciola gigantica* | Pl_Fgi_01 | UniProt | A0A504Y6X7 |  |  |
|  |  | Pl_Fgi_02 | UniProt | A0A504Y5J8 | N84 |  |
|  |  | Pl_Fgi_03 | UniProt | A0A504YQY5 |  | N220 |
|  |  | Pl_Fgi_04 | UniProt | A0A504Z0Z1 |  |  |
|  |  | Pl_Fgi_05 | UniProt | A0A504YCS9 |  |  |
|  |  | Pl_Fgi_06 | UniProt | A0A504YVX0 |  |  |
|  | *Fasciola hepatica* | Pl_Fhe_01 | UniProt | A0A4E0RMZ1 |  | N220 |
|  |  | Pl_Fhe_02 | UniProt | A0A4E0RPY2 |  | N234 |
|  |  | Pl_Fhe_03 | UniProt | A0A4E0RW48 | N84 |  |
|  |  | Pl_Fhe_04 | UniProt | A0A4E0RY00 |  |  |
|  |  | Pl_Fhe_05 | UniProt | A0A4E0RM26 |  | N228 |
|  |  | Pl_Fhe_06 | UniProt | A0A4E0R9P5 |  | N220 |
|  |  | Pl_Fhe_07 | UniProt | A0A4E0RLV9 |  | N238 |
|  | *Paragonimus westermani* | Pl_Pwe_01 | UniProt | A0A5J4NC06 |  |  |
|  |  | Pl_Pwe_02 | UniProt | A0A5J4NZL2 |  |  |
|  |  | Pl_Pwe_03 | UniProt | A0A5J4NXS3 |  | N238 |
|  |  | Pl_Pwe_04 | UniProt | A0A5J4NGN9 | N84 |  |
|  |  | Pl_Pwe_05 | UniProt | A0A5J4NUC7 | N78 | N265 |
|  |  | Pl_Pwe_06 | UniProt | A0A5J4NWA9 |  |  |
|  |  | Pl_Pwe_07 | UniProt | A0A5J4P177 |  | N323 |
|  | *Schistosoma bovis* | Pl_Sbo_01 | UniProt | A0A430QPZ1 | N78 | N262 |
|  |  | Pl_Sbo_02 | UniProt | A0A430QG77 |  |  |
|  |  | Pl_Sbo_03 | UniProt | A0A430QL07 |  | N239 |
|  |  | Pl_Sbo_04 | UniProt | A0A430Q3T5 | N84 |  |
|  |  | Pl_Sbo_05 | UniProt | A0A430QR17 |  |  |
|  |  | Pl_Sbo_06 | UniProt | A0A430QDM4 |  | N176 |
|  |  | Pl_Sbo_07 | UniProt | A0A430QGB7 |  |  |
|  | *Hymenolepis diminuta* | Pl_Hdi_01 | UniProt | A0A564YPR7 | N92 |  |
|  |  | Pl_Hdi_02 | UniProt | A0A564XVC7 |  | N262 |
|  |  | Pl_Hdi_03 | UniProt | A0A158QE71 |  | N185 |
|  |  | Pl_Hdi_04 | UniProt | A0A0R3SD38 |  |  |
|  |  | Pl_Hdi_05 | UniProt | A0A0R3SQR4 | N84 |  |
|  |  | Pl_Hdi_06 | UniProt | A0A0R3SV36 | N78 |  |
|  |  | Pl_Hdi_07 | UniProt | A0A0R3SV62 |  | N260 |
|  |  | Pl_Hdi_08 | UniProt | A0A0R3SV61 | N81 | N231 |
|  |  | Pl_Hdi_09 | UniProt | A0A158QC67 |  |  |
|  |  | Pl_Hdi_10 | UniProt | A0A0R3SVX4 |  |  |
|  | *Rodentolepis nana* | Pl_Rna_01 | UniProt | A0A158QH74 | N64 |  |
|  |  | Pl_Rna_02 | UniProt | A0A0R3TDA8 |  |  |
|  |  | Pl_Rna_03 | UniProt | A0A0R3T1H7 | N78 |  |
|  |  | Pl_Rna_04 | UniProt | A0A158QHF3 |  | N228 |
|  |  | Pl_Rna_05 | UniProt | A0A0R3TKC3 |  |  |
|  |  | Pl_Rna_06 | UniProt | A0A0R3TWI3 | N63 N98 | N260 |
|  |  | Pl_Rna_07 | UniProt | A0A0R3T064 | N84 |  |
|  | *Mesocestoides corti* | Pl_Mco_01 | UniProt | A0A0R3UAK9 | N78 |  |
|  |  | Pl_Mco_02 | UniProt | A0A158QU82 |  |  |
|  |  | Pl_Mco_03 | UniProt | A0A158QT43 |  |  |
|  |  | Pl_Mco_04 | UniProt | A0A0R3UAJ2 | N84 | N229 |
|  |  | Pl_Mco_05 | UniProt | A0A0R3UG40 |  |  |
|  |  | Pl_Mco_06 | UniProt | A0A0R3U5K7 |  |  |
|  |  | Pl_Mco_07 | UniProt | A0A5K3ENU4 |  | N260 |
|  |  | Pl_Mco_08 | UniProt | A0A3P6HXF4 |  |  |
|  |  | Pl_Mco_09 | UniProt | A0A0R3U3C1 |  | N238 |
|  |  | Pl_Mco_10 | UniProt | A0A0R3U194 |  | N180 |
|  | *Echinococcus multilocularis* | Pl_Emu_01 | UniProt | A0A068XY87 |  |  |
|  |  | Pl_Emu_02 | UniProt | A0A087W1T2 | N93 |  |
|  |  | Pl_Emu_03 | UniProt | A0A068YAQ8 |  |  |
|  |  | Pl_Emu_04 | UniProt | A0A068YAY3 |  | N228 |
|  |  | Pl_Emu_05 | UniProt | A0A068Y5E6 | N78 |  |
|  |  | Pl_Emu_06 | UniProt | A0A068YJC6 |  |  |
|  |  | Pl_Emu_07 | UniProt | A0A068XZR8 |  | N240 |
|  |  | Pl_Emu_08 | UniProt | A0A087W211 |  | N260 |
|  |  | Pl_Emu_09 | UniProt | A0A068Y3L2 | N90 | N241 |
|  |  | Pl_Emu_10 | UniProt | A0A087W1G9 |  | N220 |
|  |  | Pl_Emu_11 | UniProt | A0A068Y6C7 |  | N240 |
|  |  | Pl_Emu_12 | UniProt | A0A068Y626 | N97 | N248 N263 |
|  |  | Pl_Emu_13 | UniProt | A0A068Y4N3 | N92 | N238 |
|  | *Hydatigena taeniaeformis* | Pl_Hta_01 | UniProt | A0A0R3X7L4 | N93 |  |
|  |  | Pl_Hta_02 | UniProt | A0A0R3X485 |  | N192 |
|  |  | Pl_Hta_03 | UniProt | A0A0R3WPQ8 |  |  |
|  |  | Pl_Hta_04 | UniProt | A0A0R3WUZ7 | N97 |  |
|  |  | Pl_Hta_05 | UniProt | A0A0R3WKF9 | N84 |  |
|  |  | Pl_Hta_06 | UniProt | A0A0R3WK36 | N92 | N238 |
|  | *Dugesia japonica* | Pl_Dja_01 | UniProt | Q2L6M5 | N84 |  |
|  |  | Pl_Dja_02 | UniProt | Q2L6M9 |  | N220 |
|  |  | Pl_Dja_03 | UniProt | Q2L6M4 |  |  |
|  |  | Pl_Dja_04 | UniProt | Q2L6M3 |  |  |
|  |  | Pl_Dja_05 | UniProt | Q2L6N1 |  |  |
|  |  | Pl_Dja_06 | UniProt | Q2L6N0 |  |  |
|  |  | Pl_Dja_07 | UniProt | Q2L6M6 |  |  |
|  |  | Pl_Dja_08 | UniProt | I7FTZ1 |  |  |
|  |  | Pl_Dja_09 | UniProt | Q2L6M7 |  | N238 |
|  |  | Pl_Dja_10 | UniProt | Q2L6N2 |  | N220 |
|  | *Schmidtea mediterranea* | Pl_Sme_01 | UniProt | I1ZIL2 |  | N271 |
|  |  | Pl_Sme_02 | UniProt | A7RDN9 |  | N254 |
|  |  | Pl_Sme_03 | UniProt | H9CXU2 | N84 |  |
|  | *Cryptocotyle lingua* | Pl_Cli_01 | NCBI | QQY02540.1 |  |  |
|  |  | Pl_Cli_02 | NCBI | QQY02564.1 | N71 |  |
|  |  | Pl_Cli_03 | NCBI | QQY02485.1 |  |  |
|  |  | Pl_Cli_04 | NCBI | QQY02493.1 |  |  |
|  |  | Pl_Cli_05 | NCBI | QQY02558.1 |  |  |
|  |  | Pl_Cli_06 | NCBI | QQY02489.1 |  |  |
|  |  | Pl_Cli_07 | NCBI | QQY02533.1 |  | N241 |
|  |  | Pl_Cli_08 | NCBI | QQY02446.1 |  |  |
|  |  | Pl_Cli_09 | NCBI | QQY02615.1 |  |  |
|  |  | Pl_Cli_10 | NCBI | QQY02534.1 |  |  |
|  |  | Pl_Cli_11 | NCBI | QQY02568.1 |  |  |
|  |  | Pl_Cli_12 | NCBI | QQY02610.1 |  |  |
|  |  | Pl_Cli_13 | NCBI | QQY02611.1 |  |  |
|  | *Fasciolopsis buski* | Pl_Fbu_01 | NCBI | KAA0191062.1 |  |  |
|  |  | Pl_Fbu_02 | NCBI | VDP83634.1 |  |  |
|  |  | Pl_Fbu_03 | NCBI | KAA0199908.1 | N57 | N241 |
|  |  | Pl_Fbu_04 | NCBI | KAA0189704.1 |  |  |
|  |  | Pl_Fbu_05 | NCBI | VDP82532.1 |  |  |
|  |  | Pl_Fbu_06 | NCBI | VDP84140.1 | N44 | N226 |
|  |  | Pl_Fbu_07 | NCBI | KAA0184863.1 |  | N239 |
|  |  | Pl_Fbu_08 | NCBI | KAA0190542.1 |  |  |
|  |  | Pl_Fbu_09 | NCBI | VDP67763.1 |  |  |
|  |  | Pl_Fbu_10 | NCBI | VDP91171.1 |  |  |
|  |  | Pl_Fbu_11 | NCBI | KAA0200966.1 |  |  |
|  |  | Pl_Fbu_12 | NCBI | KAA0188566.1 | N63 |  |
|  |  | Pl_Fbu_13 | NCBI | KAA0186584.1 |  |  |
|  |  | Pl_Fbu_14 | NCBI | VDP70436.1 |  |  |
|  |  | Pl_Fbu_15 | NCBI | KAA0197213.1 |  |  |
|  | *Schistocephalus solidus* | Pl_Sso_01 | NCBI | VDL92445.1 |  |  |
|  |  | Pl_Sso_02 | NCBI | VDL99099.1 |  |  |
|  |  | Pl_Sso_03 | NCBI | VDL95133.1 |  |  |
|  |  | Pl_Sso_04 | NCBI | VDL98495.1 |  |  |
|  |  | Pl_Sso_05 | NCBI | VDL98447.1 | N63 |  |
|  |  | Pl_Sso_06 | NCBI | VDL95100.1 |  |  |
|  |  | Pl_Sso_07 | NCBI | VDL91104.1 |  |  |
|  |  | Pl_Sso_08 | NCBI | VDM02941.1 |  |  |
|  |  | Pl_Sso_09 | NCBI | VDL89333.1 |  |  |
|  |  | Pl_Sso_10 | NCBI | VDL95246.1 |  |  |
|  | *Sparganum proliferum* | Pl_Spr_01 | NCBI | VZI29331.1 |  |  |
|  |  | Pl_Spr_02 | NCBI | VZI03983.1 |  |  |
|  |  | Pl_Spr_03 | NCBI | VZI10700.1 |  |  |
|  |  | Pl_Spr_04 | NCBI | VZI10682.1 |  |  |
|  |  | Pl_Spr_05 | NCBI | VZI23197.1 |  |  |
|  |  | Pl_Spr_06 | NCBI | VZI34983.1 |  |  |
|  |  | Pl_Spr_07 | NCBI | VZH95804.1 | N116 |  |
|  |  | Pl_Spr_08 | NCBI | VZI31141.1 |  |  |
|  |  | Pl_Spr_09 | NCBI | VZI30066.1 |  |  |
|  |  | Pl_Spr_10 | NCBI | VZI33550.1 |  |  |
|  |  | Pl_Spr_11 | NCBI | VZI24029.1 |  |  |
|  |  | Pl_Spr_12 | NCBI | VZI34554.1 |  |  |
|  | *Taenia asiatica* | Pl_Tas_01 | NCBI | VDK33906.1 |  |  |
|  |  | Pl_Tas_02 | NCBI | VDK32103.1 |  |  |
|  |  | Pl_Tas_03 | NCBI | VDK33160.1 |  |  |
|  |  | Pl_Tas_04 | NCBI | VDK38412.1 |  |  |
|  |  | Pl_Tas_05 | NCBI | VDK32282.1 | N71 |  |
|  |  | Pl_Tas_06 | NCBI | VDK37644.1 |  |  |
|  |  | Pl_Tas_07 | NCBI | VDK23507.1 | N63 |  |
|  |  | Pl_Tas_08 | NCBI | VDK34661.1 |  |  |
|  |  | Pl_Tas_09 | NCBI | VDK25407.1 |  |  |
|  |  | Pl_Tas_10 | NCBI | VDK21681.1 | N57 |  |
|  |  | Pl_Tas_11 | NCBI | VDK33161.1 |  |  |
|  |  | Pl_Tas_12 | NCBI | VDK34188.1 |  |  |
|  |  | Pl_Tas_13 | NCBI | VDK26693.1 |  |  |
|  |  | Pl_Tas_14 | NCBI | VDK37646.1 |  |  |

| **Nematodes** | *Trichinella spiralis* | Ne_Tsp_01 | UniProt | A0A0V1AZN6 |  | N241 |
| --- | --- | --- | --- | --- | --- | --- |
|  |  | Ne_Tsp_02 | UniProt | A0A0V1B5C2 |  |  |
|  |  | Ne_Tsp_03 | UniProt | E5SC05 |  | N239 |
|  |  | Ne_Tsp_04 | UniProt | E5SKQ0 |  |  |
|  |  | Ne_Tsp_05 | UniProt | A0A0V1AXU9 | N686 |  |
|  |  | Ne_Tsp_06 | UniProt | A0A0V1C0M8 |  |  |
|  |  | Ne_Tsp_07 | UniProt | A0A0V1AQR6 |  |  |
|  |  | Ne_Tsp_08 | UniProt | A0A0V1AZ11 | N80 | N247 |
|  |  | Ne_Tsp_09 | UniProt | A0A0V1AVK6 |  |  |
|  |  | Ne_Tsp_10 | UniProt | A0A0V1BVK0 |  |  |
|  |  | Ne_Tsp_11 | UniProt | A0A0V1BBK8 |  |  |
|  | *Trichinella britovi* | Ne_Tbr_01 | UniProt | A0A0V1CV14 |  | N260 |
|  |  | Ne_Tbr_02 | UniProt | A0A0V1D3S0 |  | N241 |
|  |  | Ne_Tbr_03 | UniProt | A0A0V1DGJ5 |  |  |
|  |  | Ne_Tbr_04 | UniProt | A0A0V1CFF4 |  | N244 |
|  |  | Ne_Tbr_05 | UniProt | A0A0V1D5G7 |  |  |
|  |  | Ne_Tbr_06 | UniProt | A0A0V1DDE5 |  |  |
|  |  | Ne_Tbr_07 | UniProt | A0A0V1D5H4 |  |  |
|  |  | Ne_Tbr_08 | UniProt | A0A0V1CT34 |  |  |
|  |  | Ne_Tbr_09 | UniProt | A0A0V1DD77 |  |  |
|  |  | Ne_Tbr_10 | UniProt | A0A0V1CB37 | N80 | N224 N247 |
|  |  | Ne_Tbr_11 | UniProt | A0A0V1CU17 |  |  |
|  |  | Ne_Tbr_12 | UniProt | A0A0V1DIW6 |  |  |
|  |  | Ne_Tbr_13 | UniProt | A0A0V1CH91 |  |  |
|  | *Trichinella murrelli* | Ne_Tmr_01 | UniProt | A0A0V0TAA3 | N427 |  |
|  |  | Ne_Tmr_02 | UniProt | A0A0V0U3G8 |  | N400 |
|  |  | Ne_Tmr_03 | UniProt | A0A0V0TYW8 |  | N241 |
|  |  | Ne_Tmr_04 | UniProt | A0A0V0TKG9 |  |  |
|  |  | Ne_Tmr_05 | UniProt | A0A0V0T9L3 |  | N239 |
|  |  | Ne_Tmr_06 | UniProt | A0A0V0UC99 |  |  |
|  |  | Ne_Tmr_07 | UniProt | A0A0V0TYV3 |  |  |
|  |  | Ne_Tmr_08 | UniProt | A0A0V0T8N8 |  |  |
|  |  | Ne_Tmr_09 | UniProt | A0A0V0TFC5 | N80 | N247 |
|  |  | Ne_Tmr_10 | UniProt | A0A0V0TFB2 |  |  |
|  |  | Ne_Tmr_11 | UniProt | A0A0V0TMN6 |  |  |
|  | *Trichuris muris* | Ne_Tmu_01 | UniProt | A0A5S6QY30 |  |  |
|  |  | Ne_Tmu_02 | UniProt | A0A5S6QQ19 |  |  |
|  |  | Ne_Tmu_03 | UniProt | A0A5S6QVZ4 |  | N238 |
|  |  | Ne_Tmu_04 | UniProt | A0A5S6QZJ9 |  | N241 |
|  |  | Ne_Tmu_05 | UniProt | A0A5S6QHM8 |  |  |
|  |  | Ne_Tmu_06 | UniProt | A0A5S6QMQ5 |  |  |
|  |  | Ne_Tmu_07 | UniProt | A0A5S6QDG0 | N81 |  |
|  |  | Ne_Tmu_08 | UniProt | A0A5S6QT19 |  |  |
|  |  | Ne_Tmu_09 | UniProt | A0A5S6QLR1 |  |  |
|  |  | Ne_Tmu_10 | UniProt | A0A5S6QDB5 |  |  |
|  |  | Ne_Tmu_11 | UniProt | A0A5S6QH92 |  |  |
|  | *Trichuris trichiura* | Ne_Ttr_01 | UniProt | A0A077Z3F7 |  |  |
|  |  | Ne_Ttr_02 | UniProt | A0A077Z4T1 |  |  |
|  |  | Ne_Ttr_03 | UniProt | A0A077Z8F8 |  |  |
|  |  | Ne_Ttr_04 | UniProt | A0A077Z3E1 |  | N242 |
|  |  | Ne_Ttr_05 | UniProt | A0A077Z457 |  |  |
|  |  | Ne_Ttr_06 | UniProt | A0A077Z0H5 | N81 |  |
|  |  | Ne_Ttr_07 | UniProt | A0A077Z0Z3 |  |  |
|  |  | Ne_Ttr_08 | UniProt | A0A077Z654 |  |  |
|  |  | Ne_Ttr_09 | UniProt | A0A077ZBD9 |  |  |
|  |  | Ne_Ttr_10 | UniProt | A0A077Z931 |  |  |
|  | *Soboliphyme baturini* | Ne_Sba_01 | UniProt | A0A183IFX9 |  |  |
|  |  | Ne_Sba_02 | UniProt | A0A183INN5 |  | N237 |
|  |  | Ne_Sba_03 | UniProt | A0A183ITJ3 |  |  |
|  |  | Ne_Sba_04 | UniProt | A0A183IGA6 |  |  |
|  |  | Ne_Sba_05 | UniProt | A0A183IHM0 |  | N202 |
|  | *Caenorhabditis elegans* | Ne_Cel_01 | UniProt | Q22549 |  | N239 |
|  |  | Ne_Cel_02 | UniProt | Q21123 |  | N267 |
|  |  | Ne_Cel_03 | UniProt | Q03412 |  |  |
|  |  | Ne_Cel_04 | UniProt | Q23157 |  |  |
|  |  | Ne_Cel_05 | UniProt | O61715 |  |  |
|  |  | Ne_Cel_06 | UniProt | Q23027 |  |  |
|  |  | Ne_Cel_07 | UniProt | Q27295 |  |  |
|  |  | Ne_Cel_08 | UniProt | Q19746 |  | N219 |
|  |  | Ne_Cel_09 | UniProt | Q9U3K5 | N87 |  |
|  |  | Ne_Cel_10 | UniProt | O01634 | N99 |  |
|  |  | Ne_Cel_11 | UniProt | Q9U3N4 |  |  |
|  |  | Ne_Cel_12 | UniProt | O01393 |  | N223 |
|  |  | Ne_Cel_13 | UniProt | Q23593 |  |  |
|  |  | Ne_Cel_14 | UniProt | O61787 |  |  |
|  |  | Ne_Cel_15 | UniProt | O61788 | N81 | N250 |
|  | *Pristionchus pacificus* | Ne_Ppa_01 | UniProt | H3EMZ9 | N81 | N247 |
|  |  | Ne_Ppa_02 | UniProt | H3E6H0 |  |  |
|  |  | Ne_Ppa_03 | UniProt | H3EKV9 |  |  |
|  |  | Ne_Ppa_04 | UniProt | H3F590 |  | N229 |
|  |  | Ne_Ppa_05 | UniProt | H3EFE5 |  |  |
|  |  | Ne_Ppa_06 | UniProt | A0A4X3P932 |  |  |
|  |  | Ne_Ppa_07 | UniProt | H3F937 |  |  |
|  |  | Ne_Ppa_08 | UniProt | H3FA02 |  |  |
|  |  | Ne_Ppa_09 | UniProt | A0A4X3NYP8 | N206 |  |
|  |  | Ne_Ppa_10 | UniProt | A0A4X3NV72 |  | N254 |
|  |  | Ne_Ppa_11 | UniProt | H3FY67 |  |  |
|  |  | Ne_Ppa_12 | UniProt | A0A4X3PCB6 |  |  |
|  |  | Ne_Ppa_13 | UniProt | A0A4X3NXJ9 |  |  |
|  |  | Ne_Ppa_14 | UniProt | H3EHR6 | N100 |  |
|  |  | Ne_Ppa_15 | UniProt | H3EAV4 |  |  |
|  |  | Ne_Ppa_16 | UniProt | H3EHR4 |  |  |
|  |  | Ne_Ppa_17 | UniProt | H3EAW6 |  |  |
|  |  | Ne_Ppa_18 | UniProt | H3DU91 |  |  |
|  | *Caenorhabditis brenneri* | Ne_Cbr_01 | UniProt | G0NJM8 |  |  |
|  |  | Ne_Cbr_02 | UniProt | G0NVZ8 |  | N267 |
|  |  | Ne_Cbr_03 | UniProt | G0MB03 |  |  |
|  |  | Ne_Cbr_04 | UniProt | G0MFM2 |  |  |
|  |  | Ne_Cbr_05 | UniProt | G0P5C9 | N89 |  |
|  |  | Ne_Cbr_06 | UniProt | G0NJ70 |  |  |
|  |  | Ne_Cbr_07 | UniProt | G0NI48 |  |  |
|  |  | Ne_Cbr_08 | UniProt | G0N574 |  |  |
|  |  | Ne_Cbr_09 | UniProt | G0NKI0 |  |  |
|  |  | Ne_Cbr_10 | UniProt | G0MT10 |  | N219 |
|  |  | Ne_Cbr_11 | UniProt | G0M6M8 | N87 |  |
|  |  | Ne_Cbr_12 | UniProt | G0PCQ6 | N99 |  |
|  |  | Ne_Cbr_13 | UniProt | G0MH94 |  |  |
|  |  | Ne_Cbr_14 | UniProt | G0MBK9 |  | N223 |
|  |  | Ne_Cbr_15 | UniProt | G0NJH2 |  |  |
|  |  | Ne_Cbr_16 | UniProt | G0NJM9 |  |  |
|  |  | Ne_Cbr_17 | UniProt | G0PHX1 |  |  |
|  | *Dracunculus medinensis* | Ne_Dme_01 | UniProt | A0A0N4URD8 |  | N256 |
|  |  | Ne_Dme_02 | UniProt | A0A0N4UD17 |  |  |
|  |  | Ne_Dme_03 | UniProt | A0A0N4UJT0 |  |  |
|  |  | Ne_Dme_04 | UniProt | A0A158Q5J6 |  |  |
|  |  | Ne_Dme_05 | UniProt | A0A0N4UHQ9 |  | N260 |
|  |  | Ne_Dme_06 | UniProt | A0A0N4U0E9 |  | N238 |
|  |  | Ne_Dme_07 | UniProt | A0A158Q618 |  | N215 |
|  |  | Ne_Dme_08 | UniProt | A0A0N4UAF7 | N100 |  |
|  |  | Ne_Dme_09 | UniProt | A0A158Q694 |  |  |
|  |  | Ne_Dme_10 | UniProt | A0A0N4UAF6 |  |  |
|  |  | Ne_Dme_11 | UniProt | A0A0N4U9U2 |  | N249 |
|  |  | Ne_Dme_12 | UniProt | A0A0N4U4A2 |  |  |
|  |  | Ne_Dme_13 | UniProt | A0A0N4UQM8 |  |  |
|  | *Anisakis simplex* | Ne_Asi_01 | UniProt | A0A0M3JUH9 |  | N553 |
|  |  | Ne_Asi_02 | UniProt | A0A0M3JR29 |  |  |
|  |  | Ne_Asi_03 | UniProt | A0A158PP29 |  | N297 |
|  |  | Ne_Asi_04 | UniProt | A0A346RVN4 |  | N239 |
|  |  | Ne_Asi_05 | UniProt | A0A2R4KQZ7 |  |  |
|  |  | Ne_Asi_06 | UniProt | A0A346RVL7 |  |  |
|  |  | Ne_Asi_07 | UniProt | A0A0M3JR87 |  |  |
|  |  | Ne_Asi_08 | UniProt | A0A0M3JUS4 |  |  |
|  |  | Ne_Asi_09 | UniProt | A0A2R4KR07 |  |  |
|  |  | Ne_Asi_10 | UniProt | A0A0M3JU42 |  |  |
|  |  | Ne_Asi_11 | UniProt | A0A0M3JR88 |  | N253 |
|  | *Ascaris lumbricoides* | Ne_Alu_01 | UniProt | A0A0M3HT97 |  | N273 |
|  |  | Ne_Alu_02 | UniProt | A0A0M3HMV1 |  |  |
|  |  | Ne_Alu_03 | UniProt | A0A0M3I3S7 |  |  |
|  |  | Ne_Alu_04 | UniProt | A0A0M3HNG6 |  |  |
|  |  | Ne_Alu_05 | UniProt | A0A0M3HRM5 | N82 |  |
|  |  | Ne_Alu_06 | UniProt | A0A0M3HRI4 | N82 |  |
|  | *Enterobius vermicularis* | Ne_Eve_01 | UniProt | A0A0N4VHQ9 |  | N254 |
|  |  | Ne_Eve_02 | UniProt | A0A0N4UXC2 |  |  |
|  |  | Ne_Eve_03 | UniProt | A0A0N4VFE5 |  |  |
|  |  | Ne_Eve_04 | UniProt | A0A0N4VEZ3 |  | N273 N245 |
|  |  | Ne_Eve_05 | UniProt | A0A158QAD5 |  |  |
|  |  | Ne_Eve_06 | UniProt | A0A0N4V9Q0 |  |  |
|  |  | Ne_Eve_07 | UniProt | A0A0N4UWF5 | N65 N94 | N255 |
|  |  | Ne_Eve_08 | UniProt | A0A0N4VKQ4 |  |  |
|  |  | Ne_Eve_09 | UniProt | A0A0N4VBS4 |  |  |
|  |  | Ne_Eve_10 | UniProt | A0A0N4VAU6 |  | N234 |
|  |  | Ne_Eve_11 | UniProt | A0A0N4V5J5 |  |  |
|  |  | Ne_Eve_12 | UniProt | A0A0N4VBS3 |  |  |
|  | *Syphacia muris* | Ne_Smu_01 | UniProt | A0A0N5AKQ1 |  | N272 |
|  |  | Ne_Smu_02 | UniProt | A0A0N5AIV2 |  | N315 |
|  |  | Ne_Smu_03 | UniProt | A0A0N5AXY4 |  |  |
|  |  | Ne_Smu_04 | UniProt | A0A0N5A9L6 | N90 | N258 |
|  |  | Ne_Smu_05 | UniProt | A0A0N5A9F0 |  |  |
|  |  | Ne_Smu_06 | UniProt | A0A158R4F1 |  |  |
|  |  | Ne_Smu_07 | UniProt | A0A0N5AEF8 |  |  |
|  |  | Ne_Smu_08 | UniProt | A0A0N5ASH8 |  |  |
|  |  | Ne_Smu_09 | UniProt | A0A0N5B018 |  |  |
|  |  | Ne_Smu_10 | UniProt | A0A0N5AIU0 |  |  |
|  |  | Ne_Smu_11 | UniProt | A0A0N5AAY5 | N100 |  |
|  |  | Ne_Smu_12 | UniProt | A0A0N5AP55 |  |  |
|  |  | Ne_Smu_13 | UniProt | A0A0N5AYZ5 |  |  |
|  |  | Ne_Smu_14 | UniProt | A0A0N5AGQ2 |  |  |
|  |  | Ne_Smu_15 | UniProt | A0A0N5ABY8 |  | N274 |
|  |  | Ne_Smu_16 | UniProt | A0A0N5AYG4 |  |  |
|  | *Acanthocheilonema viteae* | Ne_Avi_01 | UniProt | A0A498SFZ8 |  | N241 |
|  |  | Ne_Avi_02 | UniProt | A0A498S8Z7 |  |  |
|  |  | Ne_Avi_03 | UniProt | A0A498SFZ6 |  | N243 |
|  |  | Ne_Avi_04 | UniProt | A0A498SB47 |  |  |
|  |  | Ne_Avi_05 | UniProt | A0A498S9K0 |  |  |
|  |  | Ne_Avi_06 | UniProt | A0A498S735 |  |  |
|  |  | Ne_Avi_07 | UniProt | A0A498SID0 |  |  |
|  |  | Ne_Avi_08 | UniProt | A0A498SFE7 |  |  |
|  |  | Ne_Avi_09 | UniProt | A0A498SDS8 |  | N188 |
|  |  | Ne_Avi_10 | UniProt | A0A498SPN9 |  |  |
|  | *Brugia pahangi* | Ne_Bpa_01 | UniProt | A0A158PPX0 |  |  |
|  |  | Ne_Bpa_02 | UniProt | A0A0N4SZM7 |  |  |
|  |  | Ne_Bpa_03 | UniProt | A0A158PQ75 |  | N286 |
|  |  | Ne_Bpa_04 | UniProt | A0A0N4TMZ6 |  |  |
|  |  | Ne_Bpa_05 | UniProt | A0A0N4TM67 |  |  |
|  |  | Ne_Bpa_06 | UniProt | A0A0N4TQI0 |  | N241 |
|  |  | Ne_Bpa_07 | UniProt | A0A3P7QDG4 |  |  |
|  |  | Ne_Bpa_08 | UniProt | A0A0N4TKT7 |  |  |
|  |  | Ne_Bpa_09 | UniProt | A0A0N4T2B3 |  |  |
|  |  | Ne_Bpa_10 | UniProt | A0A0N4SWM3 | N82 |  |
|  |  | Ne_Bpa_11 | UniProt | A0A0N4T3A6 | N87 |  |
|  |  | Ne_Bpa_12 | UniProt | A0A0N4TRN4 |  |  |
|  |  | Ne_Bpa_13 | UniProt | A0A0N4TPE5 |  |  |
|  |  | Ne_Bpa_14 | UniProt | A0A0N4TVC5 |  | N223 |
|  | *Elaeophora elaphi* | Ne_Eel_01 | UniProt | A0A0R3S611 |  |  |
|  |  | Ne_Eel_02 | UniProt | A0A0R3RFQ0 |  |  |
|  |  | Ne_Eel_03 | UniProt | A0A158Q7E7 |  | N239 |
|  |  | Ne_Eel_04 | UniProt | A0A0R3RI94 |  |  |
|  |  | Ne_Eel_05 | UniProt | A0A0R3RVD0 |  |  |
|  |  | Ne_Eel_06 | UniProt | A0A0R3RTU9 |  |  |
|  |  | Ne_Eel_07 | UniProt | A0A0R3RZF8 |  |  |
|  |  | Ne_Eel_08 | UniProt | A0A0R3S431 |  | N306 |
|  |  | Ne_Eel_09 | UniProt | A0A0R3S5N7 | N100 |  |
|  |  | Ne_Eel_10 | UniProt | A0A0R3RK80 |  |  |
|  |  | Ne_Eel_11 | UniProt | A0A0R3RI02 |  |  |
|  |  | Ne_Eel_12 | UniProt | A0A0R3RK83 | N85 |  |
|  |  | Ne_Eel_13 | UniProt | A0A0R3S3P1 |  | N221 |
|  |  | Ne_Eel_14 | UniProt | A0A0R3S688 |  |  |
|  | *Litomosoides sigmodontis* | Ne_Lsi_01 | UniProt | A0A3P6UKJ6 |  |  |
|  |  | Ne_Lsi_02 | UniProt | A0A3P6TA91 |  |  |
|  |  | Ne_Lsi_03 | UniProt | A0A3P6S074 |  | N239 |
|  |  | Ne_Lsi_04 | UniProt | A0A3P6THZ3 |  | N241 |
|  |  | Ne_Lsi_05 | UniProt | A0A3P6TBR4 |  |  |
|  |  | Ne_Lsi_06 | UniProt | A0A3P6TG21 | N137 |  |
|  |  | Ne_Lsi_07 | UniProt | A0A3P6SKB9 | N82 |  |
|  |  | Ne_Lsi_08 | UniProt | A0A3P6USD0 |  |  |
|  |  | Ne_Lsi_09 | UniProt | A0A3P6TNF5 |  |  |
|  |  | Ne_Lsi_10 | UniProt | A0A3P6U341 |  |  |
|  |  | Ne_Lsi_11 | UniProt | A0A3P6T8R0 |  |  |
|  |  | Ne_Lsi_12 | UniProt | A0A3P6SBR3 |  |  |
|  |  | Ne_Lsi_13 | UniProt | A0A3P6V9Y8 |  |  |
|  | *Loa loa* | Ne_Llo_01 | UniProt | A0A1S0TZW5 |  |  |
|  |  | Ne_Llo_02 | UniProt | A0A1S0U9U2 |  | N239 |
|  |  | Ne_Llo_03 | UniProt | A0A1I7VXQ4 |  |  |
|  |  | Ne_Llo_04 | UniProt | A0A1S0UB65 |  |  |
|  |  | Ne_Llo_05 | UniProt | A0A1S0UFK4 |  | N241 |
|  |  | Ne_Llo_06 | UniProt | A0A1I7VZJ3 |  | N240 |
|  |  | Ne_Llo_07 | UniProt | A0A1S0U3A9 |  |  |
|  |  | Ne_Llo_08 | UniProt | A0A1I7W157 |  |  |
|  |  | Ne_Llo_09 | UniProt | A0A1S0TM78 |  |  |
|  |  | Ne_Llo_10 | UniProt | A0A1I7VEN4 |  |  |
|  |  | Ne_Llo_11 | UniProt | A0A1I7VKW5 |  |  |
|  |  | Ne_Llo_12 | UniProt | A0A1S0TRP3 |  |  |
|  |  | Ne_Llo_13 | UniProt | A0A1I7VGB5 |  |  |
|  |  | Ne_Llo_14 | UniProt | A0A1I7VHP3 |  |  |
|  |  | Ne_Llo_15 | UniProt | A0A1I7V589 |  | N221 |
|  |  | Ne_Llo_16 | UniProt | A0A1S0TZ71 | N85 N97 |  |
|  |  | Ne_Llo_17 | UniProt | A0A1S0UFG2 |  |  |
|  |  | Ne_Llo_18 | UniProt | A0A1S0TVV2 | N82 |  |
|  |  | Ne_Llo_19 | UniProt | A0A1S0U6I5 |  | N179 |
|  | *Onchocerca flexuosa* | Ne_Ofl_01 | UniProt | A0A238BZB1 |  | N243 |
|  |  | Ne_Ofl_02 | UniProt | A0A183H2Q8 |  |  |
|  |  | Ne_Ofl_03 | UniProt | A0A238BZW8 |  |  |
|  |  | Ne_Ofl_04 | UniProt | A0A183H6N1 |  |  |
|  |  | Ne_Ofl_05 | UniProt | A0A238BV49 |  |  |
|  |  | Ne_Ofl_06 | UniProt | A0A238BZU9 |  |  |
|  |  | Ne_Ofl_07 | UniProt | A0A183I013 |  |  |
|  |  | Ne_Ofl_08 | UniProt | A0A183I3U9 |  |  |
|  |  | Ne_Ofl_09 | UniProt | A0A238BT96 |  | N239 |
|  |  | Ne_Ofl_10 | UniProt | A0A238C5U0 |  |  |
|  |  | Ne_Ofl_11 | UniProt | A0A183H3Q4 |  |  |
|  |  | Ne_Ofl_12 | UniProt | A0A183HYN5 |  |  |
|  |  | Ne_Ofl_13 | UniProt | A0A183I1C0 |  | N239 |
|  |  | Ne_Ofl_14 | UniProt | A0A238BQ43 |  | N241 |
|  | *Wuchereria bancrofti* | Ne_Wba_01 | UniProt | A0A1I8EQL9 | N100 |  |
|  |  | Ne_Wba_02 | UniProt | J9FAW5 |  |  |
|  |  | Ne_Wba_03 | UniProt | J9EMK4 |  | N294 |
|  |  | Ne_Wba_04 | UniProt | A0A1I8EM42 |  | N243 |
|  |  | Ne_Wba_05 | UniProt | J9F0N4 |  |  |
|  |  | Ne_Wba_06 | UniProt | J9BJK4 |  |  |
|  |  | Ne_Wba_07 | UniProt | J9FFN8 |  | N272 |
|  |  | Ne_Wba_08 | UniProt | J9BFB6 |  |  |
|  |  | Ne_Wba_09 | UniProt | J9B6M7 |  |  |
|  |  | Ne_Wba_10 | UniProt | J9EZB8 | N82 |  |
|  |  | Ne_Wba_11 | UniProt | J9EPQ3 |  |  |
|  |  | Ne_Wba_12 | UniProt | A0A1I8EBF1 | N87 |  |
|  |  | Ne_Wba_13 | UniProt | J9EKZ3 |  |  |
|  |  | Ne_Wba_14 | UniProt | A0A3P7E2W3 |  | N241 |
|  | *Steinernema carpocapsae* | Ne_Sca_01 | UniProt | A0A4U5LXJ6 |  |  |
|  |  | Ne_Sca_02 | UniProt | A0A4U8UTT7 |  |  |
|  |  | Ne_Sca_03 | UniProt | A0A4U5MSH5 |  |  |
|  |  | Ne_Sca_04 | UniProt | A0A4U5NZ08 |  | N231 |
|  |  | Ne_Sca_05 | UniProt | A0A4U5PC08 |  |  |
|  |  | Ne_Sca_06 | UniProt | A0A4U8UUS7 |  |  |
|  |  | Ne_Sca_07 | UniProt | A0A4V6A677 |  |  |
|  |  | Ne_Sca_08 | UniProt | A0A4U8V1A0 |  |  |
|  |  | Ne_Sca_09 | UniProt | A0A4V6A669 |  |  |
|  |  | Ne_Sca_10 | UniProt | A0A4U5PCC0 | N100 | N214 |
|  |  | Ne_Sca_11 | UniProt | A0A4U5PBK2 |  |  |
|  |  | Ne_Sca_12 | UniProt | A0A4U5NE73 | N81 |  |
|  |  | Ne_Sca_13 | UniProt | A0A4U8UXQ0 |  | N223 |
|  |  | Ne_Sca_14 | UniProt | A0A4U5PBJ6 |  |  |
|  |  | Ne_Sca_15 | UniProt | A0A4U5LTW5 |  |  |
|  |  | Ne_Sca_16 | UniProt | A0A4U5PCH9 | N79 |  |
|  | *Strongyloides papillosus* | Ne_Spa_01 | UniProt | A0A0N5C4A6 | N79 | N254 |
|  |  | Ne_Spa_02 | UniProt | A0A0N5CI52 |  |  |
|  |  | Ne_Spa_03 | UniProt | A0A0N5C763 |  |  |
|  |  | Ne_Spa_04 | UniProt | A0A0N5BCL3 |  |  |
|  |  | Ne_Spa_05 | UniProt | A0A0N5B2H8 |  |  |
|  |  | Ne_Spa_06 | UniProt | A0A0N5CF27 |  |  |
|  |  | Ne_Spa_07 | UniProt | A0A0N5BUD7 |  | N241 |
|  |  | Ne_Spa_08 | UniProt | A0A0N5C8P0 |  |  |
|  |  | Ne_Spa_09 | UniProt | A0A0N5C764 |  |  |
|  |  | Ne_Spa_10 | UniProt | A0A0N5BUL9 |  | N272 |
|  |  | Ne_Spa_11 | UniProt | A0A0N5BPE4 |  |  |
|  |  | Ne_Spa_12 | UniProt | A0A0N5BKZ3 |  |  |
|  |  | Ne_Spa_13 | UniProt | A0A0N5BLB2 |  | N223 |
|  |  | Ne_Spa_14 | UniProt | A0A0N5C1V1 |  |  |
|  |  | Ne_Spa_15 | UniProt | A0A0N5C8M4 | N100 |  |
|  |  | Ne_Spa_16 | UniProt | A0A0N5BI31 |  |  |
|  |  | Ne_Spa_17 | UniProt | A0A0N5BYP9 |  |  |
|  |  | Ne_Spa_18 | UniProt | A0A0N5C8M3 |  |  |
|  |  | Ne_Spa_19 | UniProt | A0A0N5BLJ6 |  |  |
|  |  | Ne_Spa_20 | UniProt | A0A0N5BI66 |  |  |
|  |  | Ne_Spa_21 | UniProt | A0A0N5B7R2 |  |  |
|  |  | Ne_Spa_22 | UniProt | A0A0N5BUM4 |  |  |
|  |  | Ne_Spa_23 | UniProt | A0A0N5B239 |  |  |
|  |  | Ne_Spa_24 | UniProt | A0A0N5B7R1 | N84 |  |
|  | *Parastrongyloides trichosuri* | Ne_Ptr_01 | UniProt | A0A0N4ZZ03 |  |  |
|  |  | Ne_Ptr_02 | UniProt | A0A0N5A0D4 |  |  |
|  |  | Ne_Ptr_03 | UniProt | A0A0N4Z4Q8 |  |  |
|  |  | Ne_Ptr_04 | UniProt | A0A0N4ZP26 |  |  |
|  |  | Ne_Ptr_05 | UniProt | A0A0N4ZJ42 |  |  |
|  |  | Ne_Ptr_06 | UniProt | A0A0N4ZBU3 |  | N241 |
|  |  | Ne_Ptr_07 | UniProt | A0A0N4Z2L1 |  |  |
|  |  | Ne_Ptr_08 | UniProt | A0A0N4ZIL6 |  |  |
|  |  | Ne_Ptr_09 | UniProt | A0A0N5A1F7 | N82 |  |
|  |  | Ne_Ptr_10 | UniProt | A0A0N5A409 |  |  |
|  |  | Ne_Ptr_11 | UniProt | A0A0N4Z539 | N84 | N220 |
|  |  | Ne_Ptr_12 | UniProt | A0A0N4Z7Y1 |  |  |
|  |  | Ne_Ptr_13 | UniProt | A0A0N4ZBL2 | N79 | N254 |
|  |  | Ne_Ptr_14 | UniProt | A0A0N4Z1N1 |  |  |
|  |  | Ne_Ptr_15 | UniProt | A0A0N4ZD13 |  |  |
|  |  | Ne_Ptr_16 | UniProt | A0A0N4Z1N2 |  |  |
|  |  | Ne_Ptr_17 | UniProt | A0A0N4Z3M2 |  |  |
|  |  | Ne_Ptr_18 | UniProt | A0A0N4ZN91 |  |  |
|  |  | Ne_Ptr_19 | UniProt | A0A0N4ZW95 |  | N223 |
|  |  | Ne_Ptr_20 | UniProt | A0A0N4ZRI2 |  |  |
|  |  | Ne_Ptr_21 | UniProt | A0A0N4Z808 |  |  |
|  |  | Ne_Ptr_22 | UniProt | A0A0N4ZN90 | N84 |  |
|  | *Bursaphelenchus xylophilus* | Ne_Bxy_01 | UniProt | A0A1I7RZC3 |  |  |
|  |  | Ne_Bxy_02 | UniProt | A0A1I7SDL3 |  |  |
|  |  | Ne_Bxy_03 | UniProt | A0A1I7SMR4 |  |  |
|  |  | Ne_Bxy_04 | UniProt | A0A1I7S579 |  |  |
|  |  | Ne_Bxy_05 | UniProt | A0A1I7SQ23 | N85 | N244 |
|  |  | Ne_Bxy_06 | UniProt | A0A1I7SAC7 |  |  |
|  |  | Ne_Bxy_07 | UniProt | A0A1I7RJF8 |  |  |
|  |  | Ne_Bxy_08 | UniProt | A0A1I7RRV2 |  |  |
|  |  | Ne_Bxy_09 | UniProt | A0A1I7SAD4 |  |  |
|  |  | Ne_Bxy_10 | UniProt | A0A1I7RSM2 | N84 |  |
|  |  | Ne_Bxy_11 | UniProt | A0A1I7S7M6 |  |  |
|  |  | Ne_Bxy_12 | UniProt | A0A1I7RVC3 |  |  |
|  |  | Ne_Bxy_13 | UniProt | A0A1I7SC16 |  |  |
|  |  | Ne_Bxy_14 | UniProt | A0A1I7SC15 | N100 |  |
|  |  | Ne_Bxy_15 | UniProt | A0A1I7RL21 |  |  |
|  |  | Ne_Bxy_16 | UniProt | A0A1I7STL0 |  |  |
|  |  | Ne_Bxy_17 | UniProt | A0A1I7RUM8 |  |  |
|  |  | Ne_Bxy_18 | UniProt | A0A1I7RL20 | N79 |  |
|  | *Globodera pallida* | Ne_Gpa_01 | UniProt | A0A183CEU7 | N121 |  |
|  |  | Ne_Gpa_02 | UniProt | A0A183C9A7 |  |  |
|  |  | Ne_Gpa_03 | UniProt | A0A183BIG6 |  |  |
|  |  | Ne_Gpa_04 | UniProt | A0A183C5V3 |  | N239 |
|  |  | Ne_Gpa_05 | UniProt | A0A183BYW5 |  |  |
|  |  | Ne_Gpa_06 | UniProt | A0A183CEJ6 |  |  |
|  |  | Ne_Gpa_07 | UniProt | A0A183CFT7 |  | N235 |
|  |  | Ne_Gpa_08 | UniProt | A0A183C0A2 |  |  |
|  |  | Ne_Gpa_09 | UniProt | A0A183CGN3 |  |  |
|  |  | Ne_Gpa_10 | UniProt | A0A183CAY5 |  |  |
|  | *Meloidogyne hapla* | Ne_Mha_01 | UniProt | A0A1I8BJJ9 |  |  |
|  |  | Ne_Mha_02 | UniProt | A0A1I8B043 | N79 | N231 |
|  |  | Ne_Mha_03 | UniProt | A0A1I8BZY8 | N90 | N243 N271 |
|  |  | Ne_Mha_04 | UniProt | A0A1I8BWR9 |  |  |
|  |  | Ne_Mha_05 | UniProt | A0A1I8BGA6 |  |  |
|  |  | Ne_Mha_06 | UniProt | A0A1I8B6I3 |  |  |
|  |  | Ne_Mha_07 | UniProt | A0A1I8BTR7 |  |  |
|  |  | Ne_Mha_08 | UniProt | A0A1I8BV77 |  | N216 |
|  | *Necator americanus* | Ne_Nam_01 | UniProt | W2SJ69 |  |  |
|  |  | Ne_Nam_02 | UniProt | W2STZ3 |  | N358 |
|  |  | Ne_Nam_03 | UniProt | W2TIP6 |  |  |
|  |  | Ne_Nam_04 | UniProt | W2TYF2 |  | N235 |
|  |  | Ne_Nam_05 | UniProt | W2TEZ4 |  |  |
|  |  | Ne_Nam_06 | UniProt | W2T5J4 |  |  |
|  |  | Ne_Nam_07 | UniProt | W2SYE4 | N81 | N244 |
|  |  | Ne_Nam_08 | UniProt | W2T609 |  | N221 |
|  |  | Ne_Nam_09 | UniProt | W2SHV1 |  | N218 N237 |
|  |  | Ne_Nam_10 | UniProt | W2SR35 |  |  |
|  | *Ancylostoma caninum* | Ne_Aca_01 | UniProt | A0A368FCL1 |  | N247 |
|  |  | Ne_Aca_02 | UniProt | A0A368H1X0 |  | N239 |
|  |  | Ne_Aca_03 | UniProt | A0A368GMG3 | N212 |  |
|  |  | Ne_Aca_04 | UniProt | A0A368G5Z5 | N196 |  |
|  |  | Ne_Aca_05 | UniProt | A0A368GSS3 | N103 |  |
|  |  | Ne_Aca_06 | UniProt | A0A368GUK4 | N87 |  |
|  | *Dictyocaulus viviparus* | Ne_Dvi_01 | UniProt | A0A0D8YBA5 |  | N247 |
|  |  | Ne_Dvi_02 | UniProt | A0A0D8XP27 |  |  |
|  |  | Ne_Dvi_03 | UniProt | A0A0D8XE47 |  |  |
|  |  | Ne_Dvi_04 | UniProt | A0A0D8XV81 | N104 |  |
|  |  | Ne_Dvi_05 | UniProt | A0A0D8Y3K3 | N100 |  |
|  |  | Ne_Dvi_06 | UniProt | A0A0D8XVG6 |  | N203 |
|  |  | Ne_Dvi_07 | UniProt | A0A0D8Y4Z3 |  |  |
|  | *Heligmosomoides polygyrus* | Ne_Hpo_01 | UniProt | A0A3P8A072 |  |  |
|  |  | Ne_Hpo_02 | UniProt | A0A3P8DR73 |  |  |
|  |  | Ne_Hpo_03 | UniProt | A0A3P7WSS5 |  |  |
|  |  | Ne_Hpo_04 | UniProt | A0A183GE98 | N92 | N276 |
|  |  | Ne_Hpo_05 | UniProt | A0A3P8AIG2 |  |  |
|  |  | Ne_Hpo_06 | UniProt | A0A183GL76 |  | N239 |
|  |  | Ne_Hpo_07 | UniProt | A0A3P7WVQ0 |  |  |
|  |  | Ne_Hpo_08 | UniProt | A0A3P8BWC6 |  |  |
|  |  | Ne_Hpo_09 | UniProt | A0A3P7ZGT5 |  |  |
|  |  | Ne_Hpo_10 | UniProt | A0A183F297 |  |  |
|  |  | Ne_Hpo_11 | UniProt | A0A183F296 | N81 | N244 |
|  | *Nippostrongylus brasiliensis* | Ne_Nbr_01 | UniProt | A0A0N4YGT7 |  | N382 |
|  |  | Ne_Nbr_02 | UniProt | A0A158QWP9 |  | N362 |
|  |  | Ne_Nbr_03 | UniProt | A0A0N4Y450 | N328 |  |
|  |  | Ne_Nbr_04 | UniProt | A0A0N4XDC6 |  |  |
|  |  | Ne_Nbr_05 | UniProt | A0A158R1W4 |  | N239 |
|  |  | Ne_Nbr_06 | UniProt | A0A0N4YFM3 |  |  |
|  |  | Ne_Nbr_07 | UniProt | A0A0N4YJE4 |  |  |
|  |  | Ne_Nbr_08 | UniProt | A0A158QX74 |  |  |
|  |  | Ne_Nbr_09 | UniProt | A0A0N4XW85 |  | N218 N237 |
|  |  | Ne_Nbr_10 | UniProt | A0A158QZI4 |  |  |
|  |  | Ne_Nbr_11 | UniProt | A0A0N4YE60 |  |  |
|  |  | Ne_Nbr_12 | UniProt | A0A0N4Y9J0 |  |  |
|  |  | Ne_Nbr_13 | UniProt | A0A0N4Y451 | N80 N100 |  |
|  | *Haemonchus contortus* | Ne_Hco_01 | UniProt | A0A6F7PZM9 |  |  |
|  |  | Ne_Hco_02 | UniProt | A0A6F7Q1K4 |  |  |
|  |  | Ne_Hco_03 | UniProt | A0A6F7NY06 |  | N249 N253 |
|  |  | Ne_Hco_04 | UniProt | A0A6F7PJG6 |  | N239 |
|  |  | Ne_Hco_05 | UniProt | A0A6F7PER5 |  | N227 N243 |
|  |  | Ne_Hco_06 | UniProt | A0A6F7Q073 |  |  |
|  |  | Ne_Hco_07 | UniProt | A0A6F7Q2W8 |  |  |
|  |  | Ne_Hco_08 | UniProt | A0A6F7QAF2 |  |  |
|  |  | Ne_Hco_09 | UniProt | A0A6F7PUI7 |  |  |
|  |  | Ne_Hco_10 | UniProt | A0A6F7PQM7 |  |  |
|  |  | Ne_Hco_11 | UniProt | A0A6F7NU31 |  |  |
|  |  | Ne_Hco_12 | UniProt | A0A6F7NSS0 |  |  |
|  |  | Ne_Hco_13 | UniProt | W6NP90 |  | N218 |
|  |  | Ne_Hco_14 | UniProt | A0A6F7Q1Q6 | N87 N93 |  |
|  |  | Ne_Hco_15 | UniProt | A0A6F7NV33 | N81 | N244 |
|  |  | Ne_Hco_16 | UniProt | W6NID6 |  | N221 |
|  |  | Ne_Hco_17 | UniProt | A0A6F7NTH9 |  |  |
|  |  | Ne_Hco_18 | UniProt | A0A6F7NVM9 | N83 |  |

| **Arthropods** | *Thrips palmi* | Ar_Tpa_01 | UniProt | A0A6P8ZST9 |  |  |
| --- | --- | --- | --- | --- | --- | --- |
|  |  | Ar_Tpa_02 | UniProt | A0A6P8Y409 | N72 |  |
|  |  | Ar_Tpa_03 | UniProt | A0A6P8ZV57 |  |  |
|  |  | Ar_Tpa_04 | UniProt | A0A6P8YWU1 |  |  |
|  |  | Ar_Tpa_05 | UniProt | A0A6P8ZRZ8 |  |  |
|  |  | Ar_Tpa_06 | UniProt | A0A6P8ZNF7 |  |  |
|  |  | Ar_Tpa_07 | UniProt | A0A6P8YYE9 |  |  |
|  |  | Ar_Tpa_08 | UniProt | A0A6P8YKL5 |  | N231 |
|  | *Frankliniella occidentalis* | Ar_Foc_01 | UniProt | A0A6J1RZG7 |  |  |
|  |  | Ar_Foc_02 | UniProt | A0A6J1SMB9 |  |  |
|  |  | Ar_Foc_03 | UniProt | A0A6J1RTZ6 |  |  |
|  |  | Ar_Foc_04 | UniProt | A0A6J1SWZ3 |  |  |
|  |  | Ar_Foc_05 | UniProt | A0A6J1S6S1 | N84 |  |
|  |  | Ar_Foc_06 | UniProt | A0A6J1TGB1 |  | N231 |
|  |  | Ar_Foc_07 | UniProt | A0A6J1T3I0 |  |  |
|  | *Aphis craccivora* | Ar_Acr_01 | UniProt | A0A6G0YTT0 |  |  |
|  |  | Ar_Acr_02 | UniProt | A0A6G0ZQU5 |  |  |
|  |  | Ar_Acr_03 | UniProt | A0A6G0ZD86 | N83 |  |
|  |  | Ar_Acr_04 | UniProt | A0A6G0YAS6 |  |  |
|  |  | Ar_Acr_05 | UniProt | A0A6G0YY97 |  |  |
|  |  | Ar_Acr_06 | UniProt | A0A6G0YHN0 |  |  |
|  | *Aphis glycines* | Ar_Agl_01 | UniProt | A0A6G0TLB4 |  |  |
|  |  | Ar_Agl_02 | UniProt | A0A6G0STC3 |  |  |
|  |  | Ar_Agl_03 | UniProt | A0A6G0TLR6 | N83 |  |
|  |  | Ar_Agl_04 | UniProt | A0A6G0TKN3 |  |  |
|  |  | Ar_Agl_05 | UniProt | A0A6G0TL03 |  |  |
|  |  | Ar_Agl_06 | UniProt | A0A6G0TZF1 |  |  |
|  | *Daphnia magna* | Ar_Dma_01 | UniProt | A0A164NX56 |  |  |
|  |  | Ar_Dma_02 | UniProt | A0A0P5IX97 |  |  |
|  |  | Ar_Dma_03 | UniProt | A0A0P5ZCT8 |  |  |
|  |  | Ar_Dma_04 | UniProt | A0A164TF69 |  |  |
|  |  | Ar_Dma_05 | UniProt | A0A0N8DXA0 |  |  |
|  |  | Ar_Dma_06 | UniProt | A0A162SUC4 |  |  |
|  | *Daphnia pulex* | Ar_Dpu_01 | UniProt | E9HBX3 |  |  |
|  |  | Ar_Dpu_02 | UniProt | E9HSG1 |  |  |
|  |  | Ar_Dpu_03 | UniProt | E9GU09 |  |  |
|  |  | Ar_Dpu_04 | UniProt | E9HJ62 |  |  |
|  |  | Ar_Dpu_05 | UniProt | E9HJ63 | N89 |  |
|  |  | Ar_Dpu_06 | UniProt | E9G961 |  |  |
|  |  | Ar_Dpu_07 | UniProt | E9HSG2 |  |  |
|  |  | Ar_Dpu_08 | UniProt | E9HSG3 |  |  |
|  | *Scylla olivacea* | Ar_Sol_01 | UniProt | A0A0P4W675 |  | N217 |
|  |  | Ar_Sol_02 | UniProt | A0A0N7ZD50 |  |  |
|  |  | Ar_Sol_03 | UniProt | A0A0N7ZCP1 |  | N238 N241 |
|  |  | Ar_Sol_04 | UniProt | A0A0P4WHJ5 | N81 |  |
|  |  | Ar_Sol_05 | UniProt | A0A0P4W3U5 |  |  |
|  |  | Ar_Sol_06 | UniProt | A0A0P4WEZ5 |  | N226 |
|  | *Hirondellea gigas* | Ar_Hgi_01 | UniProt | A0A2P2I2N3 | N81 |  |
|  |  | Ar_Hgi_02 | UniProt | A0A6A7FNV7 |  |  |
|  |  | Ar_Hgi_03 | UniProt | A0A2P2I2I6 |  | N238 N241 |
|  |  | Ar_Hgi_04 | UniProt | A0A2P2HZN5 |  | N234 |
|  |  | Ar_Hgi_05 | UniProt | A0A2P2I1N6 |  | N218 N223 |
|  |  | Ar_Hgi_06 | UniProt | A0A6A7FV51 |  |  |
|  |  | Ar_Hgi_07 | UniProt | A0A2P2HW92 |  |  |
|  |  | Ar_Hgi_08 | UniProt | A0A2P2I4A3 |  |  |
|  | *Hyalella azteca* | Ar_Haz_01 | UniProt | A0A6A0GP53 | N98 |  |
|  |  | Ar_Haz_02 | UniProt | A0A6A0HAM8 | N81 |  |
|  |  | Ar_Haz_03 | UniProt | A0A6A0H8A9 | N80 |  |
|  |  | Ar_Haz_04 | UniProt | A0A6A0H700 |  |  |
|  | *Amphibalanus amphitrite* | Ar_Aam_01 | UniProt | A0A6A4VFK7 |  |  |
|  |  | Ar_Aam_02 | UniProt | A0A6A4VDH1 |  |  |
|  |  | Ar_Aam_03 | UniProt | A0A6A4W3Q3 |  |  |
|  |  | Ar_Aam_04 | UniProt | A0A6A4V6H0 |  | N241 |
|  |  | Ar_Aam_05 | UniProt | A0A6A4W2M3 |  |  |
|  |  | Ar_Aam_06 | UniProt | A0A6A4WXZ9 |  |  |
|  |  | Ar_Aam_07 | UniProt | A0A6A4W9V3 |  |  |
|  |  | Ar_Aam_08 | UniProt | A0A6A4W7T0 |  |  |
|  |  | Ar_Aam_09 | UniProt | A0A6A4V6G5 |  |  |
|  |  | Ar_Aam_10 | UniProt | A0A6A4WSI7 |  |  |
|  |  | Ar_Aam_11 | UniProt | A0A6A4WKW5 |  |  |
|  |  | Ar_Aam_12 | UniProt | A0A6A4VJ12 |  | N221 |
|  |  | Ar_Aam_13 | UniProt | A0A6A4WPK5 |  |  |
|  |  | Ar_Aam_14 | UniProt | A0A6A4V3T3 |  |  |
|  | *Tigriopus californicus* | Ar_Tca_01 | UniProt | A0A553P1G4 | N155 |  |
|  |  | Ar_Tca_02 | UniProt | A0A553PJ75 |  |  |
|  |  | Ar_Tca_03 | UniProt | A0A553NFW3 |  |  |
|  |  | Ar_Tca_04 | UniProt | A0A553NEC0 |  |  |
|  |  | Ar_Tca_05 | UniProt | A0A553P1J4 |  |  |
|  |  | Ar_Tca_06 | UniProt | A0A553PKP1 |  |  |
|  |  | Ar_Tca_07 | UniProt | A0A553N8N9 |  |  |
|  |  | Ar_Tca_08 | UniProt | A0A553P1W9 | N55 |  |
|  |  | Ar_Tca_09 | UniProt | A0A553PI24 |  |  |
|  |  | Ar_Tca_10 | UniProt | A0A553PS08 |  |  |
|  |  | Ar_Tca_11 | UniProt | A0A553PHV3 |  |  |
|  |  | Ar_Tca_12 | UniProt | A0A553NNA8 |  |  |
|  |  | Ar_Tca_13 | UniProt | A0A553P4E6 |  |  |
|  |  | Ar_Tca_14 | UniProt | A0A553PBP0 |  |  |
|  |  | Ar_Tca_15 | UniProt | A0A553NE09 | N104 |  |
|  |  | Ar_Tca_16 | UniProt | A0A553PKN4 |  |  |
|  |  | Ar_Tca_17 | UniProt | A0A553NFB7 |  |  |
|  |  | Ar_Tca_18 | UniProt | A0A553PDK2 |  | N246 |
|  |  | Ar_Tca_19 | UniProt | A0A553ND69 |  |  |
|  |  | Ar_Tca_20 | UniProt | A0A553PND8 |  |  |
|  | *Lepeophtheirus salmonis* | Ar_Lsa_01 | UniProt | A0A0K2TDD4 |  |  |
|  |  | Ar_Lsa_02 | UniProt | C1BTQ5 |  |  |
|  |  | Ar_Lsa_03 | UniProt | A0A0K2TMS5 |  |  |
|  |  | Ar_Lsa_04 | UniProt | A0A0K2UKB6 |  |  |
|  |  | Ar_Lsa_05 | UniProt | A0A0K2UW40 | N108 |  |
|  |  | Ar_Lsa_06 | UniProt | A0A0K2TDL8 |  |  |
|  |  | Ar_Lsa_07 | UniProt | A0A0K2T369 | N51 |  |
|  |  | Ar_Lsa_08 | UniProt | A0A0K2T7F6 |  |  |
|  |  | Ar_Lsa_09 | UniProt | A0A0K2T159 |  |  |
|  |  | Ar_Lsa_10 | UniProt | A0A0K2TEQ0 |  |  |
|  |  | Ar_Lsa_11 | UniProt | A0A0K2V323 |  |  |
|  |  | Ar_Lsa_12 | UniProt | C1BSL1 |  |  |
|  |  | Ar_Lsa_13 | UniProt | A0A0K2T1S2 | N93 |  |
|  |  | Ar_Lsa_14 | UniProt | A0A0K2U872 |  |  |
|  |  | Ar_Lsa_15 | UniProt | A0A0K2UID2 | N89 |  |
|  | *Folsomia candida* | Ar_Fca_01 | UniProt | A0A226EIH3 | N450 |  |
|  |  | Ar_Fca_02 | UniProt | A0A226EIV6 | N103 |  |
|  |  | Ar_Fca_03 | UniProt | A0A226E9K9 |  |  |
|  |  | Ar_Fca_04 | UniProt | A0A226F5T4 |  |  |
|  |  | Ar_Fca_05 | UniProt | A0A226ETR7 |  |  |
|  |  | Ar_Fca_06 | UniProt | A0A226EJF9 |  |  |
|  |  | Ar_Fca_07 | UniProt | A0A226EHY9 |  |  |
|  |  | Ar_Fca_08 | UniProt | A0A226EFJ3 |  |  |
|  |  | Ar_Fca_09 | UniProt | A0A226EUL8 |  | N224 |
|  |  | Ar_Fca_10 | UniProt | A0A226F5Z2 |  |  |
|  |  | Ar_Fca_11 | UniProt | A0A226EJ08 |  |  |
|  |  | Ar_Fca_12 | UniProt | A0A226EJI1 |  |  |
|  |  | Ar_Fca_13 | UniProt | A0A226EKK7 |  |  |
|  |  | Ar_Fca_14 | UniProt | A0A226F6A4 | N87 |  |
|  |  | Ar_Fca_15 | UniProt | A0A226EQW4 | N90 |  |
|  | *Orchesella cincta* | Ar_Oci_01 | UniProt | A0A1D2NG12 |  |  |
|  |  | Ar_Oci_02 | UniProt | A0A1D2M8E3 |  |  |
|  |  | Ar_Oci_03 | UniProt | A0A1D2NJ51 |  |  |
|  |  | Ar_Oci_04 | UniProt | A0A1D2NFJ6 |  |  |
|  |  | Ar_Oci_05 | UniProt | A0A1D2MAJ1 |  | N258 |
|  |  | Ar_Oci_06 | UniProt | A0A1D2NGE6 | N78 |  |
|  |  | Ar_Oci_07 | UniProt | A0A1D2MTY0 |  |  |
|  |  | Ar_Oci_08 | UniProt | A0A1D2MD91 |  |  |
|  |  | Ar_Oci_09 | UniProt | A0A1D2M5X8 |  | N240 |
|  |  | Ar_Oci_10 | UniProt | A0A1D2N4L7 |  |  |
|  |  | Ar_Oci_11 | UniProt | A0A1D2M8K9 |  |  |
|  |  | Ar_Oci_12 | UniProt | A0A1D2MTK2 |  |  |
|  | *Strigamia maritima* | Ar_Sma_01 | UniProt | T1J195 |  |  |
|  |  | Ar_Sma_02 | UniProt | T1IHE1 |  | N222 |
|  |  | Ar_Sma_03 | UniProt | T1IXN4 |  | N225 |
|  |  | Ar_Sma_04 | UniProt | T1J6W8 |  | N222 |
|  |  | Ar_Sma_05 | UniProt | T1J585 |  | N220 |
|  | *Blattella germanica* | Ar_Bge_01 | UniProt | A0A2P8YQN8 |  |  |
|  |  | Ar_Bge_02 | UniProt | A0A2P8YAX5 | N73 |  |
|  |  | Ar_Bge_03 | UniProt | A0A2P8YHY4 | N108 | N231 |
|  |  | Ar_Bge_04 | UniProt | A0A2P8YHW8 |  | N238 |
|  |  | Ar_Bge_05 | UniProt | A0A2P8YE60 |  |  |
|  |  | Ar_Bge_06 | UniProt | A0A2P8YZB7 |  |  |
|  |  | Ar_Bge_07 | UniProt | A0A2P8XRI0 |  | N196 |
|  | *Cryptotermes secundus* | Ar_Cse_01 | UniProt | A0A2J7PH97 |  |  |
|  |  | Ar_Cse_02 | UniProt | A0A2J7PSD6 |  | N253 |
|  |  | Ar_Cse_03 | UniProt | A0A2J7PSE5 |  | N231 |
|  |  | Ar_Cse_04 | UniProt | A0A2J7PHB7 | N84 |  |
|  |  | Ar_Cse_05 | UniProt | A0A2J7PH95 | N53 |  |
|  |  | Ar_Cse_06 | UniProt | A0A2J7PHC1 |  |  |
|  |  | Ar_Cse_07 | UniProt | A0A2J7PHA0 |  |  |
|  | *Pediculus humanus subsp. corporis* | Ar_Phu_01 | UniProt | E0VDS4 |  | N232 |
|  |  | Ar_Phu_02 | UniProt | E0VNL8 |  |  |
|  |  | Ar_Phu_03 | UniProt | E0VLP5 |  |  |
|  |  | Ar_Phu_04 | UniProt | E0VDS2 | N86 |  |
|  |  | Ar_Phu_05 | UniProt | E0VDS1 |  |  |
|  | *Clastoptera arizonana* | Ar_Caz_01 | UniProt | A0A1B6CKW9 |  |  |
|  |  | Ar_Caz_02 | UniProt | A0A1B6EBL0 |  |  |
|  |  | Ar_Caz_03 | UniProt | A0A1B6BWP0 |  |  |
|  |  | Ar_Caz_04 | UniProt | A0A1B6D2L4 |  |  |
|  | *Graphocephala atropunctata* | Ar_Gat_01 | UniProt | A0A1B6L2T6 |  |  |
|  |  | Ar_Gat_02 | UniProt | A0A1B6MLB6 |  |  |
|  |  | Ar_Gat_03 | UniProt | A0A1B6LYM0 |  |  |
|  |  | Ar_Gat_04 | UniProt | A0A1B6KPH5 |  | N231 |
|  |  | Ar_Gat_05 | UniProt | A0A1B6KNT5 |  |  |
|  | *Cuerna arida* | Ar_Car_01 | UniProt | A0A1B6GUF3 |  | N231 |
|  |  | Ar_Car_02 | UniProt | A0A1B6GPC7 | N88 |  |
|  |  | Ar_Car_03 | UniProt | A0A1B6G3H6 |  |  |
|  |  | Ar_Car_04 | UniProt | A0A1B6FA42 |  |  |
|  |  | Ar_Car_05 | UniProt | A0A1B6EIJ8 |  |  |
|  |  | Ar_Car_06 | UniProt | A0A1B6GUR0 |  |  |
|  |  | Ar_Car_07 | UniProt | A0A1B6EWA1 |  |  |
|  |  | Ar_Car_08 | UniProt | A0A1B6GPQ2 |  |  |
|  | *Homalodisca liturata* | Ar_Hli_01 | UniProt | A0A1B6HU79 |  |  |
|  |  | Ar_Hli_02 | UniProt | A0A1B6IFW0 |  |  |
|  |  | Ar_Hli_03 | UniProt | A0A1B6IPY0 |  |  |
|  |  | Ar_Hli_04 | UniProt | A0A1B6JAV4 |  | N231 |
|  | *Triatoma infestans* | Ar_Tin_01 | UniProt | A0A023F3Q9 |  |  |
|  |  | Ar_Tin_02 | UniProt | A0A023F5Q4 |  |  |
|  |  | Ar_Tin_03 | UniProt | A0A023F482 |  |  |
|  |  | Ar_Tin_04 | UniProt | A0A023F430 |  |  |
|  | *Apolygus lucorum* | Ar_Alu_01 | UniProt | A0A6A4JQW2 |  |  |
|  |  | Ar_Alu_02 | UniProt | A0A6A4JUI1 | N110 |  |
|  |  | Ar_Alu_03 | UniProt | A0A6A4K089 |  |  |
|  |  | Ar_Alu_04 | UniProt | A0A6A4K500 |  |  |
|  |  | Ar_Alu_05 | UniProt | A0A6A4JS69 |  |  |
|  | *Lygus hesperus* | Ar_Lhe_01 | UniProt | A0A0A9XL95 | N83 |  |
|  |  | Ar_Lhe_02 | UniProt | A0A0A9WAN6 |  |  |
|  |  | Ar_Lhe_03 | UniProt | A0A146LXY8 |  |  |
|  |  | Ar_Lhe_04 | UniProt | A0A0A9VUC1 |  |  |
|  |  | Ar_Lhe_05 | UniProt | A0A146LYX0 |  |  |
|  | *Cinara cedri* | Ar_Cce_01 | UniProt | A0A5E4MG28 |  |  |
|  |  | Ar_Cce_02 | UniProt | A0A5E4M383 |  | N228 |
|  |  | Ar_Cce_03 | UniProt | A0A5E4MFV4 |  |  |
|  |  | Ar_Cce_04 | UniProt | A0A5E4MI47 | N61 |  |
|  |  | Ar_Cce_05 | UniProt | A0A5E4N043 |  |  |
|  |  | Ar_Cce_06 | UniProt | A0A5E4MKN9 |  |  |
|  | *Rhodnius prolixus* | Ar_Rpr_01 | UniProt | T1HJJ5 |  |  |
|  |  | Ar_Rpr_02 | UniProt | T1HVH6 |  |  |
|  |  | Ar_Rpr_03 | UniProt | T1ICT1 |  |  |
|  |  | Ar_Rpr_04 | UniProt | T1HXJ9 |  |  |
|  |  | Ar_Rpr_05 | UniProt | T1I6M9 |  |  |
|  | *Acyrthosiphon pisum* | Ar_Api_01 | UniProt | J9JUV0 |  |  |
|  |  | Ar_Api_02 | UniProt | J9JSD1 |  |  |
|  |  | Ar_Api_03 | UniProt | J9K934 |  |  |
|  |  | Ar_Api_04 | UniProt | J9K6R4 |  |  |
|  |  | Ar_Api_05 | UniProt | J9K6I2 |  |  |
|  |  | Ar_Api_06 | UniProt | J9JWH0 | N83 |  |
|  | *Melanaphis sacchari* | Ar_Msa_01 | UniProt | A0A2H8TIN1 |  |  |
|  |  | Ar_Msa_02 | UniProt | A0A2H8TDG7 |  |  |
|  |  | Ar_Msa_03 | UniProt | A0A2H8TVN8 |  |  |
|  |  | Ar_Msa_04 | UniProt | A0A2H8TSZ7 |  |  |
|  | *Schizaphis graminum* | Ar_Sgr_01 | UniProt | A0A2S2PAU7 |  |  |
|  |  | Ar_Sgr_02 | UniProt | A0A2S2NYF6 |  |  |
|  |  | Ar_Sgr_03 | UniProt | A0A2S2NF27 |  |  |
|  |  | Ar_Sgr_04 | UniProt | A0A2S2NNK0 |  |  |
|  |  | Ar_Sgr_05 | UniProt | A0A2S2P2Y2 |  |  |
|  | *Homarus americanus* | Ar_Ham_01 | UniProt | A0A097KUQ9 | N81 |  |
|  |  | Ar_Ham_02 | UniProt | A0A097KUQ6 |  |  |
|  |  | Ar_Ham_03 | UniProt | A0A097KUR2 |  |  |
|  |  | Ar_Ham_04 | UniProt | A0A097KUP7 |  |  |
|  |  | Ar_Ham_05 | UniProt | A0A097KUP5 |  |  |
|  | *Parasteatoda tepidariorum* | Ar_Pte_01 | UniProt | A0A2L2YN85 |  |  |
|  |  | Ar_Pte_02 | UniProt | A0A2L2YNL8 |  |  |
|  | *Araneus ventricosus* | Ar_Ave_01 | UniProt | A0A4Y2BLW1 |  |  |
|  |  | Ar_Ave_02 | UniProt | A0A4Y2J013 |  |  |
|  |  | Ar_Ave_03 | UniProt | A0A4Y2BJD5 |  |  |
|  |  | Ar_Ave_04 | UniProt | A0A4Y2PP27 |  |  |
|  |  | Ar_Ave_05 | UniProt | A0A4Y2G161 | N82 |  |
|  |  | Ar_Ave_06 | UniProt | A0A4Y2BKB8 |  | N223 |
|  |  | Ar_Ave_07 | UniProt | A0A4Y2BLW9 |  | N226 |
|  |  | Ar_Ave_08 | UniProt | A0A4Y2MVI3 |  | N228 |
|  |  | Ar_Ave_09 | UniProt | A0A4Y2J1R1 |  |  |
|  |  | Ar_Ave_10 | UniProt | A0A4Y2RHT3 |  |  |
|  |  | Ar_Ave_11 | UniProt | A0A4Y2BKZ5 |  |  |
|  |  | Ar_Ave_12 | UniProt | A0A4Y2BJF0 |  |  |
|  |  | Ar_Ave_13 | UniProt | A0A4Y2UAN0 |  | N228 |
|  |  | Ar_Ave_14 | UniProt | A0A4Y2KIL1 |  |  |
|  |  | Ar_Ave_15 | UniProt | A0A4Y2I839 |  |  |
|  |  | Ar_Ave_16 | UniProt | A0A4Y2J0N9 | N83 |  |
|  | *Stegodyphus mimosarum* | Ar_Smi_01 | UniProt | A0A087UFR3 |  |  |
|  |  | Ar_Smi_02 | UniProt | A0A087T834 | N82 |  |
|  |  | Ar_Smi_03 | UniProt | A0A087UUC3 |  |  |
|  |  | Ar_Smi_04 | UniProt | A0A087TIQ6 |  |  |
|  |  | Ar_Smi_05 | UniProt | A0A087UUC5 |  |  |
|  |  | Ar_Smi_06 | UniProt | A0A087TK54 |  |  |
|  |  | Ar_Smi_07 | UniProt | A0A087U192 |  |  |
|  |  | Ar_Smi_08 | UniProt | A0A087USW5 |  |  |
|  |  | Ar_Smi_09 | UniProt | A0A087UUC4 |  |  |
|  | *Aceria tosichella* | Ar_Ato_01 | UniProt | A0A6G1SAZ5 |  |  |
|  |  | Ar_Ato_02 | UniProt | A0A6G1SDC3 | N81 |  |
|  |  | Ar_Ato_03 | UniProt | A0A6G1SHX9 | N94 |  |
|  |  | Ar_Ato_04 | UniProt | A0A6G1SNI0 | N81 |  |
|  |  | Ar_Ato_05 | UniProt | A0A6G1SHY8 | N82 |  |
|  |  | Ar_Ato_06 | UniProt | A0A6G1SEZ4 | N81 |  |
|  |  | Ar_Ato_07 | UniProt | A0A6G1SG39 |  |  |
|  | *Dinothrombium tinctorium* | Ar_Dti_01 | UniProt | A0A3S3P5I6 |  |  |
|  |  | Ar_Dti_02 | UniProt | A0A3S4R7M3 |  |  |
|  |  | Ar_Dti_03 | UniProt | A0A443RBL2 |  |  |
|  |  | Ar_Dti_04 | UniProt | A0A3S3RUJ0 |  |  |
|  |  | Ar_Dti_05 | UniProt | A0A3S3NV37 |  |  |
|  |  | Ar_Dti_06 | UniProt | A0A3S3RSB1 |  |  |
|  | *Leptotrombidium deliense* | Ar_Lde_01 | UniProt | A0A443SF19 | N71 | N230 |
|  |  | Ar_Lde_02 | UniProt | A0A443SWB2 | N99 |  |
|  |  | Ar_Lde_03 | UniProt | A0A443S6K1 |  |  |
|  |  | Ar_Lde_04 | UniProt | A0A443RXP0 |  |  |
|  |  | Ar_Lde_05 | UniProt | A0A443S083 |  |  |
|  |  | Ar_Lde_06 | UniProt | A0A443SGN1 | N83 |  |
|  |  | Ar_Lde_07 | UniProt | A0A443S186 | N83 |  |
|  |  | Ar_Lde_08 | UniProt | A0A443S8U8 |  |  |
|  |  | Ar_Lde_09 | UniProt | A0A443S445 |  |  |
|  |  | Ar_Lde_10 | UniProt | A0A443S2V1 |  |  |
|  |  | Ar_Lde_11 | UniProt | A0A443RY06 | N109 |  |
|  | *Tetranychus urticae* | Ar_Tur_01 | UniProt | T1KCG2 |  |  |
|  |  | Ar_Tur_02 | UniProt | T1KKN2 |  |  |
|  |  | Ar_Tur_03 | UniProt | T1KCG3 |  |  |
|  |  | Ar_Tur_04 | UniProt | T1K2R1 |  | N276 |
|  |  | Ar_Tur_05 | UniProt | T1KEY2 | N118 |  |
|  |  | Ar_Tur_06 | UniProt | T1JXB6 |  |  |
|  |  | Ar_Tur_07 | UniProt | T1KDH8 |  |  |
|  |  | Ar_Tur_08 | UniProt | T1JWY4 |  |  |
|  |  | Ar_Tur_09 | UniProt | T1KKV8 |  |  |
|  |  | Ar_Tur_10 | UniProt | T1KKV7 |  |  |
|  |  | Ar_Tur_11 | UniProt | T1KCG9 |  |  |
|  |  | Ar_Tur_12 | UniProt | T1JY39 |  | N226 |
|  |  | Ar_Tur_13 | UniProt | T1KQB8 |  |  |
|  |  | Ar_Tur_14 | UniProt | T1KQA9 |  |  |
|  |  | Ar_Tur_15 | UniProt | T1L390 | N82 |  |
|  |  | Ar_Tur_16 | UniProt | T1KQD2 |  |  |
|  |  | Ar_Tur_17 | UniProt | T1JVL3 |  |  |
|  | *Dermatophagoides pteronyssinus* | Ar_Dpt_01 | UniProt | A0A6P6XS70 |  |  |
|  |  | Ar_Dpt_02 | UniProt | A0A6P6XMQ2 |  |  |
|  |  | Ar_Dpt_03 | UniProt | A0A6P6XLA4 |  |  |
|  |  | Ar_Dpt_04 | UniProt | A0A6P6XLM4 | N87 |  |
|  |  | Ar_Dpt_05 | UniProt | A0A6P6YEC3 |  |  |
|  |  | Ar_Dpt_06 | UniProt | A0A6P6Y059 |  |  |
|  |  | Ar_Dpt_07 | UniProt | A0A6P6XRT7 | N60 |  |
|  |  | Ar_Dpt_08 | UniProt | A0A6P6XXH3 |  |  |
|  | *Ornithodoros erraticus* | Ar_Oer_01 | UniProt | A0A293M8N1 |  |  |
|  |  | Ar_Oer_02 | UniProt | A0A293LNE0 |  |  |
|  |  | Ar_Oer_03 | UniProt | A0A293LIM4 |  |  |
|  |  | Ar_Oer_04 | UniProt | A0A293LPQ9 | N83 |  |
|  |  | Ar_Oer_05 | UniProt | A0A293MMD4 |  | N199 |
|  | *Amblyomma aureolatum* | Ar_Aau_01 | UniProt | A0A1E1XAB3 | N83 |  |
|  |  | Ar_Aau_02 | UniProt | A0A1E1XA91 |  |  |
|  |  | Ar_Aau_03 | UniProt | A0A1E1X6L2 |  |  |
|  |  | Ar_Aau_04 | UniProt | A0A1E1X7A1 | N84 |  |
|  |  | Ar_Aau_05 | UniProt | A0A1E1XAM5 |  |  |
|  | *Ixodes ricinus* | Ar_Iri_01 | UniProt | A0A131XS32 | N83 |  |
|  |  | Ar_Iri_02 | UniProt | A0A0K8RDF6 | N84 |  |
|  |  | Ar_Iri_03 | UniProt | A0A131XRY4 |  |  |
|  |  | Ar_Iri_04 | UniProt | A0A131XS64 |  |  |
|  |  | Ar_Iri_05 | UniProt | V5HSI1 |  |  |
|  |  | Ar_Iri_06 | UniProt | A0A0K8R574 |  |  |
|  | *Tropilaelaps mercedesae* | Ar_Tme_01 | UniProt | A0A1V9X292 |  |  |
|  |  | Ar_Tme_02 | UniProt | A0A1V9Y2L1 |  |  |
|  |  | Ar_Tme_03 | UniProt | A0A1V9XCM4 |  |  |
|  |  | Ar_Tme_04 | UniProt | A0A1V9XWP2 |  |  |
|  |  | Ar_Tme_05 | UniProt | A0A1V9XKX1 |  |  |
|  |  | Ar_Tme_06 | UniProt | A0A1V9XQS5 |  |  |
|  |  | Ar_Tme_07 | UniProt | A0A1V9Y0N1 | N99 | N216 |
|  |  | Ar_Tme_08 | UniProt | A0A1V9WYG6 |  |  |
|  |  | Ar_Tme_09 | UniProt | A0A1V9XV07 |  |  |
|  | *Rhipicephalus microplus* | Ar_Rmi_01 | UniProt | A0A6M2D420 | N83 |  |
|  |  | Ar_Rmi_02 | UniProt | A0A6M2CIN8 | N84 |  |
|  |  | Ar_Rmi_03 | UniProt | A0A6M2D5Y1 |  |  |
|  |  | Ar_Rmi_04 | UniProt | A0A6G4ZXQ2 |  |  |
|  | *Dendroctonus ponderosae* | Ar_Dpo_01 | UniProt | N6T152 |  |  |
|  |  | Ar_Dpo_02 | UniProt | N6T7Y7 |  |  |
|  |  | Ar_Dpo_03 | UniProt | N6U8L4 |  | N228 |
|  |  | Ar_Dpo_04 | UniProt | N6U651 |  |  |
|  |  | Ar_Dpo_05 | UniProt | J3JTV6 |  | N215 |
|  |  | Ar_Dpo_06 | UniProt | N6TJ28 |  |  |
|  |  | Ar_Dpo_07 | UniProt | U4U1I5 |  |  |
|  |  | Ar_Dpo_08 | UniProt | J3JY90 |  |  |
|  |  | Ar_Dpo_09 | UniProt | N6TC47 |  |  |
|  |  | Ar_Dpo_10 | UniProt | U4U3L4 |  |  |
|  | *Sitophilus oryzae* | Ar_Sor_01 | UniProt | A0A6J2XP14 | N114 |  |
|  |  | Ar_Sor_02 | UniProt | A0A6J2XEW7 |  | N228 |
|  |  | Ar_Sor_03 | UniProt | A0A6J2X1S7 |  |  |
|  |  | Ar_Sor_04 | UniProt | A0A6J2XM62 |  |  |
|  |  | Ar_Sor_05 | UniProt | A0A6J2X1Z6 |  |  |
|  | *Asbolus verrucosus* | Ar_Avr_01 | UniProt | A0A482W4S4 | N77 |  |
|  |  | Ar_Avr_02 | UniProt | A0A482VSR4 |  | N220 N228 |
|  |  | Ar_Avr_03 | UniProt | A0A482VC95 |  |  |
|  |  | Ar_Avr_04 | UniProt | A0A482VLG2 |  |  |
|  | *Tribolium castaneum* | Ar_Tct_01 | UniProt | D6X0D3 |  | N220 N228 |
|  |  | Ar_Tct_02 | UniProt | A0A139WAT1 |  |  |
|  |  | Ar_Tct_03 | UniProt | D6X4I9 | N104 |  |
|  |  | Ar_Tct_04 | UniProt | D6X4I8 |  |  |
|  |  | Ar_Tct_05 | UniProt | D6X4I2 |  |  |
|  |  | Ar_Tct_06 | UniProt | D6WYF3 |  |  |
|  |  | Ar_Tct_07 | UniProt | D6X4J0 |  |  |
|  |  | Ar_Tct_08 | UniProt | D6X4I5 |  | N215 |
|  | *Agrilus planipennis* | Ar_Apl_01 | UniProt | A0A1W4XSA6 |  | N228 |
|  |  | Ar_Apl_02 | UniProt | A0A1W4XQJ8 |  |  |
|  |  | Ar_Apl_03 | UniProt | A0A1W4XPB6 |  |  |
|  |  | Ar_Apl_04 | UniProt | A0A1W4WZU8 |  |  |
|  | *Photinus pyralis* | Ar_Ppy_01 | UniProt | A0A1Y1L4F3 | N105 |  |
|  |  | Ar_Ppy_02 | UniProt | A0A1Y1KBU2 | N84 |  |
|  |  | Ar_Ppy_03 | UniProt | A0A5N4A936 |  |  |
|  |  | Ar_Ppy_04 | UniProt | A0A1Y1M793 |  |  |
|  |  | Ar_Ppy_05 | UniProt | A0A1Y1MWX4 |  |  |
|  |  | Ar_Ppy_06 | UniProt | A0A1Y1NGD2 |  | N215 |
|  | *Ceratitis capitata* | Ar_Cca_01 | UniProt | W8AZ10 |  |  |
|  |  | Ar_Cca_02 | UniProt | W8CE01 |  |  |
|  |  | Ar_Cca_03 | UniProt | W8B2W5 |  |  |
|  |  | Ar_Cca_04 | UniProt | W8B342 |  |  |
|  |  | Ar_Cca_05 | UniProt | W8BD06 | N83 |  |
|  |  | Ar_Cca_06 | UniProt | W8AKP4 |  |  |
|  |  | Ar_Cca_07 | UniProt | W8B620 |  |  |
|  | *Drosophila albomicans* | Ar_Dal_01 | UniProt | A0A6P8Y0G4 |  |  |
|  |  | Ar_Dal_02 | UniProt | A0A6P8WFM3 | N101 |  |
|  |  | Ar_Dal_03 | UniProt | A0A6P8ZFX3 | N80 |  |
|  |  | Ar_Dal_04 | UniProt | A0A6P8Y1Y0 |  |  |
|  |  | Ar_Dal_05 | UniProt | A0A6P8Y660 | N103 |  |
|  |  | Ar_Dal_06 | UniProt | A0A6P8Y014 |  |  |
|  |  | Ar_Dal_07 | UniProt | A0A6P8Y7C9 |  |  |
|  | *Drosophila grimshawi* | Ar_Dgr_01 | UniProt | B4JF44 |  |  |
|  |  | Ar_Dgr_02 | UniProt | B4JMS8 |  |  |
|  |  | Ar_Dgr_03 | UniProt | B4JXM9 |  |  |
|  |  | Ar_Dgr_04 | UniProt | B4JN21 |  |  |
|  |  | Ar_Dgr_05 | UniProt | B4JKB2 | N80 |  |
|  |  | Ar_Dgr_06 | UniProt | B4JMT1 |  |  |
|  |  | Ar_Dgr_07 | UniProt | B4IXW7 | N86 |  |
|  | *Drosophila melanogaster* | Ar_Dme_01 | UniProt | Q9VR82 | N104 |  |
|  |  | Ar_Dme_02 | UniProt | Q9V3W6 | N83 |  |
|  |  | Ar_Dme_03 | UniProt | Q9VWL5 |  |  |
|  |  | Ar_Dme_04 | UniProt | A0A0B4LHT3 |  |  |
|  |  | Ar_Dme_05 | UniProt | Q9VAS7 |  |  |
|  |  | Ar_Dme_06 | UniProt | P33085 |  |  |
|  |  | Ar_Dme_07 | UniProt | Q9VRX6 | N77 |  |
|  |  | Ar_Dme_08 | UniProt | Q9V427 |  |  |
|  |  | Ar_Dme_09 | UniProt | P27716 |  |  |
|  | *Glossina austeni* | Ar_Gau_01 | UniProt | A0A1A9UUN4 |  |  |
|  |  | Ar_Gau_02 | UniProt | A0A1A9VAH4 |  |  |
|  |  | Ar_Gau_03 | UniProt | A0A1A9V3V9 | N93 N116 |  |
|  |  | Ar_Gau_04 | UniProt | A0A1A9UUN2 |  |  |
|  |  | Ar_Gau_05 | UniProt | A0A1A9V3W4 |  |  |
|  | *Musca domestica* | Ar_Mdo_01 | UniProt | T1PCB4 | N83 |  |
|  |  | Ar_Mdo_02 | UniProt | A0A1I8MBQ8 |  |  |
|  |  | Ar_Mdo_03 | UniProt | A0A1I8NDJ6 | N93 |  |
|  |  | Ar_Mdo_04 | UniProt | T1PEY5 |  |  |
|  |  | Ar_Mdo_05 | UniProt | A0A1I8MU37 |  |  |
|  | *Bombyx mori* | Ar_Bmo_01 | UniProt | H9IT73 | N85 |  |
|  |  | Ar_Bmo_02 | UniProt | Q5XLD8 |  | N232 |
|  |  | Ar_Bmo_03 | UniProt | Q6SA04 |  |  |
|  | *Manduca sexta* | Ar_Mse_01 | UniProt | A0A517BE25 | N85 | N235 |
|  |  | Ar_Mse_02 | UniProt | A0A517BE21 | N85 |  |
|  |  | Ar_Mse_03 | UniProt | A0A517BE34 |  |  |
|  |  | Ar_Mse_04 | UniProt | A0A517BE20 |  |  |
|  |  | Ar_Mse_05 | UniProt | A0A517BE40 |  |  |
|  |  | Ar_Mse_06 | UniProt | A0A517BE30 |  |  |
|  | *Heliothis virescens* | Ar_Hvi_01 | UniProt | A0A2A4K0X0 |  |  |
|  |  | Ar_Hvi_02 | UniProt | A0A2A4K6A3 | N82 |  |
|  |  | Ar_Hvi_03 | UniProt | A0A2A4K6C4 |  |  |
|  |  | Ar_Hvi_04 | UniProt | Q6GVH1 |  |  |
|  |  | Ar_Hvi_05 | UniProt | A0A2A4K2D6 | N86 |  |
|  | *Danaus plexippus plexippus* | Ar_Dpl_01 | UniProt | A0A212FCE9 | N85 |  |
|  |  | Ar_Dpl_02 | UniProt | A0A212F4N9 |  |  |
|  |  | Ar_Dpl_03 | UniProt | A0A212EW68 |  |  |
|  |  | Ar_Dpl_04 | UniProt | A0A212EYK5 |  |  |
|  | *Papilio machaon* | Ar_Pma_01 | UniProt | A0A0N1I9S5 |  |  |
|  |  | Ar_Pma_02 | UniProt | A0A194QW93 |  |  |
|  |  | Ar_Pma_03 | UniProt | A0A0N1IHM3 |  |  |
|  |  | Ar_Pma_04 | UniProt | A0A0N1IGD1 |  |  |
|  |  | Ar_Pma_05 | UniProt | A0A0N0PCR1 |  |  |
|  | *Galleria mellonella* | Ar_Gme_01 | UniProt | A0A6J1WCG0 | N62 |  |
|  |  | Ar_Gme_02 | UniProt | A0A6J1WMU7 |  |  |
|  |  | Ar_Gme_03 | UniProt | A0A6J1X3M4 |  |  |
|  |  | Ar_Gme_04 | UniProt | A0A6J1X371 |  |  |
|  | *Apis mellifera* | Ar_Ame_01 | UniProt | A0A088A2D6 | N237 |  |
|  |  | Ar_Ame_02 | UniProt | A0A088A2D0 | N92 |  |
|  |  | Ar_Ame_03 | UniProt | A0A088AID2 |  |  |
|  | *Bombus bifarius* | Ar_Bbi_01 | UniProt | A0A6P8LE89 |  |  |
|  |  | Ar_Bbi_02 | UniProt | A0A6P8LET5 |  |  |
|  |  | Ar_Bbi_03 | UniProt | A0A6P8LT46 | N92 |  |
|  |  | Ar_Bbi_04 | UniProt | A0A6P8MF07 |  |  |
|  |  | Ar_Bbi_05 | UniProt | A0A6P8M4T9 |  |  |
|  |  | Ar_Bbi_06 | UniProt | A0A6P8ND58 |  |  |
|  | *Lasius niger* | Ar_Lni_01 | UniProt | A0A0J7KSE9 |  |  |
|  |  | Ar_Lni_02 | UniProt | A0A0J7KZE2 | N57 N82 |  |
|  |  | Ar_Lni_03 | UniProt | A0A0J7LA69 |  |  |
|  |  | Ar_Lni_04 | UniProt | A0A0J7KFC1 |  |  |
|  | *Atta cephalotes* | Ar_Ace_01 | UniProt | A0A158NQK4 |  |  |
|  |  | Ar_Ace_02 | UniProt | A0A158NU57 |  |  |
|  |  | Ar_Ace_03 | UniProt | A0A158NQK3 |  |  |
|  |  | Ar_Ace_04 | UniProt | A0A158NPV6 |  |  |
|  |  | Ar_Ace_05 | UniProt | A0A158NQK6 |  |  |
|  |  | Ar_Ace_06 | UniProt | A0A158NQK5 | N91 |  |
|  | *Dinoponera quadriceps* | Ar_Dqu_01 | UniProt | A0A6P3XJ21 |  |  |
|  |  | Ar_Dqu_02 | UniProt | A0A6P3XHS1 | N92 |  |
|  |  | Ar_Dqu_03 | UniProt | A0A6P3XJ24 |  |  |
|  |  | Ar_Dqu_04 | UniProt | A0A6P3XFQ9 |  |  |
|  |  | Ar_Dqu_05 | UniProt | A0A6P3XJL3 |  |  |
|  |  | Ar_Dqu_06 | UniProt | A0A6P3X6E4 |  |  |
|  | *Ooceraea biroi* | Ar_Obi_01 | UniProt | A0A026WUY9 |  |  |
|  |  | Ar_Obi_02 | UniProt | A0A026WWJ5 |  |  |
|  |  | Ar_Obi_03 | UniProt | A0A3L8E3W4 | N88 |  |
|  |  | Ar_Obi_04 | UniProt | A0A026X0B1 |  |  |
|  |  | Ar_Obi_05 | UniProt | A0A026WVZ4 |  |  |
|  |  | Ar_Obi_06 | UniProt | A0A026WXJ9 |  |  |
|  | *Nasonia vitripennis* | Ar_Nvi_01 | UniProt | K7J6Q6 |  |  |
|  |  | Ar_Nvi_02 | UniProt | K7J6Q8 |  | N216 |
|  |  | Ar_Nvi_03 | UniProt | K7J6Q9 | N89 |  |
|  |  | Ar_Nvi_04 | UniProt | K7J0A9 |  |  |
|  | *Fopius arisanus* | Ar_Far_01 | UniProt | A0A0C9QE81 |  |  |
|  |  | Ar_Far_02 | UniProt | A0A0C9R0I7 | N91 |  |
|  |  | Ar_Far_03 | UniProt | A0A0C9QJQ2 |  |  |
|  |  | Ar_Far_04 | UniProt | A0A0C9QHU7 |  |  |
|  |  | Ar_Far_05 | UniProt | A0A0C9QAE6 |  |  |
|  |  | Ar_Far_06 | UniProt | A0A0C9PHI7 |  |  |
|  | *Neodiprion lecontei* | Ar_Nle_01 | UniProt | A0A6J0BWI9 |  |  |
|  |  | Ar_Nle_02 | UniProt | A0A6J0C563 |  |  |
|  |  | Ar_Nle_03 | UniProt | A0A6J0BVT8 | N88 |  |
|  |  | Ar_Nle_04 | UniProt | A0A6J0BXN6 |  |  |
|  |  | Ar_Nle_05 | UniProt | A0A6J0B4W1 |  |  |
|  |  | Ar_Nle_06 | UniProt | A0A6J0BXT1 |  |  |
|  |  | Ar_Nle_07 | UniProt | A0A6J0BUN7 |  |  |
|  | *Cancer borealis* | Ar_Cbo_01 | NCBI | AFN25964.1 |  |  |
|  |  | Ar_Cbo_02 | NCBI | AFN25965.1 |  |  |
|  |  | Ar_Cbo_03 | NCBI | AFN25966.1 |  |  |
|  |  | Ar_Cbo_04 | NCBI | AID07491.1 |  |  |
|  |  | Ar_Cbo_05 | NCBI | AIJ10714.1 |  |  |
|  |  | Ar_Cbo_06 | NCBI | AIJ10714.1 |  |  |

| **Xenacoelomorphs** | *Hofstenia miamia* | Xe_Hmi_01 | NCBI TSA | GFSA01016590.1 |  |  |
| --- | --- | --- | --- | --- | --- | --- |
|  |  | Xe_Hmi_02 | NCBI TSA | GFSA01018083.1 |  |  |
|  |  | Xe_Hmi_03 | NCBI TSA | GFSA01025217.1 | N67 |  |
|  |  | Xe_Hmi_04 | NCBI TSA | GFSA01028240.1 |  |  |
|  |  | Xe_Hmi_05 | NCBI TSA | GFSA01035590.1 |  |  |
|  |  | Xe_Hmi_06 | NCBI TSA | GFSA01000361.1 | N51 |  |
|  |  | Xe_Hmi_07 | NCBI TSA | GFSA01040070.1 |  |  |
|  |  | Xe_Hmi_08 | NCBI TSA | GFSA01043372.1 |  |  |
|  |  | Xe_Hmi_09 | NCBI TSA | GFSA01048585.1 | N70 |  |
|  |  | Xe_Hmi_10 | NCBI TSA | GFSA01048994.1 |  |  |
|  |  | Xe_Hmi_11 | NCBI TSA | GFSA01049854.1 |  |  |
|  |  | Xe_Hmi_12 | NCBI TSA | GFSA01052710.1 |  |  |
|  |  | Xe_Hmi_13 | NCBI TSA | GFSA01055299.1 |  |  |
|  |  | Xe_Hmi_14 | NCBI TSA | GFSA01056382.1 |  | N225 |
|  |  | Xe_Hmi_15 | NCBI TSA | GFSA01057723.1 |  |  |
|  |  | Xe_Hmi_16 | NCBI TSA | GFSA01066405.1 | N69 |  |
|  |  | Xe_Hmi_17 | NCBI TSA | GFSA01066474.1 | N73 | N227 N268 |
|  |  | Xe_Hmi_18 | NCBI TSA | GFSA01120359.1 |  | N225 |
|  |  | Xe_Hmi_19 | NCBI TSA | GFSA01120558.1 |  |  |
|  |  | Xe_Hmi_20 | NCBI TSA | GFSA01010893.1 |  |  |
|  | *Isodiametra pulchra* | Xe_Ipu_01 | NCBI TSA | GGBV01013673.1 |  |  |
|  |  | Xe_Ipu_02 | NCBI TSA | GGBV01079800.1 | N51 |  |
|  |  | Xe_Ipu_03 | NCBI TSA | GGBV01097887.1 | N53 |  |
|  |  | Xe_Ipu_04 | NCBI TSA | GGBV01097903.1 |  | N248 |
|  |  | Xe_Ipu_05 | NCBI TSA | GGBV01110131.1 |  |  |
|  |  | Xe_Ipu_06 | NCBI TSA | GGBV01110487.1 |  | N236 |
|  |  | Xe_Ipu_07 | NCBI TSA | GGBV01111987.1 |  |  |
|  |  | Xe_Ipu_08 | NCBI TSA | GGBV01115553.1 |  | N236 |
|  |  | Xe_Ipu_09 | NCBI TSA | GGBV01122036.1 |  |  |
|  |  | Xe_Ipu_10 | NCBI TSA | GGBV01134023.1 |  |  |
|  |  | Xe_Ipu_11 | NCBI TSA | GGBV01139128.1 |  |  |
|  |  | Xe_Ipu_12 | NCBI TSA | GGBV01158397.1 |  |  |
|  |  | Xe_Ipu_13 | NCBI TSA | GGBV01164032.1 |  |  |
|  | *Symsagittifera roscoffensis* | Xe_Sro_01 | NCBI TSA | GFRZ01119853.1 | N59 |  |
|  |  | Xe_Sro_02 | NCBI TSA | GFRZ01138313.1 |  |  |
|  |  | Xe_Sro_03 | NCBI TSA | GFRZ01150821.1 |  |  |
|  |  | Xe_Sro_04 | NCBI TSA | GFRZ01156175.1 |  |  |
|  |  | Xe_Sro_05 | NCBI TSA | GFRZ01158749.1 |  |  |
|  |  | Xe_Sro_06 | NCBI TSA | GFRZ01161908.1 | N51 |  |
|  |  | Xe_Sro_07 | NCBI TSA | GFRZ01184394.1 |  |  |
|  |  | Xe_Sro_08 | NCBI TSA | GFRZ01186732.1 |  |  |
|  |  | Xe_Sro_09 | NCBI TSA | GFRZ01196287.1 |  |  |
|  |  | Xe_Sro_10 | NCBI TSA | GFRZ01206159.1 |  |  |
|  |  | Xe_Sro_11 | NCBI TSA | GFRZ01214008.1 |  |  |
|  |  | Xe_Sro_12 | NCBI TSA | GFRZ01244963.1 |  | N238 |
|  |  | Xe_Sro_13 | NCBI TSA | GFRZ01259699.1 |  |  |
|  |  | Xe_Sro_14 | NCBI TSA | GFRZ01272870.1 |  |  |
|  | *Xenoturbella bocki* | Xe_Xbo_01 | NCBI TSA | GGMI01080781.1 |  |  |
|  |  | Xe_Xbo_02 | NCBI TSA | GGMI01174086.1 |  |  |

| **Echinoderms** | *Apostichopus japonicus* | Ec_Aja_01 | NCBI TSA | GHCH01013653.1 |  |  |
| --- | --- | --- | --- | --- | --- | --- |
|  |  |  | NCBI TSA | GHCH01047549.1 |  |  |
|  | *Arbacia punctulata* | Ec_Apu_01 | NCBI TSA | GECD01051584.1 |  |  |
|  |  |  | NCBI TSA | GECD01003551.1 |  |  |
|  |  |  | NCBI TSA | GECD01013000.1 |  |  |
|  |  |  | NCBI TSA | GECD01070990.1 |  |  |
|  |  |  | NCBI TSA | GECD01026667.1 |  |  |
|  | *Asterias rubens* | Ec_Aru_01 | NCBI TSA | GHKZ01096691.1 | N53 |  |
|  |  |  | NCBI TSA | GHKZ01082365.1 |  |  |
|  | *Paracentrotus lividus* | Ec_Pli_01 | NCBI TSA | GIIR01058804.1 |  |  |
