# Supplementary material for: Connexins evolved after early chordates lost innexin diversity
Source: eLife. 2022 Jan 19;11:e74422. doi: 10.7554/eLife.74422 (PMC8769644; doi:10.7554/eLife.74422)
Supplement: Figure 1—source data 3. — The identified NGSs have a high potential score (see Materials and methods) and the amino acid distribution around the N-glycosylation motif shows no indications for an unoccupancy of the predicted NGSs (Petrescu, 2003). [file elife-74422-fig1-data3.zip › Figure 1source data 3.docx]

**Figure 1–source data 3. Extracellular N-glycosylation sites (NGS) in non-chordate innexins with confirmed gap junction function.** The identified NGS have a high potential score (see Materials and Methods) and the amino acid distribution around the N-glycosylation motif shows no indications for an unoccupancy of the predicted NGSs (Petrescu, 2003)

| **Species** | **Innexin**  **ID** | **Innexin**  **name** | **Accession**  **ID** | **N-glycosylation site (NGS)** | | | | **Experimental evidence for gap junction function** |
| --- | --- | --- | --- | --- | --- | --- | --- | --- |
|  |  |  |  | **Position**  **EL1 EL2** | | **Amino acids around the NGS** | **Potenital score** |  |
|  |  |  |  |  |  |  |  |  |
| *Hydra vulgaris* | Cn_Hvu_11 | Innexin-2 | A0A5B8IE58 | N60 |  | IPPGSNLSQDFVH | 0.7254 | (Takaku et al., 2014) |

| *Hirudo verbana* | An_Hve_02 | Innexin-6 | H9C4Q4 | N78 N93 |  | SICWVNGTYYVPF  YLPLPNQSRTAIL | 0.6874  0.7212 | (Firme et al., 2012) |
| --- | --- | --- | --- | --- | --- | --- | --- | --- |

| *Caenorhabditis elegans* | Ne_Cel_01 | Innexin-10 | Q22549 |  | 239 | LVDLLNGTTWEQS | 0.5980 | (Liu et al., 2013) |
| --- | --- | --- | --- | --- | --- | --- | --- | --- |
|  | Ne_Cel_10 | Innexin-12 | O01634 | 99 |  | SEDKQNTTSLKQT | 0.5006 | (Kovacevic et al., 2013) |
|  | Ne_Cel_12 | Innexin unc-9 | O01393 |  | 223 | NTFLGNRSKWYGL | 0.6573 | (Starich et al., 2009) |

| *Drosophila melanogaster* | Ar_Dme_01 | Innexin-6 | Q9VR82 | 104 |  | AAETFNVSSLRAL | 0.6536 | (Wu et al., 2011) |
| --- | --- | --- | --- | --- | --- | --- | --- | --- |
|  | Ar_Dme_02 | Innexin-7 | Q9V3W6 | 83 |  | VVRDQNQTAYRPG | 0.5032 |  |

**Supplementary References**

Firme, C. P., Natan, R. G., Yazdani, N., Macagno, E. R., & Baker, M. W. (2012). Ectopic expression of select innexins in individual central neurons couples them to pre-existing neuronal or glial networks that express the same innexin. *Journal of Neuroscience*, *32*(41), 14265–14270. doi: 10.1523/JNEUROSCI.2693-12.2012

Kovacevic, I., Orozco, J. M., & Cram, E. J. (2013). Filamin and phospholipase C-ε are required for calcium signaling in the Caenorhabditis elegans spermatheca. *PLoS Genetics*, *9*(5), e1003510. doi: 10.1371/journal.pgen.1003510

Liu, P., Chen, B., Altun, Z. F., Gross, M. J., Shan, A., Schuman, B., Hall, D. H., & Wang, Z.-W. (2013). Six innexins contribute to electrical coupling of C. elegans body-wall muscle. *PLoS ONE*, *8*(10), e76877. doi: 10.1371/journal.pone.0076877

Petrescu, A.-J. (2003). Statistical analysis of the protein environment of N-glycosylation sites: implications for occupancy, structure, and folding. *Glycobiology*, *14*(2), 103–114. doi: 10.1093/glycob/cwh008

Starich, T. A., Xu, J., Skerrett, I. M., Nicholson, B. J., & Shaw, J. E. (2009). Interactions between innexins UNC-7 and UNC-9 mediate electrical synapse specificity in the Caenorhabditis elegans locomotory nervous system. *Neural Development*, *4*(1), 16. doi: 10.1186/1749-8104-4-16

Takaku, Y., Hwang, J. S., Wolf, A., Böttger, A., Shimizu, H., David, C. N., & Gojobori, T. (2014). Innexin gap junctions in nerve cells coordinate spontaneous contractile behavior in Hydra polyps. *Scientific Reports*, *4*(1), 3573. doi: 10.1038/srep03573

Wu, C.-L., Shih, M.-F. M., Lai, J. S.-Y., Yang, H.-T., Turner, G. C., Chen, L., & Chiang, A.-S. (2011). Heterotypic gap junctions between two neurons in the Drosophila brain are critical for memory. *Current Biology*, *21*(10), 848–854. doi: 10.1016/j.cub.2011.02.041
