# Supplementary material for: Connexins evolved after early chordates lost innexin diversity
Source: eLife. 2022 Jan 19;11:e74422. doi: 10.7554/eLife.74422 (PMC8769644; doi:10.7554/eLife.74422)
Supplement: Figure 2—source data 1. [file elife-74422-fig2-data1.zip › Figure 2source data 1.docx]

**Figure 2–source data 1. A list of the identified N-glycosylation sites (NGS) in the extracellular loops of innexins in chordate species.**

| **Taxonomic rank** | **Species** | **Innexin**  **ID** | **Database** | **Accession**  **ID** | **Predicted NGS** | | |
| --- | --- | --- | --- | --- | --- | --- | --- |
|  |  |  |  |  | **EL1** | **EL2** | |
|  |  |  |  |  |  | |  |
| **Lancelets** | *Branchiostoma belcheri* | Ce_Bbe_01 | LanceletDB | Bb_087290F |  | N146 | |
|  | *Branchiostoma floridae* | Ce_Bfl_01 | LanceletDB | Bf_124577 |  | N142 | |
|  | *Branchiostoma lanceolatum* | Ce_Bla_01 | NCBI | FLLO01000068.1 |  | N146 | |
|  | *Asymmetron lucayanum* | Ce_Alu_01 | NCBI | LZCU01211352.1 |  | N142 | |

| **Tunicates** | *Ciona intestinalis* | Tu_Cin_01 | NCBI | XP_026690045 | N65 N107 |  |
| --- | --- | --- | --- | --- | --- | --- |
|  |  | Tu_Cin_02 | NCBI | XP_026690071 | N67 |  |
|  | *Ciona robusta* | Tu_Cro_01 | ANISEED | KH.XM_009860129.2 | N65 N107 |  |
|  |  | Tu_Cro_02 | ANISEED | KH.C4.6.v1.A.ND1-1 | N70 |  |
|  | *Botrylloides leachii* | Tu_Ble_01 | ANISEED | Boleac.CG.SB_v3.S311.g07471.01.t | N90 |  |
|  | *Halocynthia aurantium* | Tu_Hau_01 | ANISEED | Haaura.CG.MTP2014.S63.g01470.01.t | N55 N62 N86 | N454 |
|  | *Halocynthia roretzi* | Tu_Hro_01 | ANISEED | Harore.CG.MTP2014.S25.g05288.01.t | N55 N62 | N457 |
|  | *Molgula oculata* | Tu_Moc_01 | ANISEED | Mooccu.CG.ELv1_2.S726405.g47989.01.t | N66 N84 |  |
|  | *Phallusia mammillata* | Tu_Pma_01 | NCBI | CAB3262992.1 | N87 |  |
|  | *Styela clava* | Tu_Scl_01 | NCBI | XP_039249658.1 | N56 N64 N84 |  |
|  |  | Tu_Scl_02 | NCBI | XP_039249657.1 | N56 N64 |  |
|  |  | Tu_Scl_03 | NCBI | XP_039249659.1 | N120 |  |

| **Lampreys** | *Petromyzon marinus* | La_Pma_01 | NCBI | XP_032833829.1 |  | N252 |
| --- | --- | --- | --- | --- | --- | --- |
|  | *Lethenteron reissneri* | La_Lre_01 | NCBI | CM027349.1 | N71 | N213 |
|  | *Lampetra richardsoni* | La_Lri_01 | SIMRBASE | LPT_00014074-RA | N71 | N248 |
|  | *Entosphenus tridentatus* | La_Etr_01 | NCBI | CM027349.1 |  | N249 |
|  |  |  |  | JAAVTP020010032.1 |  |  |
|  |  |  |  | JAAVTP020004907.1 |  |  |

| **Cartilaginous fish** | *Callorhinchus milii* | Cf_Cmi_P1 | NCBI | Pannexin1 | XP_007900295.1 |  | N251 |
| --- | --- | --- | --- | --- | --- | --- | --- |
|  | *Amblyraja radiata* | Cf_Ara_P1 | NCBI |  | XP_032878304.1 |  | N252 |
|  | *Scyliorhinus canicula* | Cf_Sca_P1 | NCBI |  | XP_038674454.1 |  | N252 |
|  | *Chiloscyllium punctatum* | Cf_Cpu_P1 | NCBI |  | GCC22356.1 |  | N252 |
|  | *Callorhinchus milii* | Cf_Cmi_P2 | NCBI | Pannexin2 | XP_007889224.1 | N87 |  |
|  | *Amblyraja radiata* | Cf_Ara_P2 | NCBI |  | XP_032895673.1 | N86 |  |
|  | *Scyliorhinus canicula* | Cf_Sca_P2 | NCBI |  | XP_038667371.1 | N87 |  |
|  | *Chiloscyllium punctatum* | Cf_Cpu_P2 | NCBI |  | GCC35141.1 | N86 |  |
|  | *Callorhinchus milii* | Cf_Cmi_P3 | NCBI | Pannexin3 | XP_007894201.1 | N71 |  |
|  | *Amblyraja radiata* | Cf_Ara_P3 | NCBI |  | XP_032905581.1 | N71 |  |
|  | *Scyliorhinus canicula* | Cf_Sca_P3 | NCBI |  | XP_038635033.1 | N71 |  |
|  | *Chiloscyllium punctatum* | Cf_Cpu_P3 | NCBI |  | GCC36129.1 | N71 |  |

| **Bony fish** | *Danio rerio* | Bf_Dre_P1a | UniProt | Pannexin1a | Q7ZUN0 |  | N246 |
| --- | --- | --- | --- | --- | --- | --- | --- |
|  | *Sinocyclocheilus anshuiensis* | Bf_San_P1a | UniProt |  | A0A671PKT2 |  | N246 |
|  | *Sinocyclocheilus rhinocerous* | Bf_Srh_P1a | UniProt |  | A0A673GZZ0 |  | N246 |
|  | *Danionella translucida* | Bf_Dtr_P1a | UniProt |  | A0A553QQ25 |  | N245 |
|  | *Triplophysa tibetana* | Bf_Tti_P1a | UniProt |  | A0A5A9MZG0 |  | N244 |
|  | *Pygocentrus nattereri* | Bf_Pna_P1a | UniProt |  | A0A3B4E828 | N72 | N240 |
|  | *Electrophorus electricus* | Bf_Eel_P1a | UniProt |  | A0A4W4EVJ7 | N72 | N240 |
|  | *Astyanax mexicanus* | Bf_Ame_P1a | UniProt |  | A0A3B1IL67 |  | N240 |
|  | *Pangasianodon hypophthalmus* | Bf_Phy_P1a | UniProt |  | A0A5N5KS50 | N72 | N240 |
|  | *Ictalurus punctatus* | Bf_Ipu_P1a | UniProt |  | W5UJ73 | N72 | N237 |
|  | *Chanos chanos* | Bf_Cch_P1a | UniProt |  | A0A6J2VSH4 | N72 | N247 |
|  | *Bagarius yarrelli* | Bf_Bya_P1a | UniProt |  | A0A556TWG5 | N72 | N240 |
|  | *Myripristis murdjan* | Bf_Mmu_P1a | UniProt |  | A0A667ZWQ6 | N71 | N242 |
|  | *Salmo salar* | Bf_Ssa_P1a | UniProt |  | A0A1S3T0A5 | N71 | N235 |
|  | *Esox lucius* | Bf_Elu_P1a | UniProt |  | A0A3P9API0 | N71 | N235 |
|  | *Hucho hucho* | Bf_Hhu_P1a | UniProt |  | A0A4W5QEZ1 | N71 | N235 |
|  | *Coregonus sp. 'balchen'* | Bf_Cba_P1a | UniProt |  | A0A6F9CHI4 | N71 | N235 |
|  | *Salmo trutta* | Bf_Str_P1a | UniProt |  | A0A673X509 | N35 | N199 |
|  | *Seriola dumerili* | Bf_Sdu_P1a | UniProt |  | A0A3B4VCK6 | N71 | N241 |
|  | *Seriola lalandi dorsalis* | Bf_Sdo_P1a | UniProt |  | A0A3B4XGA6 | N71 | N241 |
|  | *Perca flavescens* | Bf_Pfl_P1a | UniProt |  | A0A484DK94 | N71 |  |
|  | *Scophthalmus maximus* | Bf_Sma_P1a | UniProt |  | A0A6A4SYD9 | N90 | N258 |
|  | *Gasterosteus aculeatus* | Bf_Gac_P1a | UniProt |  | G3PIQ8 | N71 |  |
|  | *Cottoperca gobio* | Bf_Cgo_P1a | UniProt |  | A0A6J2R114 | N71 |  |
|  | *Lates calcarifer* | Bf_Lca_P1a | UniProt |  | A0A4W6FFV1 | N71 |  |
|  | *Stegastes partitus* | Bf_Spa_P1a | UniProt |  | A0A3B5B636 | N71 |  |
|  | *Anabas testudineus* | Bf_Ate_P1a | UniProt |  | A0A3Q1HD43 | N71 |  |
|  | *Hippocampus comes* | Bf_Hco_P1a | UniProt |  | A0A3Q2YJL1 | N71 | N233 |
|  | *Echeneis naucrates* | Bf_Ena_P1a | UniProt |  | A0A665TAG8 | N71 |  |
|  | *Oreochromis aureus* | Bf_Oau_P1a | UniProt |  | A0A668W2K3 | N71 |  |
|  | *Oryzias latipes* | Bf_Ola_P1a | UniProt |  | H2L9V9 | N71 |  |
|  | *Perca fluviatilis* | Bf_Pfu_P1a | UniProt |  | A0A6A5FP32 | N71 |  |
|  | *Larimichthys crocea* | Bf_Lcr_P1a | UniProt |  | A0A6G0JBV3 | N71 | N238 |
|  | *Acanthochromis polyacanthus* | Bf_Apo_P1a | UniProt |  | A0A3Q1GSX1 |  | N241 |
|  | *Neolamprologus brichardi* | Bf_Nbr_P1a | UniProt |  | A0A3Q4MW06 | N71 |  |
|  | *Sphaeramia orbicularis* | Bf_Sor_P1a | UniProt |  | A0A673C402 | N60 | N201 |
|  | *Oreochromis niloticus* | Bf_Oni_P1a | UniProt |  | I3JLQ0 | N71 |  |
|  | *Astatotilapia calliptera* | Bf_Aca_P1a | UniProt |  | A0A3P8Q7Q0 | N71 |  |
|  | *Maylandia zebra* | Bf_Mze_P1a | UniProt |  | A0A3P9DFE6 | N71 |  |
|  | *Pundamilia nyererei* | Bf_Pny_P1a | UniProt |  | A0A3B4H620 | N71 |  |
|  | *Haplochromis burtoni* | Bf_Hbu_P1a | UniProt |  | A0A3Q2WJB0 | N71 |  |
|  | *Periophthalmus magnuspinnatus* | Bf_Pma_P1a | UniProt |  | A0A3B4BBG4 | N68 |  |
|  | *Oryzias melastigma* | Bf_Ome_P1a | UniProt |  | A0A3B3B5N4 | N71 | N222 |
|  | *Amphilophus citrinellus* | Bf_Aci_P1a | UniProt |  | A0A3Q0SFT1 | N71 |  |
|  | *Sparus aurata* | Bf_Sau_P1a | UniProt |  | A0A671UA15 | N71 |  |
|  | *Amphiprion ocellaris* | Bf_Aoc_P1a | UniProt |  | A0A3Q1CAG4 | N71 |  |
|  | *Channa argus* | Bf_Car_P1a | UniProt |  | A0A6G1PT53 | N71 |  |
|  | *Austrofundulus limnaeus* | Bf_Ali_P1a | UniProt |  | A0A2I4BUE5 | N71 | N233 |
|  | *Fundulus heteroclitus* | Bf_Fhe_P1a | UniProt |  | A0A3Q2PYY3 | N71 | N233 |
|  | *Amphiprion percula* | Bf_Ape_P1a | UniProt |  | A0A3P8RKM6 | N71 |  |
|  | *Kryptolebias marmoratus* | Bf_Kma_P1a | UniProt |  | A0A3Q3BFU5 | N71 | N233 |
|  | *Oryzias javanicus* | Bf_Oja_P1a | UniProt |  | A0A437CSD2 | N71 |  |
|  | *Labrus bergylta* | Bf_Lbe_P1a | UniProt |  | A0A3Q3EYP6 | N71 |  |
|  | *Poecilia mexicana* | Bf_Pme_P1a | UniProt |  | A0A3B3WUA4 | N71 N90 |  |
|  | *Poecilia reticulata* | Bf_Pre_P1a | UniProt |  | A0A3P9PUE4 | N71 N90 | N233 |
|  | *Xiphophorus maculatus* | Bf_Xma_P1a | UniProt |  | M3ZQH5 | N71 | N233 |
|  | *Salarias fasciatus* | Bf_Sfa_P1a | UniProt |  | A0A672FTV6 | N71 | N233 |
|  | *Takifugu rubripes* | Bf_Tru_P1a | UniProt |  | H2SW28 | N71 |  |
|  | *Takifugu flavidus* | Bf_Tfl_P1a | UniProt |  | A0A5C6PGI8 | N71 |  |
|  | *Anabarilius grahami* | Bf_Agr_P1a | UniProt |  | A0A3N0YQI8 |  | N246 |
|  | *Mastacembelus armatus* | Bf_Mar_P1a | UniProt |  | A0A3Q3N3S7 | N71 |  |
|  | *Poecilia latipinna* | Bf_Pla_P1a | UniProt |  | A0A3B3UZ24 | N71 N90 | N233 |
|  | *Poecilia formosa* | Bf_Pfo_P1a | UniProt |  | A0A087Y1R8 | N71 N90 | N233 |
|  | *Cynoglossus semilaevis* | Bf_Cse_P1a | UniProt |  | A0A3P8V4K5 | N71 |  |
|  | *Tetraodon nigroviridis* | Bf_Tni_P1a | UniProt |  | H3CKJ7 | N71 | N233 |
|  | *Cyprinodon variegatus* | Bf_Cva_P1a | UniProt |  | A0A3Q2E008 | N71 | N228 |
|  | *Scleropages formosus* | Bf_Sfo_P1a | UniProt |  | A0A0N8K203 |  | N180 |
|  | *Danio rerio* | Bf_Dre_P1b | UniProt | Pannexin1b | F1QSR7 | N71 N95 | N246 |
|  | *Sinocyclocheilus anshuiensis* | Bf_San_P1b | UniProt |  | A0A671L8T2 | N71 | N246 |
|  | *Sinocyclocheilus rhinocerous* | Bf_Srh_P1b | UniProt |  | A0A673FW92 | N71 | N239 |
|  | *Danionella translucida* | Bf_Dtr_P1b | UniProt |  | A0A672PBI1 | N71 | N246 |
|  | *Triplophysa tibetana* | Bf_Tti_P1b | UniProt |  | A0A5A9P885 |  | N362 |
|  | *Pygocentrus nattereri* | Bf_Pna_P1b | UniProt |  | A0A3B4DT47 | N71 N90 | N245 |
|  | *Electrophorus electricus* | Bf_Eel_P1b | UniProt |  | A0A4W4EZT6 | N71 | N243 |
|  | *Astyanax mexicanus* | Bf_Ame_P1b | UniProt |  | A0A3B1IIW2 | N71 | N250 |
|  | *Pangasianodon hypophthalmus* | Bf_Phy_P1b | UniProt |  | A0A5N5JZA2 | N71 | N245 |
|  | *Ictalurus punctatus* | Bf_Ipu_P1b | UniProt |  | A0A2D0PTY9 | N72 | N237 |
|  | *Chanos chanos* | Bf_Cch_P1b | UniProt |  | A0A6J2W3L0 | N71 | N245 |
|  | *Bagarius yarrelli* | Bf_Bya_P1b | UniProt |  | A0A556UES4 | N91 | N265 |
|  | *Myripristis murdjan* | Bf_Mmu_P1b | UniProt |  | A0A667ZE16 | N71 | N233 |
|  | *Salmo salar* | Bf_Ssa_P1b | UniProt |  | A0A1S3RJJ6 | N71 | N248 |
|  | *Esox lucius* | Bf_Elu_P1b | UniProt |  | A0A3P8XS64 | N71 | N248 |
|  | *Hucho hucho* | Bf_Hhu_P1b | UniProt |  | A0A4W5JW91 | N71 | N248 |
|  | *Coregonus sp. 'balchen'* | Bf_Cba_P1b | UniProt |  | A0A6F9CVT5 | N71 | N248 |
|  | *Salmo trutta* | Bf_Str_P1b | UniProt |  | A0A673Z3D1 | N71 | N248 |
|  | *Seriola dumerili* | Bf_Sdu_P1b | UniProt |  | A0A3B4VD28 | N71 | N241 |
|  | *Seriola lalandi dorsalis* | Bf_Sdo_P1b | UniProt |  | A0A3B4X664 | N71 | N241 |
|  | *Perca flavescens* | Bf_Pfl_P1b | UniProt |  | A0A484DNZ5 | N71 | N241 |
|  | *Scophthalmus maximus* | Bf_Sma_P1b | UniProt |  | A0A2U9BQJ7 | N71 | N239 |
|  | *Gasterosteus aculeatus* | Bf_Gac_P1b | UniProt |  | G3Q632 | N71 | N242 |
|  | *Cottoperca gobio* | Bf_Cgo_P1b | UniProt |  | A0A6J2Q5M3 | N71 | N241 |
|  | *Lates calcarifer* | Bf_Lca_P1b | UniProt |  | A0A4W6BL78 | N71 | N241 |
|  | *Stegastes partitus* | Bf_Spa_P1b | UniProt |  | A0A3B5ALT6 |  | N244 |
|  | *Anabas testudineus* | Bf_Ate_P1b | UniProt |  | A0A3Q1JE93 | N70 | N242 |
|  | *Hippocampus comes* | Bf_Hco_P1b | UniProt |  | A0A3Q2YCM9 | N71 | N235 |
|  | *Echeneis naucrates* | Bf_Ena_P1b | UniProt |  | A0A665V2W5 | N71 | N241 |
|  | *Oreochromis aureus* | Bf_Oau_P1b | UniProt |  | A0A668TCG3 | N71 | N241 |
|  | *Oryzias latipes* | Bf_Ola_P1b | UniProt |  | H2MEM2 | N71 N93 | N241 |
|  | *Perca fluviatilis* | Bf_Pfu_P1b | UniProt |  | A0A6A5FS38 | N71 | N241 |
|  | *Larimichthys crocea* | Bf_Lcr_P1b | UniProt |  | A0A0F8CRU3 | N71 | N238 |
|  | *Acanthochromis polyacanthus* | Bf_Apo_P1b | UniProt |  | A0A3Q1FBA4 |  | N241 |
|  | *Neolamprologus brichardi* | Bf_Nbr_P1b | UniProt |  | A0A3Q4HZC0 | N71 | N241 |
|  | *Sphaeramia orbicularis* | Bf_Sor_P1b | UniProt |  | A0A673AHN3 | N71 | N242 |
|  | *Oreochromis niloticus* | Bf_Oni_P1b | UniProt |  | I3KRL4 | N71 | N241 |
|  | *Astatotilapia calliptera* | Bf_Aca_P1b | UniProt |  | A0A3P8Q4H6 | N71 | N241 |
|  | *Maylandia zebra* | Bf_Mze_P1b | UniProt |  | A0A3P9BTV9 | N71 | N241 |
|  | *Pundamilia nyererei* | Bf_Pny_P1b | UniProt |  | A0A3B4F980 | N71 | N241 |
|  | *Haplochromis burtoni* | Bf_Hbu_P1b | UniProt |  | A0A3Q3CYY2 | N71 | N241 |
|  | *Periophthalmus magnuspinnatus* | Bf_Pma_P1b | UniProt |  | A0A3B3ZW85 | N74 | N244 |
|  | *Oryzias melastigma* | Bf_Ome_P1b | UniProt |  | A0A3B3BSI9 | N71 N93 | N241 |
|  | *Amphilophus citrinellus* | Bf_Aci_P1b | UniProt |  | A0A3Q0SLZ4 | N71 | N248 |
|  | *Sparus aurata* | Bf_Sau_P1b | UniProt |  | A0A671WNS1 | N71 | N241 |
|  | *Amphiprion ocellaris* | Bf_Aoc_P1b | UniProt |  | A0A3Q1C039 | N71 | N241 |
|  | *Channa argus* | Bf_Car_P1b | UniProt |  | A0A6G1PAJ6 | N71 | N242 |
|  | *Austrofundulus limnaeus* | Bf_Ali_P1b | UniProt |  | A0A2I4C1Y6 | N71 | N241 |
|  | *Fundulus heteroclitus* | Bf_Fhe_P1b | UniProt |  | A0A3Q2TEU5 | N71 | N241 |
|  | *Amphiprion percula* | Bf_Ape_P1b | UniProt |  | A0A3P8TJG9 | N71 | N236 |
|  | *Kryptolebias marmoratus* | Bf_Kma_P1b | UniProt |  | A0A3Q3BR75 | N71 | N242 |
|  | *Oryzias javanicus* | Bf_Oja_P1b | UniProt |  | A0A437DLS5 | N71 N93 | N241 |
|  | *Labrus bergylta* | Bf_Lbe_P1b | UniProt |  | A0A3Q3GC43 | N71 | N227 |
|  | *Poecilia mexicana* | Bf_Pme_P1b | UniProt |  | A0A3B3XGG7 | N71 N93 | N241 |
|  | *Poecilia reticulata* | Bf_Pre_P1b | UniProt |  | A0A3P9MTP0 | N71 | N241 |
|  | *Xiphophorus maculatus* | Bf_Xma_P1b | UniProt |  | M3ZS53 | N71 | N241 |
|  | *Salarias fasciatus* | Bf_Sfa_P1b | UniProt |  | A0A672HSX5 | N71 N93 | N215 |
|  | *Takifugu rubripes* | Bf_Tru_P1b | UniProt |  | H2TPU6 | N71 | N241 |
|  | *Takifugu flavidus* | Bf_Tfl_P1b | UniProt |  | A0A5C6MU23 | N71 | N241 |
|  | *Anabarilius grahami* | Bf_Agr_P1b | UniProt |  | A0A3N0Y4M7 | N71 | N246 |
|  | *Mastacembelus armatus* | Bf_Mar_P1b | UniProt |  | A0A3Q3LER9 | N50 | N220 |
|  | *Poecilia latipinna* | Bf_Pla_P1b | UniProt |  | A0A3B3TUH4 | N60 N83 | N230 |
|  | *Poecilia formosa* | Bf_Pfo_P1b | UniProt |  | A0A087XZX3 | N60 N83 | N230 |
|  | *Cynoglossus semilaevis* | Bf_Cse_P1b | UniProt |  | A0A3P8VUF4 | N71 | N229 |
|  | *Tetraodon nigroviridis* | Bf_Tni_P1b | UniProt |  | H3CL66 | N71 | N244 |
|  | *Cyprinodon variegatus* | Bf_Cva_P1b | UniProt |  | A0A3Q2CTD3 | N71 | N241 |
|  | *Scleropages formosus* | Bf_Sfo_P1b | UniProt |  | A0A0P7UEI8 | N10 | N203 |
|  | *Danio rerio* | Bf_Dre_P2 | UniProt | Pannexin2 | B1P2E8 | N87 |  |
|  | *Sinocyclocheilus anshuiensis* | Bf_San_P2 | UniProt |  | A0A671KAW9 | N87 |  |
|  | *Sinocyclocheilus rhinocerous* | Bf_Srh_P2 | UniProt |  | A0A673KVR6 | N87 |  |
|  | *Danionella translucida* | Bf_Dtr_P2 | UniProt |  | A0A553Q5K6 | N87 |  |
|  | *Triplophysa tibetana* | Bf_Tti_P2 | UniProt |  | A0A5A9P1S8 | N87 |  |
|  | *Pygocentrus nattereri* | Bf_Pna_P2 | UniProt |  | A0A3B4CND9 | N87 |  |
|  | *Electrophorus electricus* | Bf_Eel_P2 | UniProt |  | A0A4W4FLN9 | N87 |  |
|  | *Astyanax mexicanus* | Bf_Ame_P2 | UniProt |  | W5LLB2 | N87 |  |
|  | *Pangasianodon hypophthalmus* | Bf_Phy_P2 | UniProt |  | A0A5N5P7K2 | N87 |  |
|  | *Ictalurus punctatus* | Bf_Ipu_P2 | UniProt |  | A0A2D0QTH6 | N87 |  |
|  | *Chanos chanos* | Bf_Cch_P2 | UniProt |  | A0A6J2WMP4 | N87 |  |
|  | *Bagarius yarrelli* | Bf_Bya_P2 | UniProt |  | A0A556TQY4 | N77 |  |
|  | *Myripristis murdjan* | Bf_Mmu_P2 | UniProt |  | A0A667XRP1 | N87 |  |
|  | *Salmo salar* | Bf_Ssa_P2 | UniProt |  | A0A1S3MKH2 | N87 |  |
|  | *Esox lucius* | Bf_Elu_P2 | UniProt |  | A0A3P8YYE5 | N87 |  |
|  | *Hucho hucho* | Bf_Hhu_P2 | UniProt |  | A0A4W5L5W5 | N87 |  |
|  | *Coregonus sp. 'balchen'* | Bf_Cba_P2 | UniProt |  | A0A6F8ZQS3 | N76 |  |
|  | *Salmo trutta* | Bf_Str_P2 | UniProt |  | A0A673XGZ7 | N87 |  |
|  | *Seriola dumerili* | Bf_Sdu_P2 | UniProt |  | A0A3B4VCG3 | N46 |  |
|  | *Seriola lalandi dorsalis* | Bf_Sdo_P2 | UniProt |  | A0A3B4XAI8 | N87 |  |
|  | *Perca flavescens* | Bf_Pfl_P2 | UniProt |  | A0A484D210 | N87 |  |
|  | *Scophthalmus maximus* | Bf_Sma_P2 | UniProt |  | A0A2U9BWT5 | N87 |  |
|  | *Gasterosteus aculeatus* | Bf_Gac_P2 | UniProt |  | G3PJL3 | N77 |  |
|  | *Cottoperca gobio* | Bf_Cgo_P2 | UniProt |  | A0A6J2PZ59 | N87 |  |
|  | *Lates calcarifer* | Bf_Lca_P2 | UniProt |  | A0A4W6EWW0 | N87 |  |
|  | *Stegastes partitus* | Bf_Spa_P2 | UniProt |  | A0A3B5A2L8 | N87 |  |
|  | *Anabas testudineus* | Bf_Ate_P2 | UniProt |  | A0A3Q1J7P4 | N87 |  |
|  | *Hippocampus comes* | Bf_Hco_P2 | UniProt |  | A0A3Q2XN59 | N87 |  |
|  | *Echeneis naucrates* | Bf_Ena_P2 | UniProt |  | A0A665V3A9 | N87 |  |
|  | *Oreochromis aureus* | Bf_Oau_P2 | UniProt |  | A0A668RTT2 | N87 |  |
|  | *Oryzias latipes* | Bf_Ola_P2 | UniProt |  | H2MNY7 | N87 |  |
|  | *Perca fluviatilis* | Bf_Pfu_P2 | UniProt |  | A0A6A5FC68 | N87 |  |
|  | *Larimichthys crocea* | Bf_Lcr_P2 | UniProt |  | A0A6G0IZ00 | N87 |  |
|  | *Acanthochromis polyacanthus* | Bf_Apo_P2 | UniProt |  | A0A3Q1ELQ8 | N87 |  |
|  | *Neolamprologus brichardi* | Bf_Nbr_P2 | UniProt |  | A0A3Q4HFT1 | N87 |  |
|  | *Sphaeramia orbicularis* | Bf_Sor_P2 | UniProt |  | A0A673BEE5 | N87 |  |
|  | *Oreochromis niloticus* | Bf_Oni_P2 | UniProt |  | I3JW76 | N87 |  |
|  | *Astatotilapia calliptera* | Bf_Aca_P2 | UniProt |  | A0A3P8NPF4 | N87 |  |
|  | *Maylandia zebra* | Bf_Mze_P2 | UniProt |  | A0A3P9CLK2 | N87 |  |
|  | *Pundamilia nyererei* | Bf_Pny_P2 | UniProt |  | A0A3B4H4H4 | N87 |  |
|  | *Haplochromis burtoni* | Bf_Hbu_P2 | UniProt |  | A0A3Q2W542 | N87 |  |
|  | *Periophthalmus magnuspinnatus* | Bf_Pma_P2 | UniProt |  | A0A3B4AR72 | N87 |  |
|  | *Oryzias melastigma* | Bf_Ome_P2 | UniProt |  | A0A3B3CUX5 | N87 |  |
|  | *Amphilophus citrinellus* | Bf_Aci_P2 | UniProt |  | A0A3Q0RSV8 | N87 |  |
|  | *Sparus aurata* | Bf_Sau_P2 | UniProt |  | A0A671THZ2 | N87 |  |
|  | *Amphiprion ocellaris* | Bf_Aoc_P2 | UniProt |  | A0A3Q1CYV0 | N87 |  |
|  | *Channa argus* | Bf_Car_P2 | UniProt |  | A0A6G1PFT8 | N87 |  |
|  | *Austrofundulus limnaeus* | Bf_Ali_P2 | UniProt |  | A0A2I4BWC2 | N87 |  |
|  | *Fundulus heteroclitus* | Bf_Fhe_P2 | UniProt |  | A0A3Q2PDM0 | N87 | N268 |
|  | *Amphiprion percula* | Bf_Ape_P2 | UniProt |  | A0A3P8SXB8 | N87 |  |
|  | *Kryptolebias marmoratus* | Bf_Kma_P2 | UniProt |  | A0A3Q3BIC9 | N87 |  |
|  | *Oryzias javanicus* | Bf_Oja_P2 | UniProt |  | A0A3S2MR15 | N87 |  |
|  | *Labrus bergylta* | Bf_Lbe_P2 | UniProt |  | A0A3Q3DZP2 | N87 |  |
|  | *Poecilia mexicana* | Bf_Pme_P2 | UniProt |  | A0A3B3WV28 | N87 |  |
|  | *Poecilia reticulata* | Bf_Pre_P2 | UniProt |  | A0A3P9QAM3 | N87 |  |
|  | *Xiphophorus maculatus* | Bf_Xma_P2 | UniProt |  | M4AXQ3 | N87 |  |
|  | *Salarias fasciatus* | Bf_Sfa_P2 | UniProt |  | A0A672GWY1 | N87 |  |
|  | *Takifugu rubripes* | Bf_Tru_P2 | UniProt |  | H2SGQ8 | N87 |  |
|  | *Takifugu flavidus* | Bf_Tfl_P2 | UniProt |  | A0A5C6P9Q8 | N87 |  |
|  | *Anabarilius grahami* | Bf_Agr_P2 | UniProt |  | A0A3N0YAX4 | N87 |  |
|  | *Mastacembelus armatus* | Bf_Mar_P2 | UniProt |  | A0A3Q3KP00 | N87 |  |
|  | *Poecilia latipinna* | Bf_Pla_P2 | UniProt |  | A0A3B3V2B8 | N87 |  |
|  | *Poecilia formosa* | Bf_Pfo_P2 | UniProt |  | A0A087XGV3 | N87 |  |
|  | *Cynoglossus semilaevis* | Bf_Cse_P2 | UniProt |  | A0A3P8VQX6 | N87 |  |
|  | *Tetraodon nigroviridis* | Bf_Tni_P2 | UniProt |  | H3CSG1 | N77 |  |
|  | *Cyprinodon variegatus* | Bf_Cva_P2 | UniProt |  | A0A3Q2D4H8 | N87 |  |
|  | *Scleropages formosus* | Bf_Sfo_P2 | UniProt |  | A0A0P7XI67 | N87 |  |
|  | *Danio rerio* | Bf_Dre_P3 | UniProt | Pannexin3 | E7F7V4 | N72 |  |
|  | *Sinocyclocheilus anshuiensis* | Bf_San_P3 | UniProt |  | A0A671L521 | N72 |  |
|  | *Sinocyclocheilus rhinocerous* | Bf_Srh_P3 | UniProt |  | A0A673MLT3 | N72 |  |
|  | *Danionella translucida* | Bf_Dtr_P3 | UniProt |  | A0A553QVV1 | N72 |  |
|  | *Triplophysa tibetana* | Bf_Tti_P3 | UniProt |  | A0A5A9N4S3 | N72 |  |
|  | *Pygocentrus nattereri* | Bf_Pna_P3 | UniProt |  | A0A3B4CPT8 | N72 |  |
|  | *Electrophorus electricus* | Bf_Eel_P3 | UniProt |  | A0A4W4FRU4 | N72 |  |
|  | *Astyanax mexicanus* | Bf_Ame_P3 | UniProt |  | W5KA10 | N71 |  |
|  | *Pangasianodon hypophthalmus* | Bf_Phy_P3 | UniProt |  | A0A5N5LNL5 | N107 |  |
|  | *Ictalurus punctatus* | Bf_Ipu_P3 | UniProt |  | A0A2D0SMD3 | N72 |  |
|  | *Chanos chanos* | Bf_Cch_P3 | UniProt |  | A0A6J2WWV2 | N72 |  |
|  | *Bagarius yarrelli* | Bf_Bya_P3 | UniProt |  | A0A556TJY5 | N72 |  |
|  | *Myripristis murdjan* | Bf_Mmu_P3 | UniProt |  | A0A667ZNQ4 | N72 |  |
|  | *Salmo salar* | Bf_Ssa_P3 | UniProt |  | A0A1S3RMN9 | N72 |  |
|  | *Esox lucius* | Bf_Elu_P3 | UniProt |  | A0A3P8YHY2 | N72 |  |
|  | *Hucho hucho* | Bf_Hhu_P3 | UniProt |  | A0A4W5NLP5 | N72 |  |
|  | *Coregonus sp. 'balchen'* | Bf_Cba_P3 | UniProt |  | A0A6F9A0G1 | N72 |  |
|  | *Salmo trutta* | Bf_Str_P3 | UniProt |  | A0A673WS30 | N71 |  |
|  | *Seriola dumerili* | Bf_Sdu_P3 | UniProt |  | A0A3B4VHK2 | N72 |  |
|  | *Seriola lalandi dorsalis* | Bf_Sdo_P3 | UniProt |  | A0A3B4WAR5 | N72 |  |
|  | *Perca flavescens* | Bf_Pfl_P3 | UniProt |  | A0A484CRH5 | N72 |  |
|  | *Scophthalmus maximus* | Bf_Sma_P3 | UniProt |  | A0A6A4T349 | N72 |  |
|  | *Gasterosteus aculeatus* | Bf_Gac_P3 | UniProt |  | G3Q9M3 | N72 |  |
|  | *Cottoperca gobio* | Bf_Cgo_P3 | UniProt |  | A0A6J2R349 | N72 |  |
|  | *Lates calcarifer* | Bf_Lca_P3 | UniProt |  | A0A4W6DTS7 | N72 |  |
|  | *Stegastes partitus* | Bf_Spa_P3 | UniProt |  | A0A3B4ZLA5 | N71 |  |
|  | *Anabas testudineus* | Bf_Ate_P3 | UniProt |  | A0A3Q1K2B4 | N71 |  |
|  | *Hippocampus comes* | Bf_Hco_P3 | UniProt |  | A0A3Q2XP86 | N71 |  |
|  | *Echeneis naucrates* | Bf_Ena_P3 | UniProt |  | A0A665TIL4 | N72 |  |
|  | *Oreochromis aureus* | Bf_Oau_P3 | UniProt |  | A0A668SED9 | N72 |  |
|  | *Oryzias latipes* | Bf_Ola_P3 | UniProt |  | H2MXX4 | N73 |  |
|  | *Perca fluviatilis* | Bf_Pfu_P3 | UniProt |  | A0A6A5EX38 | N72 |  |
|  | *Larimichthys crocea* | Bf_Lcr_P3 | UniProt |  | A0A6G0I9R0 | N72 |  |
|  | *Acanthochromis polyacanthus* | Bf_Apo_P3 | UniProt |  | A0A3Q1EDM7 | N71 |  |
|  | *Neolamprologus brichardi* | Bf_Nbr_P3 | UniProt |  | A0A3Q4GB35 | N71 |  |
|  | *Sphaeramia orbicularis* | Bf_Sor_P3 | UniProt |  | A0A673ABS3 | N72 |  |
|  | *Oreochromis niloticus* | Bf_Oni_P3 | UniProt |  | I3KFG2 | N72 |  |
|  | *Astatotilapia calliptera* | Bf_Aca_P3 | UniProt |  | A0A3P8NE92 | N72 |  |
|  | *Maylandia zebra* | Bf_Mze_P3 | UniProt |  | A0A3P9DAX0 | N72 |  |
|  | *Pundamilia nyererei* | Bf_Pny_P3 | UniProt |  | A0A3B4GDP5 | N71 |  |
|  | *Haplochromis burtoni* | Bf_Hbu_P3 | UniProt |  | A0A3Q2X5L3 | N72 |  |
|  | *Periophthalmus magnuspinnatus* | Bf_Pma_P3 | UniProt |  | A0A3B3ZUD6 | N71 |  |
|  | *Oryzias melastigma* | Bf_Ome_P3 | UniProt |  | A0A3B3BX68 | N72 |  |
|  | *Amphilophus citrinellus* | Bf_Aci_P3 | UniProt |  | A0A3Q0SGQ7 | N71 |  |
|  | *Sparus aurata* | Bf_Sau_P3 | UniProt |  | A0A671U344 | N72 |  |
|  | *Amphiprion ocellaris* | Bf_Aoc_P3 | UniProt |  | A0A3Q1BJJ4 | N71 |  |
|  | *Channa argus* | Bf_Car_P3 | UniProt |  | A0A6G1QW05 | N72 |  |
|  | *Austrofundulus limnaeus* | Bf_Ali_P3 | UniProt |  | A0A2I4D280 | N72 |  |
|  | *Fundulus heteroclitus* | Bf_Fhe_P3 | UniProt |  | A0A3Q2NV88 | N72 |  |
|  | *Amphiprion percula* | Bf_Ape_P3 | UniProt |  | A0A3P8T615 | N71 |  |
|  | *Kryptolebias marmoratus* | Bf_Kma_P3 | UniProt |  | A0A3Q3B131 | N72 |  |
|  | *Oryzias javanicus* | Bf_Oja_P3 | UniProt |  | A0A3S2P3R3 | N100 |  |
|  | *Labrus bergylta* | Bf_Lbe_P3 | UniProt |  | A0A3Q3E0L6 | N71 |  |
|  | *Poecilia mexicana* | Bf_Pme_P3 | UniProt |  | A0A3B3Z5S4 | N72 |  |
|  | *Poecilia reticulata* | Bf_Pre_P3 | UniProt |  | A0A3P9P9Y5 | N72 |  |
|  | *Xiphophorus maculatus* | Bf_Xma_P3 | UniProt |  | M3ZVF8 | N72 |  |
|  | *Salarias fasciatus* | Bf_Sfa_P3 | UniProt |  | A0A672JKA5 | N72 |  |
|  | *Takifugu rubripes* | Bf_Tru_P3 | UniProt |  | H2S112 | N72 |  |
|  | *Takifugu flavidus* | Bf_Tfl_P3 | UniProt |  | A0A5C6P3G4 | N72 |  |
|  | *Anabarilius grahami* | Bf_Agr_P3 | UniProt |  | A0A3N0Z7J9 | N72 |  |
|  | *Mastacembelus armatus* | Bf_Mar_P3 | UniProt |  | A0A3Q3KVF1 | N72 |  |
|  | *Poecilia latipinna* | Bf_Pla_P3 | UniProt |  | A0A3B3V7D9 | N72 |  |
|  | *Poecilia formosa* | Bf_Pfo_P3 | UniProt |  | A0A087YL18 | N72 |  |
|  | *Cynoglossus semilaevis* | Bf_Cse_P3 | UniProt |  | A0A3P8WQW5 | N72 |  |
|  | *Tetraodon nigroviridis* | Bf_Tni_P3 | UniProt |  | Q4SL86 | N72 |  |
|  | *Cyprinodon variegatus* | Bf_Cva_P3 | UniProt |  | A0A3Q2ECT5 | N72 |  |
|  | *Scleropages formosus* | Bf_Sfo_P3 | UniProt |  | A0A0P7URP1 | N77 |  |

| **Amphibians** | *Xenopus laevis* | Am_Xla_P1 | UniProt | Pannexin1 | A0A1L8HJE1 |  | N257 |
| --- | --- | --- | --- | --- | --- | --- | --- |
|  | *Xenopus tropicalis* | Am_Xtr_P1 | UniProt |  | B3DLA5 |  | N257 |
|  | *Geotrypetes seraphini* | Am_Gse_P1 | UniProt |  | A0A6P8R863 |  | N255 |
|  | *Microcaecilia unicolor* | Am_Mun_P1 | UniProt |  | A0A6P7XWZ3 |  | N255 |
|  | *Rhinatrema bivittatum* | Am_Rbi_P1 | NCBI |  | XP_029458198.1 |  | N252 |
|  | *Nanorana parkeri* | Am_Npa_P1 | NCBI |  | XP_018412323.1 |  | N254 |
|  | *Xenopus laevis* | Am_Xla_P2 | UniProt | Panexin2 | A0A1L8GU32 | N86 |  |
|  | *Xenopus tropicalis* | Am_Xtr_P2 | UniProt |  | A0A6I8PZG3 | N86 |  |
|  | *Geotrypetes seraphini* | Am_Gse_P2 | UniProt |  | A0A6P8S737 | N86 |  |
|  | *Microcaecilia unicolor* | Am_Mun_P2 | UniProt |  | A0A6P7Z0M9 | N22 |  |
|  | *Rhinatrema bivittatum* | Am_Rbi_P2 | NCBI |  | XP_029472886.1 | N22 |  |
|  | *Nanorana parkeri* | Am_Npa_P2 | NCBI |  | XP_018432232.1 | N86 N111 |  |
|  | *Xenopus laevis* | Am_Xla_P3 | UniProt | Pannexin3 | A0A1L8FFR9 | N70 |  |
|  | *Xenopus tropicalis* | Am_Xtr_P3 | UniProt |  | F7D9M9 | N70 |  |
|  | *Geotrypetes seraphini* | Am_Gse_P3 | UniProt |  | A0A6P8NR87 | N71 N101 |  |
|  | *Microcaecilia unicolor* | Am_Mun_P3 | UniProt |  | A0A6P7ZW08 | N71 |  |
|  | *Rhinatrema bivittatum* | Am_Rbi_P3 | NCBI |  | XP_029430059.1 | N75 |  |
|  | *Nanorana parkeri* | Am_Npa_P3 | NCBI |  | XP_018424096.1 | N75 |  |

| **Reptiles** | *Podarcis muralis* | Re_Pmu_P1 | UniProt | Pannexin1 | A0A670IMW4 |  | N252 |
| --- | --- | --- | --- | --- | --- | --- | --- |
|  | *Pogona vitticeps* | Re_Pvi_P1 | UniProt |  | A0A6J0SD02 |  | N255 |
|  | *Thamnophis sirtalis* | Re_Tsi_P1 | UniProt |  | A0A6I9YUF7 |  | N255 |
|  | *Paroedura picta* | Re_Ppi_P1 | UniProt |  | A0A402EYY8 |  | N255 |
|  | *Anolis carolinensis* | Re_Aca_P1 | UniProt |  | G1KPN4 |  | N257 |
|  | *Notechis scutatus* | Re_Nsc_P1 | UniProt |  | A0A6J1V4V6 |  | N191 |
|  | *Alligator sinensis* | Re_Asi_P1 | UniProt |  | A0A1U8D704 |  | N260 |
|  | *Pelodiscus sinensis* | Re_Psi_P1 | UniProt |  | K7FDB5 |  | N195 |
|  | *Platysternon megacephalum* | Re_Pme_P1 | UniProt |  | A0A4D9DWP4 |  | N255 |
|  | *Gopherus agassizii* | Re_Gag_P1 | UniProt |  | A0A452GG09 |  | N236 N255 |
|  | *Pantherophis guttatus* | Re_Pgu_P1 | UniProt |  | A0A6P9B7R7 |  | N254 |
|  | *Crotalus tigris* | Re_Cti_P1 | NCBI |  | XM_039349094.1 |  | N254 |
|  | *Gekko japonicus* | Re_Gja_P1 | NCBI |  | XM_015410293.1 |  | N255 |
|  | *Lacerta agilis* | Re_Lag_P1 | NCBI |  | XM_033146133.1 |  | N252 |
|  | *Protobothrops mucrosquamatus* | Re_Pmu_P1 | NCBI |  | XM_015827201.1 |  | N254 |
|  | *Pseudonaja textilis* | Re_Pte_P1 | NCBI |  | XM_026706935.1 |  | N254 |
|  | *Python bivittatus* | Re_Pbi_P1 | NCBI |  | XM_025171585.1 |  | N208 |
|  | *Zootoca vivipara* | Re_Zvi_P1 | NCBI |  | XM_035114643.1 |  | N252 |
|  | *Podarcis muralis* | Re_Pmu_P2 | UniProt | Pannexin2 | A0A670J859 | N22 N47 |  |
|  | *Pogona vitticeps* | Re_Pvi_P2 | UniProt |  | A0A6J0V6Q1 | N190 N215 |  |
|  | *Thamnophis sirtalis* | Re_Tsi_P2 | UniProt |  | A0A6I9XGX2 | N86 N111 |  |
|  | *Paroedura picta* | Re_Ppi_P2 | UniProt |  | A0A402EH32 | N22 N47 |  |
|  | *Anolis carolinensis* | Re_Aca_P2 | UniProt |  | G1KAJ7 | N87 N112 |  |
|  | *Notechis scutatus* | Re_Nsc_P2 | UniProt |  | A0A6J1U8G9 | N86 N111 |  |
|  | *Alligator sinensis* | Re_Asi_P2 | UniProt |  | A0A1U7R380 | N15 |  |
|  | *Pelodiscus sinensis* | Re_Psi_P2 | UniProt |  | K7G9N8 | N190 |  |
|  | *Platysternon megacephalum* | Re_Pme_P2 | UniProt |  | A0A4D9F0Q2 | N22 |  |
|  | *Gopherus agassizii* | Re_Gag_P2 | UniProt |  | A0A452H583 | N22 |  |
|  | *Pantherophis guttatus* | Re_Pgu_P2 | UniProt |  | A0A6P9C350 | N86 N111 |  |
|  | *Crotalus tigris* | Re_Cti_P2 | NCBI |  | XM_039337190.1 | N22 |  |
|  | *Gekko japonicus* | Re_Gja_P2 | NCBI |  | XM_015411813.1 | N86 N111 |  |
|  | *Lacerta agilis* | Re_Lag_P2 | NCBI |  | XM_033162913.1 | N86 N111 |  |
|  | *Protobothrops mucrosquamatus* | Re_Pmu_P2 | NCBI |  | XM_015823749.1 | N86 N111 |  |
|  | *Pseudonaja textilis* | Re_Pte_P2 | NCBI |  | XM_026714087.1 | N86 N111 |  |
|  | *Python bivittatus* | Re_Pbi_P2 | NCBI |  | XM_007427564.3 | N86 N111 |  |
|  | *Zootoca vivipara* | Re_Zvi_P2 | NCBI |  | XM_035128399.1 | N86 N111 |  |
|  | *Podarcis muralis* | Re_Pmu_P3 | UniProt | Pannexin3 | A0A670JSK3 | N71 |  |
|  | *Pogona vitticeps* | Re_Pvi_P3 | UniProt |  | A0A6J0V2S6 | N71 |  |
|  | *Thamnophis sirtalis* | Re_Tsi_P3 | UniProt |  | A0A6I9Y8X1 | N71 |  |
|  | *Paroedura picta* | Re_Ppi_P3 | UniProt |  | A0A402F0J7 | N104 |  |
|  | *Anolis carolinensis* | Re_Aca_P3 | UniProt |  | H9G3D5 | N87 N112 |  |
|  | *Notechis scutatus* | Re_Nsc_P3 | UniProt |  | A0A6J1U9P4 | N71 |  |
|  | *Alligator sinensis* | Re_Asi_P3 | UniProt |  | A0A1U7SMB7 | N71 |  |
|  | *Pelodiscus sinensis* | Re_Psi_P3 | UniProt |  | K7G7H3 | N71 N98 |  |
|  | *Platysternon megacephalum* | Re_Pme_P3 | UniProt |  | A0A4D9ECX9 | N71 N98 |  |
|  | *Gopherus agassizii* | Re_Gag_P3 | UniProt |  | A0A452GF72 | N71 N98 |  |
|  | *Pantherophis guttatus* | Re_Pgu_P3 | UniProt |  | A0A6P9C0R1 | N71 |  |
|  | *Crotalus tigris* | Re_Cti_P3 | NCBI |  | XM_039356601.1 | N71 |  |
|  | *Gekko japonicus* | Re_Gja_P3 | NCBI |  | XM_015429388.1 | N71 |  |
|  | *Lacerta agilis* | Re_Lag_P3 | NCBI |  | XM_033172461.1 | N70 |  |
|  | *Protobothrops mucrosquamatus* | Re_Pmu_P3 | NCBI |  | XM_015820106.1 | N71 |  |
|  | *Pseudonaja textilis* | Re_Pte_P3 | NCBI |  | XM_026718087.1 | N71 |  |
|  | *Python bivittatus* | Re_Pbi_P3 | NCBI |  | XM_007431037.3 | N71 |  |
|  | *Zootoca vivipara* | Re_Zvi_P3 | NCBI |  | XM_035139422.1 | N71 |  |

| **Birds** | *Taeniopygia guttata* | Bi_Tgu_P1 | UniProt | Pannexin1 | H0ZRG4 |  | N255 |
| --- | --- | --- | --- | --- | --- | --- | --- |
|  | *Hirundo rustica rustica* | Bi_Hru_P1 | UniProt |  | A0A3M0K2N6 |  | N255 |
|  | *Lonchura striata domestica* | Bi_Lst_P1 | UniProt |  | A0A218V546 |  | N255 |
|  | *Strigops habroptila* | Bi_Sha_P1 | UniProt |  | A0A672TNC5 |  | N255 |
|  | *Phasianus colchicus* | Bi_Pco_P1 | UniProt |  | A0A669P1W3 |  | N255 |
|  | *Aythya fuligula* | Bi_Afu_P1 | UniProt |  | A0A6J3EI41 |  | N255 |
|  | *Colinus virginianus* | Bi_Cvi_P1 | UniProt |  | A0A226PFQ7 |  | N255 |
|  | *Gallus gallus* | Bi_Gga_P1 | UniProt |  | A0A1D5P0I0 |  | N255 |
|  | *Patagioenas fasciata monilis* | Bi_Pfa_P1 | UniProt |  | A0A1V4L1E5 |  | N255 |
|  | *Meleagris gallopavo* | Bi_Mga_P1 | UniProt |  | G1NQM7 |  | N237 |
|  | *Callipepla squamata* | Bi_Csq_P1 | UniProt |  | A0A226MUM5 |  | N255 |
|  | *Ficedula albicollis* | Bi_Fal_P1 | UniProt |  | U3JC15 |  | N219 |
|  | *Geospiza fortis* | Bi_Gfo_P1 | UniProt |  | A0A6I9ZCS6 |  | N199 |
|  | *Charadrius vociferus* | Bi_Cvo_P1 | UniProt |  | A0A0A0AB22 |  | N195 |
|  | *Nipponia nippon* | Bi_Nni_P1 | UniProt |  | A0A091VK66 |  | N195 |
|  | *Calypte anna* | Bi_Can_P1 | UniProt |  | A0A091J2I8 |  | N195 |
|  | *Columba livia* | Bi_Cli_P1 | UniProt |  | A0A2I0LVR2 |  | N192 |
|  | *Gopherus agassizii* | Bi_Gag_P1 | UniProt |  | A0A452GG09 |  | N255 |
|  | *Struthio camelus australis* | Bi_Sca_P1 | UniProt |  | A0A093K6J7 |  | N195 |
|  | *Fulmarus glacialis* | Bi_Fgl_P1 | UniProt |  | A0A093IYR1 |  | N195 |
|  | *Merops nubicus* | Bi_Mnu_P1 | UniProt |  | A0A091S372 |  | N195 |
|  | *Acanthisitta chloris* | Bi_Ach_P1 | NCBI |  | XP_009076023.1 |  | N204 |
|  | *Anas platyrhynchos* | Bi_Apl_P1 | NCBI |  | XP_027323298.1 |  | N255 |
|  | *Anser cygnoides domesticus* | Bi_Acy_P1 | NCBI |  | XP_013033618.1 |  | N219 |
|  | *Antrostomus carolinensis* | Bi_Aca_P1 | NCBI |  | XP_010171917.1 |  | N194 |
|  | *Aptenodytes forsteri* | Bi_Afo_P1 | NCBI |  | XP_019328318.1 |  | N210 |
|  | *Apteryx rowi* | Bi_Aro_P1 | NCBI |  | XP_025927712.1 |  | N255 |
|  | *Aquila chrysaetos chrysaetos* | Bi_Acc_P1 | NCBI |  | XP_029898840.1 |  | N255 |
|  | *Calidris pugnax* | Bi_Cpu_P1 | NCBI |  | XP_014813746.1 |  | N225 |
|  | *Camarhynchus parvulus* | Bi_Cpa_P1 | NCBI |  | XP_030815036.1 |  | N255 |
|  | *Catharus ustulatus* | Bi_Cus_P1 | NCBI |  | XP_032908988.1 |  | N255 |
|  | *Chiroxiphia lanceolata* | Bi_Cla_P1 | NCBI |  | XP_032534515.1 |  | N254 |
|  | *Corapipo altera* | Bi_Cal_P1 | NCBI |  | XP_027499441.1 |  | N255 |
|  | *Corvus moneduloides* | Bi_Cmo_P1 | NCBI |  | XP_031955078.1 |  | N255 |
|  | *Coturnix japonica* | Bi_Cja_P1 | NCBI |  | XP_015707793.1 |  | N255 |
|  | *Cyanistes caeruleus* | Bi_Cca_P1 | NCBI |  | XP_023775741.1 |  | N215 |
|  | *Cygnus atratus* | Bi_Cat_P1 | NCBI |  | XP_035398581.1 |  | N255 |
|  | *Dromaius novaehollandiae* | Bi_Dno_P1 | NCBI |  | XP_025964969.1 |  | N255 |
|  | *Egretta garzetta* | Bi_Ega_P1 | NCBI |  | XP_009643220.1 |  | N224 |
|  | *Empidonax traillii* | Bi_Etr_P1 | NCBI |  | XP_027739108.1 |  | N255 |
|  | *Falco rusticolus* | Bi_Fru_P1 | NCBI |  | XP_037234097.1 |  | N255 |
|  | *Haliaeetus leucocephalus* | Bi_Hle_P1 | NCBI |  | XP_010576567.1 |  | N255 |
|  | *Manacus vitellinus* | Bi_Mvi_P1 | NCBI |  | XP_017927036.3 |  | N255 |
|  | *Melopsittacus undulatus* | Bi_Mun_p1 | NCBI |  | XP_012984683.2 |  | N255 |
|  | *Molothrus ater* | Bi_Mat_P1 | NCBI |  | XP_036246015.1 |  | N255 |
|  | *Motacilla alba alba* | Bi_Mal_P1 | NCBI |  | XP_038007064.1 |  | N255 |
|  | *Neopelma chrysocephalum* | Bi_Nch_p1 | NCBI |  | XP_027533209.1 |  | N255 |
|  | *Numida meleagris* | Bi_Nme_P1 | NCBI |  | XP_021237585.1 |  | N255 |
|  | *Oxyura jamaicensis* | Bi_Oja_P1 | NCBI |  | XP_035184553.1 |  | N255 |
|  | *Parus major* | Bi_Pma_P1 | NCBI |  | XP_015503561.1 |  | N255 |
|  | *Pipra filicauda* | Bi_Pfi_P1 | NCBI |  | XP_027591409.1 |  | N255 |
|  | *Pseudopodoces humilis* | Bi_Phu_P1 | NCBI |  | XP_005516796.1 |  | N261 |
|  | *Serinus canaria* | Bi_Scn_P1 | NCBI |  | XP_030090403.1 |  | N304 |
|  | *Sturnus vulgaris* | Bi_Svu_P1 | NCBI |  | XP_014733051.1 |  | N255 |
|  | *Zonotrichia albicollis* | Bi_Zal_P1 | NCBI |  | XP_005495791.1 |  | N255 |
|  | *Taeniopygia guttata* | Bi_Tgu_P2 | UniProt | Pannexin2 | H0Z0I1 | N86 |  |
|  | *Hirundo rustica rustica* | Bi_Hru_P2 | UniProt |  | A0A3M0KAL1 | N210 |  |
|  | *Lonchura striata domestica* | Bi_Lst_P2 | NCBI |  | XP_021410914.1 | N86 |  |
|  | *Strigops habroptila* | Bi_Sha_P2 | NCBI |  | XP_030335868.1 | N86 |  |
|  | *Phasianus colchicus* | Bi_Pco_P2 | UniProt |  | A0A669P1Z7 | N22 |  |
|  | *Aythya fuligula* | Bi_Afu_P2 | NCBI |  | XP_032046713.1 | N86 |  |
|  | *Colinus virginianus* | Bi_Cvi_P2 | UniProt |  | A0A226NUI9 | N22 |  |
|  | *Gallus gallus* | Bi_Gga_P2 | NCBI |  | XP_015128554.1 | N86 |  |
|  | *Patagioenas fasciata monilis* | Bi_Pfa_P2 | UniProt |  | A0A1V4K3B4 | N22 |  |
|  | *Meleagris gallopavo* | Bi_Mga_P2 | UniProt |  | H9H1C8 | N17 |  |
|  | *Callipepla squamata* | Bi_Csq_P2 | UniProt |  | A0A226N0J5 | N22 |  |
|  | *Ficedula albicollis* | Bi_Fal_P2 | UniProt |  | U3K4Y3 | N86 |  |
|  | *Geospiza fortis* | Bi_Gfo_P2 | NCBI |  | XP_030912808.1 | N22 |  |
|  | *Charadrius vociferus* | Bi_Cvo_P2 | UniProt |  | A0A0A0AGJ8 | N17 |  |
|  | *Nipponia nippon* | Bi_Nni_P2 | UniProt |  | A0A091UXU4 | N17 |  |
|  | *Calypte anna* | Bi_Can_P2 | UniProt |  | A0A091HW87 | N17 |  |
|  | *Columba livia* | Bi_Cli_P2 | UniProt |  | A0A2I0MH90 | N26 |  |
|  | *Gopherus agassizii* | Bi_Gag_P2 | UniProt |  | A0A452H583 | N22 |  |
|  | *Struthio camelus australis* | Bi_Sca_P2 | UniProt |  | A0A093HZT8 | N17 |  |
|  | *Fulmarus glacialis* | Bi_Fgl_P2 | UniProt |  | A0A093IRE2 | N17 |  |
|  | *Merops nubicus* | Bi_Mnu_P2 | UniProt |  | A0A091QTK3 | N17 |  |
|  | *Acanthisitta chloris* | Bi_Ach_P2 | NCBI |  | XP_009076173.1 | N10 |  |
|  | *Anas platyrhynchos* | Bi_Apl_P2 | NCBI |  | XP_027314162.1 | N86 |  |
|  | *Anser cygnoides domesticus* | Bi_Acy_P2 | NCBI |  | XP_013043831.1 | N25 |  |
|  | *Antrostomus carolinensis* | Bi_Aca_P2 | NCBI |  | XP_010175464.1 | N24 |  |
|  | *Aptenodytes forsteri* | Bi_Afo_P2 | NCBI |  | XP_009285132.1 | N22 |  |
|  | *Apteryx rowi* | Bi_Aro_P2 | NCBI |  | XP_025937285.1 | N86 |  |
|  | *Aquila chrysaetos chrysaetos* | Bi_Acc_P2 | NCBI |  | XP_029870402.1 | N86 |  |
|  | *Calidris pugnax* | Bi_Cpu_P2 | NCBI |  | XP_014819437.1 | N86 |  |
|  | *Camarhynchus parvulus* | Bi_Cpa_P2 | NCBI |  | XP_030816865.1 | N86 |  |
|  | *Catharus ustulatus* | Bi_Cus_P2 | NCBI |  | XP_032913314.1 | N86 |  |
|  | *Chiroxiphia lanceolata* | Bi_Cla_P2 | NCBI |  | XP_032544373.1 | N86 |  |
|  | *Corapipo altera* | Bi_Cal_P2 | NCBI |  | XP_027495494.1 | N86 |  |
|  | *Corvus moneduloides* | Bi_Cmo_P2 | NCBI |  | XP_031962041.1 | N86 |  |
|  | *Coturnix japonica* | Bi_Cja_P2 | NCBI |  | XP_032298541.1 | N86 |  |
|  | *Cyanistes caeruleus* | Bi_Cca_P2 | NCBI |  | XP_023774356.1 | N76 |  |
|  | *Cygnus atratus* | Bi_Cat_P2 | NCBI |  | XP_035415225.1 | N86 |  |
|  | *Dromaius novaehollandiae* | Bi_Dno_P2 | NCBI |  | XP_025968853.1 | N86 |  |
|  | *Egretta garzetta* | Bi_Ega_P2 | NCBI |  | XP_035749612.1 | N22 |  |
|  | *Empidonax traillii* | Bi_Etr_P2 | NCBI |  | XP_027750696.1 | N86 |  |
|  | *Falco rusticolus* | Bi_Fru_P2 | NCBI |  | XP_037245163.1 | N86 |  |
|  | *Haliaeetus leucocephalus* | Bi_Hle_P2 | NCBI |  | XP_010579620.1 | N126 |  |
|  | *Manacus vitellinus* | Bi_Mvi_P2 | NCBI |  | XP_029813918.1 | N71 |  |
|  | *Melopsittacus undulatus* | Bi_Mun_P2 | NCBI |  | XP_005146178.2 | N86 |  |
|  | *Molothrus ater* | Bi_Mat_P2 | NCBI |  | XP_036256424.1 | N86 |  |
|  | *Motacilla alba alba* | Bi_Mal_P2 | NCBI |  | XP_038010765.1 | N86 |  |
|  | *Neopelma chrysocephalum* | Bi_Nch_P2 | NCBI |  | XP_027535024.1 | N86 |  |
|  | *Numida meleagris* | Bi_Nme_P2 | NCBI |  | XP_021240408.1 | N86 |  |
|  | *Oxyura jamaicensis* | Bi_Oja_P2 | NCBI |  | XP_035193675.1 | N86 |  |
|  | *Parus major* | Bi_Pma_P2 | NCBI |  | XP_015484402.1 | N86 |  |
|  | *Pipra filicauda* | Bi_Pfi_P2 | NCBI |  | XP_027567659.1 | N86 |  |
|  | *Pseudopodoces humilis* | Bi_Phu_P2 | NCBI |  | XP_005519269.1 | N86 |  |
|  | *Serinus canaria* | Bi_Scn_P2 | NCBI |  | XP_009085052.2 | N86 |  |
|  | *Sturnus vulgaris* | Bi_Svu_P2 | NCBI |  | XP_014733257.1 | N86 |  |
|  | *Zonotrichia albicollis* | Bi_Zal_P2 | NCBI |  | XP_005481979.1 | N86 |  |
|  | *Taeniopygia guttata* | Bi_Tgu_P3 | UniProt | Pannexin3 | H0YR79 | N71 |  |
|  | *Hirundo rustica rustica* | Bi_Hru_P3 | UniProt |  | A0A3M0IZB0 | N61 |  |
|  | *Lonchura striata domestica* | Bi_Lst_P3 | UniProt |  | A0A218UUK2 | N71 |  |
|  | *Strigops habroptila* | Bi_Sha_P3 | UniProt |  | A0A672V6F5 | N103 | N273 |
|  | *Phasianus colchicus* | Bi_Pco_P3 | UniProt |  | A0A669Q018 | N71 |  |
|  | *Aythya fuligula* | Bi_Afu_P3 | UniProt |  | A0A6J3E864 | N71 |  |
|  | *Colinus virginianus* | Bi_Cvi_P3 | UniProt |  | A0A226PPB6 | N71 |  |
|  | *Gallus gallus* | Bi_Gga_P3 | UniProt |  | E1C4Y1 | N71 |  |
|  | *Patagioenas fasciata monilis* | Bi_Pfa_P3 | UniProt |  | A0A1V4J5Y1 | N71 |  |
|  | *Meleagris gallopavo* | Bi_Mga_P3 | UniProt |  | G1MQ73 | N71 |  |
|  | *Callipepla squamata* | Bi_Csq_P3 | UniProt |  | A0A226MLA8 | N71 |  |
|  | *Ficedula albicollis* | Bi_Fal_P3 | UniProt |  | U3JRK8 | N71 |  |
|  | *Geospiza fortis* | Bi_Gfo_P3 | UniProt |  | A0A6I9HUA3 | N71 |  |
|  | *Charadrius vociferus* | Bi_Cvo_P3 | UniProt |  | A0A0A0AZC0 | N71 |  |
|  | *Nipponia nippon* | Bi_Nni_P3 | UniProt |  | A0A091VF57 | N71 |  |
|  | *Calypte anna* | Bi_Can_P3 | UniProt |  | A0A091I7C6 | N71 |  |
|  | *Columba livia* | Bi_Cli_P3 | UniProt |  | A0A2I0LS85 | N23 |  |
|  | *Gopherus agassizii* | Bi_Gag_P3 | UniProt |  | A0A452GF72 | N71 N98 |  |
|  | *Struthio camelus australis* | Bi_Sca_P3 | UniProt |  | A0A093HZH4 | N79 |  |
|  | *Fulmarus glacialis* | Bi_Fgl_P3 | UniProt |  | A0A093IN65 | N71 |  |
|  | *Merops nubicus* | Bi_Mnu_P3 | UniProt |  | A0A091QRW3 | N52 |  |
|  | *Acanthisitta chloris* | Bi_Ach_P3 | NCBI |  | XP_009078415.1 | N75 |  |
|  | *Anas platyrhynchos* | Bi_Apl_P3 | NCBI |  | XP_005021150.2 | N112 |  |
|  | *Anser cygnoides domesticus* | Bi_Acy_P3 | NCBI |  | XP_013044092.1 | N199 |  |
|  | *Antrostomus carolinensis* | Bi_Aca_P3 | NCBI |  | XP_010165258.1 | N71 |  |
|  | *Aptenodytes forsteri* | Bi_Afo_P3 | NCBI |  | XP_009271296.1 | N59 |  |
|  | *Apteryx rowi* | Bi_Aro_P3 | NCBI |  | XP_025927543.1 | N71 |  |
|  | *Aquila chrysaetos chrysaetos* | Bi_Acc_P3 | NCBI |  | XP_029877108.1 | N71 |  |
|  | *Calidris pugnax* | Bi_Cpu_P3 | NCBI |  | XP_014816557.1 | N126 | N296 |
|  | *Camarhynchus parvulus* | Bi_Cpa_P3 | NCBI |  | XP_030820709.1 | N71 |  |
|  | *Catharus ustulatus* | Bi_Cus_P3 | NCBI |  | XP_032937775.1 | N71 |  |
|  | *Chiroxiphia lanceolata* | Bi_Cla_P3 | NCBI |  | XP_032565566.1 | N71 |  |
|  | *Corapipo altera* | Bi_Cal_P3 | NCBI |  | XP_027511312.1 | N71 |  |
|  | *Corvus moneduloides* | Bi_Cmo_P3 | NCBI |  | XP_031989432.1 | N71 | N241 |
|  | *Coturnix japonica* | Bi_Cja_P3 | NCBI |  | XP_015739660.1 | N71 |  |
|  | *Cyanistes caeruleus* | Bi_Cca_P3 | NCBI |  | XP_023797290.1 | N71 |  |
|  | *Cygnus atratus* | Bi_Cat_P3 | NCBI |  | XP_035411496.1 | N71 |  |
|  | *Dromaius novaehollandiae* | Bi_Dno_P3 | NCBI |  | XP_025978049.1 | N71 |  |
|  | *Egretta garzetta* | Bi_Ega_P3 | NCBI |  | XP_009640436.2 | N71 |  |
|  | *Empidonax traillii* | Bi_Etr_P3 | NCBI |  | XP_027753610.1 | N71 |  |
|  | *Falco rusticolus* | Bi_Fru_P3 | NCBI |  | XP_037265660.1 | N173 |  |
|  | *Haliaeetus leucocephalus* | Bi_Hle_P3 | NCBI |  | XP_010579651.1 | N71 |  |
|  | *Manacus vitellinus* | Bi_Mvi_P3 | NCBI |  | XP_008927671.2 | N71 |  |
|  | *Melopsittacus undulatus* | Bi_Mun_P3 | NCBI |  | XP_030899285.2 | N73 | N243 |
|  | *Molothrus ater* | Bi_Mat_P3 | NCBI |  | XP_036253401.1 | N71 |  |
|  | *Motacilla alba alba* | Bi_Mal_P3 | NCBI |  | XP_038017206.1 | N71 |  |
|  | *Neopelma chrysocephalum* | Bi_Nch_P3 | NCBI |  | XP_027555064.1 | N71 |  |
|  | *Numida meleagris* | Bi_Nme_P3 | NCBI |  | XP_021231805.1 | N71 |  |
|  | *Oxyura jamaicensis* | Bi_Oja_P3 | NCBI |  | XP_035201899.1 | N112 |  |
|  | *Parus major* | Bi_Pma_P3 | NCBI |  | XP_015505324.1 | N71 |  |
|  | *Pipra filicauda* | Bi_Pfi_P3 | NCBI |  | XP_027566534.1 | N71 |  |
|  | *Pseudopodoces humilis* | Bi_Phu_P3 | NCBI |  | XP_005528553.2 | N71 |  |
|  | *Serinus canaria* | Bi_Scn_P3 | NCBI |  | XP_009096107.2 | N71 |  |
|  | *Sturnus vulgaris* | Bi_Svu_P3 | NCBI |  | XP_014744271.1 | N71 |  |
|  | *Zonotrichia albicollis* | Bi_Zal_P3 | NCBI |  | XP_005494983.1 | N71 |  |

| **Mammals** | *Homo sapiens* | Ma_Hsa_P1 | UniProt | Pannexin1 | Q96RD7 |  | N255 |
| --- | --- | --- | --- | --- | --- | --- | --- |
|  | *Pan paniscus* | Ma_Ppa_P1 | UniProt |  | A0A2R9C7D5 |  | N255 |
|  | *Pan troglodytes* | Ma_Ptr_P1 | UniProt |  | H2Q4K2 |  | N255 |
|  | *Gorilla gorilla gorilla* | Ma_Ggo_P1 | UniProt |  | G3QS53 | N91 | N255 |
|  | *Pongo abelii* | Ma_Pab_P1 | UniProt |  | Q5REE3 |  | N255 |
|  | *Colobus angolensis palliatus* | Ma_Cpa_P1 | UniProt |  | A0A2K5JB28 |  | N255 |
|  | *Macaca mulatta* | Ma_Mma_P1 | UniProt |  | G7NBI3 |  | N255 |
|  | *Rhinopithecus bieti* | Ma_Rbi_P1 | UniProt |  | A0A2K6MUY4 |  | N255 |
|  | *Cercocebus atys* | Ma_Cat_P1 | UniProt |  | A0A2K5KIT1 |  | N255 |
|  | *Macaca nemestrina* | Ma_Mne_P1 | UniProt |  | A0A2K6CGA6 |  | N255 |
|  | *Macaca fascicularis* | Ma_Mfa_P1 | UniProt |  | A0A2K5TZS3 |  | N255 |
|  | *Papio anubi* | Ma_Pan_P1 | UniProt |  | A0A096MWA1 |  | N255 |
|  | *Rhinopithecus roxellana* | Ma_Rro_P1 | UniProt |  | A0A2K6PJL4 |  | N255 |
|  | *Chlorocebus sabaeus* | Ma_Csa_P1 | UniProt |  | A0A0D9S1A7 |  | N255 |
|  | *Nomascus leucogenys* | Ma_Nlu_P1 | UniProt |  | G1R636 |  | N254 |
|  | *Saimiri boliviensis* | Ma_Sbo_P1 | UniProt |  | A0A2K6S3C6 |  | N255 |
|  | *Callithrix jacchus* | Ma_Cja_P1 | UniProt |  | U3DZX6 |  | N255 |
|  | *Propithecus coquereli* | Ma_Pco_P1 | UniProt |  | A0A2K6EYV7 |  | N255 |
|  | *Cebus capucinus imitator* | Ma_Cim_P1 | UniProt |  | A0A2K5PBZ7 |  | N257 |
|  | *Sapajus apella* | Ma_Sap_P1 | UniProt |  | A0A6J3JBW1 |  | N257 |
|  | *Otolemur garnettii* | Ma_Oga_P1 | UniProt |  | H0XCZ4 |  | N255 |
|  | *Ictidomys tridecemlineatus* | Ma_Itr_P1 | UniProt |  | I3M8M2 |  | N255 |
|  | *Cavia porcellus* | Ma_Cpo_P1 | UniProt |  | A0A286XBS9 |  | N255 |
|  | *Heterocephalus glaber* | Ma_Hgl_P1 | UniProt |  | A0A0N8EUD1 |  | N255 |
|  | *Peromyscus maniculatus bairdii* | Ma_Pba_P1 | UniProt |  | A0A6I9MAQ6 |  | N254 |
|  | *Rattus norvegicus* | Ma_Rno_P1 | UniProt |  | P60570 |  | N254 |
|  | *Aotus nancymaae* | Ma_Ana_P1 | UniProt |  | A0A2K5DWN9 |  | N241 |
|  | *Cricetulus griseus* | Ma_Cgr_P1 | UniProt |  | G3HSP2 | N96 | N254 |
|  | *Mus musculus* | Ma_Mmu_P1 | UniProt |  | Q9JIP4 |  | N254 |
|  | *Equus caballus* | Ma_Eca_P1 | UniProt |  | F7C3W9 |  | N255 |
|  | *Mesocricetus auratus* | Ma_Mau_P1 | UniProt |  | A0A1U7QQT1 |  | N256 |
|  | *Trichechus manatus latirostris* | Ma_Tla_P1 | UniProt |  | A0A2Y9E395 |  | N255 |
|  | *Neotoma lepida* | Ma_Nle_P1 | UniProt |  | A0A1A6G167 |  | N271 |
|  | *Tursiops truncatus* | Ma_Ttr_P1 | UniProt |  | A0A2U3V8G6 |  | N255 |
|  | *Physeter macrocephalus* | Ma_Pma_P1 | UniProt |  | A0A2Y9FJ50 |  | N255 |
|  | *Neophocaena asiaeorientalis* | Ma_Nas_P1 | UniProt |  | A0A341A8Q8 |  | N255 |
|  | *Rhinolophus ferrumequinum* | Ma_Rfe_P1 | UniProt |  | A0A671FXP1 |  | N258 |
|  | *Lynx canadensis* | Ma_Lcn_P1 | UniProt |  | A0A667HL08 |  | N255 |
|  | *Felis catus* | Ma_Fca_P1 | UniProt |  | M3W287 |  | N255 |
|  | *Acinonyx jubatus* | Ma_Aju_P1 | UniProt |  | A0A6J0A8G9 |  | N255 |
|  | *Lipotes vexillifer* | Ma_Lve_P1 | UniProt |  | A0A340WPY6 |  | N255 |
|  | *Suricata suricatta* | Ma_Ssu_P1 | UniProt |  | A0A673TP15 |  | N254 |
|  | *Ursus arctos horribilis* | Ma_Uho_P1 | UniProt |  | A0A3Q7VJA0 |  | N254 |
|  | *Ursus americanus* | Ma_Uam_P1 | UniProt |  | A0A452QFW3 |  | N254 |
|  | *Enhydra lutris kenyoni* | Ma_Eke_P1 | UniProt |  | A0A2Y9KKV7 |  | N254 |
|  | *Canis lupus familiaris* | Ma_Cfa_P1 | UniProt |  | E2RSM3 |  | N254 |
|  | *Vulpes vulpes* | Ma_Vvu_P1 | UniProt |  | A0A3Q7RZJ5 |  | N254 |
|  | *Neomonachus schauinslandi* | Ma_Nsc_P1 | UniProt |  | A0A2Y9G6U7 |  | N255 |
|  | *Ailuropoda melanoleuca* | Ma_Ame_P1 | UniProt |  | G1LZM9 |  | N254 |
|  | *Sus scrofa* | Ma_Ssc_P1 | UniProt |  | A0A4X1TY57 |  | N255 |
|  | *Mustela putorius furo* | Ma_Mfu_P1 | UniProt |  | M3YLD5 |  | N254 |
|  | *Zalophus californianus* | Ma_Zca_P1 | UniProt |  | A0A6J2C0T6 |  | N255 |
|  | *Callorhinus ursinus* | Ma_Cur_P1 | UniProt |  | A0A3Q7QSN6 |  | N255 |
|  | *Odobenus rosmarus divergens* | Ma_Odi_P1 | UniProt |  | A0A2U3WNY3 |  | N252 |
|  | *Ovis aries* | Ma_Oar_P1 | UniProt |  | W5NUS9 |  | N255 |
|  | *Vombatus ursinus* | Ma_Vur_P1 | UniProt |  | A0A4X2KTV1 |  | N252 |
|  | *Capra hircus* | Ma_Chi_P1 | UniProt |  | A0A452E6G1 |  | N252 |
|  | *Bos taurus* | Ma_Bta_P1 | UniProt |  | D7R519 |  | N252 |
|  | *Phyllostomus discolor* | Ma_Pdi_P1 | UniProt |  | A0A6J2M191 |  | N255 |
|  | *Monodelphis domestica* | Ma_Mdo_P1 | UniProt |  | F6WB40 |  | N256 |
|  | *Bos mutus* | Ma_Bmu_P1 | UniProt |  | A0A6B0S0N1 |  | N252 |
|  | *Tupaia chinensis* | Ma_Tch_P1 | UniProt |  | L8YGG9 |  | N195 |
|  | *Lynx pardinus* | Ma_Lpa_P1 | UniProt |  | A0A485N7G6 |  | N251 |
|  | *Muntiacus muntjak* | Ma_Mmn_P1 | UniProt |  | A0A5N3WCF1 |  | N236 |
|  | *Erinaceus europaeus* | Ma_Eeu_P1 | UniProt |  | A0A1S2ZKI4 |  | N247 |
|  | *Ornithorhynchus anatinus* | Ma_Oan_P1 | UniProt |  | F6YGX2 |  | N255 |
|  | *Phascolarctos cinereus* | Ma_Pci_P1 | UniProt |  | A0A6P5LE79 |  | N255 |
|  | *Artibeus jamaicensis* | Ma_Aja_P1 | NCBI |  | XP_037009959.1 |  | N255 |
|  | *Arvicanthis niloticus* | Ma_Ani_P1 | NCBI |  | XP_034347766.1 |  | N254 |
|  | *Balaenoptera musculus* | Ma_Bms_P1 | NCBI |  | XP_036717529.1 |  | N255 |
|  | *Bubalus bubalis* | Ma_Bbu_P1 | NCBI |  | XP_025141944.1 |  | N252 |
|  | *Camelus ferus* | Ma_Cfe_P1 | NCBI |  | EPY73615.1 |  | N253 |
|  | *Ceratotherium simum simum* | Ma_Csi_P1 | NCBI |  | XP_004427496.1 |  | N255 |
|  | *Chinchilla lanigera* | Ma_Cla_P1 | NCBI |  | XP_005379739.1 |  | N255 |
|  | *Desmodus rotundus* | Ma_Dro_P1 | NCBI |  | XP_024430406.1 |  | N255 |
|  | *Elephantulus edwardii* | Ma_Eed_P1 | NCBI |  | XP_006902427.1 |  | N253 |
|  | *Eptesicus fuscus* | Ma_Efu_P1 | NCBI |  | XP_008147396.1 |  | N255 |
|  | *Fukomys damarensis* | Ma_Fda_P1 | NCBI |  | XP_010631970.1 |  | N255 |
|  | *Grammomys surdaster* | Ma_Gsu_P1 | NCBI |  | XP_028619221.1 |  | N254 |
|  | *Hipposideros armiger* | Ma_Har_P1 | NCBI |  | XP_019512717.1 |  | N257 |
|  | *Hyaena hyaena* | Ma_Hhy_P1 | NCBI |  | XP_039110936.1 |  | N255 |
|  | *Hylobates moloch* | Ma_Hmo_P1 | NCBI |  | XP_032023064.1 |  | N255 |
|  | *Jaculus jaculus* | Ma_Jja_P1 | NCBI |  | XP_004661987.1 |  | N254 |
|  | *Lontra canadensis* | Ma_Lca_P1 | NCBI |  | XP_032708683.1 |  | N255 |
|  | *Manis javanica* | Ma_Mja_P1 | NCBI |  | XP_036867488.1 |  | N254 |
|  | *Manis pentadactyla* | Ma_Mpe_P1 | NCBI |  | XP_036779199.1 |  | N255 |
|  | *Marmota flaviventris* | Ma_Mfl_P1 | NCBI |  | XP_027807195.1 |  | N255 |
|  | *Mastomys coucha* | Ma_Mco_P1 | NCBI |  | XP_031199851.1 |  | N255 |
|  | *Microtus ochrogaster* | Ma_Moc_P1 | NCBI |  | XP_005371751.1 |  | N254 |
|  | *Miniopterus natalensis* | Ma_Mna_P1 | NCBI |  | XP_016062181.1 |  | N245 |
|  | *Molossus molossus* | Ma_Mml_P1 | NCBI |  | XP_036115189.1 |  | N255 |
|  | *Monodon monoceros* | Ma_Mmo_P1 | NCBI |  | XP_029062770.1 |  | N255 |
|  | *Mus pahari* | Ma_Mpa_P1 | NCBI |  | XP_021062585.1 |  | N254 |
|  | *Mustela erminea* | Ma_Mer_P1 | NCBI |  | XP_032214138.1 |  | N254 |
|  | *Myotis myotis* | Ma_Mmy_P1 | NCBI |  | KAF6325381.1 |  | N255 |
|  | *Nannospalax galili* | Ma_Nga_P1 | NCBI |  | XP_008821339.2 |  | N254 |
|  | *Ochotona princeps* | Ma_Opr_P1 | NCBI |  | XP_004585229.1 |  | N258 |
|  | *Octodon degus* | Ma_Ode_P1 | NCBI |  | XP_004644824.1 |  | N255 |
|  | *Onychomys torridus* | Ma_Oto_P1 | NCBI |  | XP_036049172.1 |  | N254 |
|  | *Orcinus orca* | Ma_Oor_P1 | NCBI |  | XP_004265516.1 |  | N255 |
|  | *Panthera tigris altaica* | Ma_Pal_P1 | NCBI |  | XP_007079092.1 |  | N255 |
|  | *Piliocolobus tephrosceles* | Ma_Pte_P1 | NCBI |  | XP_023081403.1 |  | N255 |
|  | *Pipistrellus kuhlii* | Ma_Pku_P1 | NCBI |  | XP_036282607.1 |  | N255 |
|  | *Rattus rattus* | Ma_Rra_P1 | NCBI |  | XP_032765552.1 |  | N254 |
|  | *Rousettus aegyptiacus* | Ma_Rae_P1 | NCBI |  | XP_016014201.2 |  | N255 |
|  | *Sarcophilus harrisii* | Ma_Sha_P1 | NCBI |  | XP_003764363.1 |  | N255 |
|  | *Sorex araneus* | Ma_Sar_P1 | NCBI |  | XP_004605226.1 |  | N250 |
|  | *Trachypithecus francoisi* | Ma_Tfr_P1 | NCBI |  | XP_033063736.1 |  | N255 |
|  | *Trichosurus vulpecula* | Ma_Tvu_P1 | NCBI |  | XP_036600207.1 |  | N255 |
|  | *Urocitellus parryii* | Ma_Upa_P1 | NCBI |  | XP_026251872.1 |  | N255 |
|  | *Homo sapiens* | Ma_Hsa_P2 | UniProt | Pannexin2 | Q96RD6 | N86 |  |
|  | *Pan paniscus* | Ma_Ppa_P2 | UniProt |  | A0A2R9B2C5 | N10 |  |
|  | *Pan troglodytes* | Ma_Ptr_P2 | UniProt |  | H2RCN7 | N86 |  |
|  | *Gorilla gorilla gorilla* | Ma_Ggo_P2 | UniProt |  | G3REN9 | N10 |  |
|  | *Pongo abelii* | Ma_Pab_P2 | UniProt |  | A0A2J8XTC7 | N86 |  |
|  | *Colobus angolensis palliatus* | Ma_Cpa_P2 | UniProt |  | A0A2K5JE97 | N10 |  |
|  | *Macaca mulatta* | Ma_Mma_P2 | UniProt |  | F7GQW2 | N86 |  |
|  | *Rhinopithecus bieti* | Ma_Rbi_P2 | UniProt |  | A0A2K6K683 | N86 |  |
|  | *Cercocebus atys* | Ma_Cat_P2 | UniProt |  | A0A2K5NP46 | N86 |  |
|  | *Macaca nemestrina* | Ma_Mne_P2 | UniProt |  | A0A2K6AZ26 | N86 |  |
|  | *Macaca fascicularis* | Ma_Mfa_P2 | UniProt |  | A0A2K5WEJ8 | N86 |  |
|  | *Papio anubi* | Ma_Pan_P2 | UniProt |  | A0A096NKW4 | N86 |  |
|  | *Rhinopithecus roxellana* | Ma_Rro_P2 | UniProt |  | A0A2K6QFK2 | N86 |  |
|  | *Chlorocebus sabaeus* | Ma_Csa_P2 | UniProt |  | A0A0D9QYC0 | N76 |  |
|  | *Nomascus leucogenys* | Ma_Nlu_P2 | UniProt |  | G1QHZ5 | N10 |  |
|  | *Saimiri boliviensis* | Ma_Sbo_P2 | UniProt |  | A0A2K6SH06 | N68 |  |
|  | *Callithrix jacchus* | Ma_Cja_P2 | UniProt |  | U3ECX9 | N86 |  |
|  | *Propithecus coquereli* | Ma_Pco_P2 | UniProt |  | A0A2K6GDB8 | N79 |  |
|  | *Cebus capucinus imitator* | Ma_Cim_P2 | UniProt |  | A0A2K5QYT3 | N86 |  |
|  | *Sapajus apella* | Ma_Sap_P2 | UniProt |  | A0A6J3GUK9 | N86 |  |
|  | *Otolemur garnettii* | Ma_Oga_P2 | UniProt |  | H0XHR6 | N76 |  |
|  | *Ictidomys tridecemlineatus* | Ma_Itr_P2 | UniProt |  | I3NGV4 | N74 |  |
|  | *Cavia porcellus* | Ma_Cpo_P2 | UniProt |  | H0VYN7 | N73 |  |
|  | *Heterocephalus glaber* | Ma_Hgl_P2 | UniProt |  | G5B2I5 | N11 |  |
|  | *Peromyscus maniculatus bairdii* | Ma_Pba_P2 | UniProt |  | A0A6I9LF42 | N86 |  |
|  | *Rattus norvegicus* | Ma_Rno_P2 | UniProt |  | P60571 | N86 |  |
|  | *Aotus nancymaae* | Ma_Ana_P2 | UniProt |  | A0A2K5EAP5 | N86 |  |
|  | *Cricetulus griseus* | Ma_Cgr_P2 | UniProt |  | A0A3L7IAL3 | N76 |  |
|  | *Mus musculus* | Ma_Mmu_P2 | UniProt |  | Q6IMP4 | N86 |  |
|  | *Equus caballus* | Ma_Eca_P2 | UniProt |  | F6WMB5 | N86 |  |
|  | *Mesocricetus auratus* | Ma_Mau_P2 | UniProt |  | A0A1U7Q9L5 | N86 |  |
|  | *Trichechus manatus latirostris* | Ma_Tla_P2 | UniProt |  | A0A2Y9DRZ9 | N86 |  |
|  | *Neotoma lepida* | Ma_Nle_P2 | UniProt |  | A0A1A6H434 | N86 |  |
|  | *Tursiops truncatus* | Ma_Ttr_P2 | UniProt |  | A0A6J3S471 | N86 |  |
|  | *Physeter macrocephalus* | Ma_Pma_P2 | UniProt |  | A0A455BRV2 | N86 |  |
|  | *Neophocaena asiaeorientalis* | Ma_Nas_P2 | UniProt |  | A0A341ARA8 | N76 |  |
|  | *Rhinolophus ferrumequinum* | Ma_Rfe_P2 | UniProt |  | A0A671DWU1 | N86 |  |
|  | *Lynx canadensis* | Ma_Lcn_P2 | UniProt |  | A0A667GE52 | N86 |  |
|  | *Felis catus* | Ma_Fca_P2 | UniProt |  | M3WZJ0 | N86 |  |
|  | *Acinonyx jubatus* | Ma_Aju_P2 | UniProt |  | A0A6J2A8N2 | N86 |  |
|  | *Lipotes vexillifer* | Ma_Lve_P2 | UniProt |  | A0A340WX77 | N86 |  |
|  | *Suricata suricatta* | Ma_Ssu_P2 | UniProt |  | A0A673V4B6 | N40 |  |
|  | *Ursus arctos horribilis* | Ma_Uho_P2 | UniProt |  | A0A3Q7U433 | N53 |  |
|  | *Ursus americanus* | Ma_Uam_P2 | UniProt |  | A0A452RE23 | N76 |  |
|  | *Enhydra lutris kenyoni* | Ma_Eke_P2 | UniProt |  | A0A2Y9KUU6 | N86 |  |
|  | *Canis lupus familiaris* | Ma_Cfa_P2 | UniProt |  | F1Q3S2 | N31 |  |
|  | *Vulpes vulpes* | Ma_Vvu_P2 | UniProt |  | A0A3Q7V7D9 | N76 |  |
|  | *Neomonachus schauinslandi* | Ma_Nsc_P2 | UniProt |  | A0A2Y9GW88 | N135 |  |
|  | *Ailuropoda melanoleuca* | Ma_Ame_P2 | UniProt |  | G1L6D3 | N77 |  |
|  | *Sus scrofa* | Ma_Ssc_P2 | UniProt |  | F1RXR8 | N86 |  |
|  | *Mustela putorius furo* | Ma_Mfu_P2 | UniProt |  | M3XTJ0 | N86 |  |
|  | *Zalophus californianus* | Ma_Zca_P2 | UniProt |  | A0A6J2D7M5 | N86 |  |
|  | *Callorhinus ursinus* | Ma_Cur_P2 | UniProt |  | A0A3Q7PWV3 | N86 |  |
|  | *Odobenus rosmarus divergens* | Ma_Odi_P2 | UniProt |  | A0A2U3WLB4 | N86 |  |
|  | *Ovis aries* | Ma_Oar_P2 | UniProt |  | W5QEP8 | N86 |  |
|  | *Vombatus ursinus* | Ma_Vur_P2 | UniProt |  | A0A4X2JPB0 | N86 |  |
|  | *Capra hircus* | Ma_Chi_P2 | UniProt |  | A0A452EJ42 | N86 |  |
|  | *Bos taurus* | Ma_Bta_P2 | UniProt |  | G5E5X7 | N76 |  |
|  | *Phyllostomus discolor* | Ma_Pdi_P2 | UniProt |  | A0A6J2NGK3 | N86 |  |
|  | *Monodelphis domestica* | Ma_Mdo_P2 | UniProt |  | F7BXZ6 | N86 |  |
|  | *Bos mutus* | Ma_Bmu_P2 | UniProt |  | A0A6B0S1I4 | N86 |  |
|  | *Tupaia chinensis* | Ma_Tch_P2 | NCBI |  | XP_006157674.1 | N137 |  |
|  | *Lynx pardinus* | Ma_Lpa_P2 | UniProt |  | A0A485NY69 | N76 |  |
|  | *Muntiacus muntjak* | Ma_Mmn_P2 | UniProt |  | A0A5N3UQH5 | N86 |  |
|  | *Erinaceus europaeus* | Ma_Eeu_P2 | UniProt |  | A0A1S3W6F9 | N76 |  |
|  | *Ornithorhynchus anatinus* | Ma_Oan_P2 | UniProt |  | F6REB3 | N86 |  |
|  | *Phascolarctos cinereus* | Ma_Pci_P2 | UniProt |  | A0A6P5K055 | N86 |  |
|  | *Artibeus jamaicensis* | Ma_Aja_P2 | NCBI |  | XP_037016654.1 | N86 |  |
|  | *Arvicanthis niloticus* | Ma_Ani_P2 | NCBI |  | XP_034372977.1 | N86 |  |
|  | *Balaenoptera musculus* | Ma_Bms_P2 | NCBI |  | XP_036723320.1 | N86 |  |
|  | *Bubalus bubalis* | Ma_Bbu_P2 | NCBI |  | XP_006066378.1 | N86 |  |
|  | *Camelus ferus* | Ma_Cfe_P2 | NCBI |  | XP_032348456.1 | N86 |  |
|  | *Ceratotherium simum simum* | Ma_Csi_P2 | NCBI |  | XP_004438058.1 | N76 |  |
|  | *Chinchilla lanigera* | Ma_Cla_P2 | NCBI |  | XP_005379603.2 | N86 |  |
|  | *Desmodus rotundus* | Ma_Dro_P2 | NCBI |  | XP_024435428.1 | N77 |  |
|  | *Elephantulus edwardii* | Ma_Eed_P2 | NCBI |  | XP_006888084.1 | N86 |  |
|  | *Eptesicus fuscus* | Ma_Efu_P2 | NCBI |  | XP_027986859.1 | N86 |  |
|  | *Fukomys damarensis* | Ma_Fda_P2 | NCBI |  | XP_010632792.2 | N63 |  |
|  | *Grammomys surdaster* | Ma_Gsu_P2 | NCBI |  | XP_028609543.1 | N86 |  |
|  | *Hipposideros armiger* | Ma_Har_P2 | NCBI |  | XP_019524199.1 | N86 |  |
|  | *Hyaena hyaena* | Ma_Hhy_P2 | NCBI |  | XP_039090668.1 | N86 |  |
|  | *Hylobates moloch* | Ma_Hmo_P2 | NCBI |  | XP_031994640.1 | N86 |  |
|  | *Jaculus jaculus* | Ma_Jja_P2 | NCBI |  | XP_004650458.1 | N86 |  |
|  | *Lontra canadensis* | Ma_Lca_P2 | NCBI |  | XP_032738627.1 | N86 |  |
|  | *Manis javanica* | Ma_Mja_P2 | NCBI |  | XP_036862618.1 | N86 |  |
|  | *Manis pentadactyla* | Ma_Mpe_P2 | NCBI |  | XP_036785923.1 | N86 |  |
|  | *Marmota flaviventris* | Ma_Mfl_P2 | NCBI |  | XP_027801199.1 | N86 |  |
|  | *Mastomys coucha* | Ma_Mco_P2 | NCBI |  | XP_031208345.1 | N87 |  |
|  | *Microtus ochrogaster* | Ma_Moc_P2 | NCBI |  | XP_005354426.1 | N86 |  |
|  | *Miniopterus natalensis* | Ma_Mna_P2 | NCBI |  | XP_016067142.1 | N33 |  |
|  | *Molossus molossus* | Ma_Mml_P2 | NCBI |  | XP_036134165.1 | N86 |  |
|  | *Monodon monoceros* | Ma_Mmo_P2 | NCBI |  | XP_029061606.1 | N86 |  |
|  | *Mus pahari* | Ma_Mpa_P2 | NCBI |  | XP_021072578.1 | N86 |  |
|  | *Mustela erminea* | Ma_Mer_P2 | NCBI |  | XP_032201727.1 | N91 |  |
|  | *Myotis myotis* | Ma_Mmy_P2 | NCBI |  | XP_036167207.1 | N86 |  |
|  | *Nannospalax galili* | Ma_Nga_P2 | NCBI |  | XP_008832951.1 | N86 |  |
|  | *Ochotona princeps* | Ma_Opr_P2 | NCBI |  | XP_004589470.1 | N86 |  |
|  | *Octodon degus* | Ma_Ode_P2 | NCBI |  | XP_004642471.1 | N76 |  |
|  | *Onychomys torridus* | Ma_Oto_P2 | NCBI |  | XP_036064611.1 | N86 |  |
|  | *Orcinus orca* | Ma_Oor_P2 | NCBI |  | XP_004279653.1 | N86 |  |
|  | *Panthera tigris altaica* | Ma_Pal_P2 | NCBI |  | XP_007094099.1 | N37 |  |
|  | *Piliocolobus tephrosceles* | Ma_Pte_P2 | NCBI |  | XP_023078062.1 | N86 |  |
|  | *Pipistrellus kuhlii* | Ma_Pku_P2 | NCBI |  | XP_036285979.1 | N86 |  |
|  | *Rattus rattus* | Ma_Rra_P2 | NCBI |  | XP_032775238.1 | N86 |  |
|  | *Rousettus aegyptiacus* | Ma_Rae_P2 | NCBI |  | XP_015980844.2 | N86 |  |
|  | *Sarcophilus harrisii* | Ma_Sha_P2 | NCBI |  | XP_031794057.1 | N86 |  |
|  | *Sorex araneus* | Ma_Sar_P2 | NCBI |  | XP_004610637.1 | N86 |  |
|  | *Trachypithecus francoisi* | Ma_Tfr_P2 | NCBI |  | XP_033089402.1 | N86 |  |
|  | *Trichosurus vulpecula* | Ma_Tvu_P2 | NCBI |  | XP_036617560.1 | N86 |  |
|  | *Urocitellus parryii* | Ma_Upa_P2 | NCBI |  | XP_026269320.1 | N86 |  |
|  | *Homo sapiens* | Ma_Hsa_P3 | UniProt | Pannexin3 | Q96QZ0 | N71 |  |
|  | *Pan paniscus* | Ma_Ppa_P3 | UniProt |  | A0A2R9A6D7 | N71 |  |
|  | *Pan troglodytes* | Ma_Ptr_P3 | UniProt |  | H2Q511 | N71 |  |
|  | *Gorilla gorilla gorilla* | Ma_Ggo_P3 | UniProt |  | G3QMD9 | N71 |  |
|  | *Pongo abelii* | Ma_Pab_P3 | UniProt |  | H2NFQ4 | N71 |  |
|  | *Colobus angolensis palliatus* | Ma_Cpa_P3 | UniProt |  | A0A2K5JLU5 | N71 |  |
|  | *Macaca mulatta* | Ma_Mma_P3 | UniProt |  | F6THN1 | N71 |  |
|  | *Rhinopithecus bieti* | Ma_Rbi_P3 | UniProt |  | A0A2K6MV84 | N71 |  |
|  | *Cercocebus atys* | Ma_Cat_P3 | UniProt |  | A0A2K5MVP2 | N71 |  |
|  | *Macaca nemestrina* | Ma_Mne_P3 | UniProt |  | A0A2K6BEN2 | N71 |  |
|  | *Macaca fascicularis* | Ma_Mfa_P3 | UniProt |  | G7PPK7 | N71 |  |
|  | *Papio anubi* | Ma_Pan_P3 | UniProt |  | A0A096N4L5 | N71 |  |
|  | *Rhinopithecus roxellana* | Ma_Rro_P3 | UniProt |  | A0A2K6PEF4 | N71 |  |
|  | *Chlorocebus sabaeus* | Ma_Csa_P3 | UniProt |  | A0A0D9S467 | N71 |  |
|  | *Nomascus leucogenys* | Ma_Nlu_P3 | UniProt |  | G1R777 | N71 |  |
|  | *Saimiri boliviensis* | Ma_Sbo_P3 | UniProt |  | A0A2K6SDN2 | N71 |  |
|  | *Callithrix jacchus* | Ma_Cja_P3 | UniProt |  | F7I231 | N61 |  |
|  | *Propithecus coquereli* | Ma_Pco_P3 | UniProt |  | A0A2K6FAF4 | N71 |  |
|  | *Cebus capucinus imitator* | Ma_Cim_P3 | UniProt |  | A0A2K5PM93 | N71 |  |
|  | *Sapajus apella* | Ma_Sap_P3 | UniProt |  | A0A6J3IFP7 | N71 |  |
|  | *Otolemur garnettii* | Ma_Oga_P3 | UniProt |  | H0WXJ5 | N71 |  |
|  | *Ictidomys tridecemlineatus* | Ma_Itr_P3 | UniProt |  | I3MFF1 | N71 |  |
|  | *Cavia porcellus* | Ma_Cpo_P3 | UniProt |  | A0A286XWY5 | N71 |  |
|  | *Heterocephalus glaber* | Ma_Hgl_P3 | UniProt |  | G5C494 | N71 | N241 |
|  | *Peromyscus maniculatus bairdii* | Ma_Pba_P3 | UniProt |  | A0A6I9MNZ6 | N71 |  |
|  | *Rattus norvegicus* | Ma_Rno_P3 | UniProt |  | P60572 | N71 |  |
|  | *Aotus nancymaae* | Ma_Ana_P3 | UniProt |  | A0A2K5ES49 | N71 |  |
|  | *Cricetulus griseus* | Ma_Cgr_P3 | UniProt |  | G3IIB4 | N71 |  |
|  | *Mus musculus* | Ma_Mmu_P3 | UniProt |  | Q8CEG0 | N71 |  |
|  | *Equus caballus* | Ma_Eca_P3 | UniProt |  | F6TM26 | N71 |  |
|  | *Mesocricetus auratus* | Ma_Mau_P3 | UniProt |  | A0A1U7Q549 | N71 |  |
|  | *Trichechus manatus latirostris* | Ma_Tla_P3 | UniProt |  | A0A2Y9DSF5 | N71 |  |
|  | *Neotoma lepida* | Ma_Nle_P3 | UniProt |  | A0A1A6FUI4 | N71 |  |
|  | *Tursiops truncatus* | Ma_Ttr_P3 | UniProt |  | A0A2U4BWR8 | N71 |  |
|  | *Physeter macrocephalus* | Ma_Pma_P3 | UniProt |  | A0A2Y9F9T2 | N71 |  |
|  | *Neophocaena asiaeorientalis* | Ma_Nas_P3 | UniProt |  | A0A341B1D4 | N71 |  |
|  | *Rhinolophus ferrumequinum* | Ma_Rfe_P3 | UniProt |  | A0A671EDW3 | N71 |  |
|  | *Lynx canadensis* | Ma_Lcn_P3 | UniProt |  | A0A667GUA2 | N71 |  |
|  | *Felis catus* | Ma_Fca_P3 | UniProt |  | M3VYU2 | N71 |  |
|  | *Acinonyx jubatus* | Ma_Aju_P3 | UniProt |  | A0A6I9ZRI5 | N71 |  |
|  | *Lipotes vexillifer* | Ma_Lve_P3 | UniProt |  | A0A340XQL2 | N71 |  |
|  | *Suricata suricatta* | Ma_Ssu_P3 | UniProt |  | A0A673UKR9 | N71 |  |
|  | *Ursus arctos horribilis* | Ma_Uho_P3 | UniProt |  | A0A3Q7XES1 | N71 |  |
|  | *Ursus americanus* | Ma_Uam_P3 | UniProt |  | A0A452RQX3 | N71 |  |
|  | *Enhydra lutris kenyoni* | Ma_Eke_P3 | UniProt |  | A0A2Y9K7L0 | N71 |  |
|  | *Canis lupus familiaris* | Ma_Cfa_P3 | UniProt |  | F6X9T9 | N71 |  |
|  | *Vulpes vulpes* | Ma_Vvu_P3 | UniProt |  | A0A3Q7T450 | N71 |  |
|  | *Neomonachus schauinslandi* | Ma_Nsc_P3 | UniProt |  | A0A2Y9HKM4 | N71 |  |
|  | *Ailuropoda melanoleuca* | Ma_Ame_P3 | UniProt |  | D2HNJ7 | N71 |  |
|  | *Sus scrofa* | Ma_Ssc_P3 | UniProt |  | F1S7B4 | N71 |  |
|  | *Mustela putorius furo* | Ma_Mfu_P3 | UniProt |  | M3Y3U5 | N71 |  |
|  | *Zalophus californianus* | Ma_Zca_P3 | UniProt |  | A0A6J2BNI3 | N71 |  |
|  | *Callorhinus ursinus* | Ma_Cur_P3 | UniProt |  | A0A3Q7QV52 | N71 |  |
|  | *Odobenus rosmarus divergens* | Ma_Odi_P3 | UniProt |  | A0A2U3WW42 | N71 |  |
|  | *Ovis aries* | Ma_Oar_P3 | UniProt |  | W5PL29 | N71 |  |
|  | *Vombatus ursinus* | Ma_Vur_P3 | UniProt |  | A0A4X2M6S5 | N71 |  |
|  | *Capra hircus* | Ma_Chi_P3 | UniProt |  | A0A452E5V2 | N71 |  |
|  | *Bos taurus* | Ma_Bta_P3 | UniProt |  | E1BF03 | N71 |  |
|  | *Phyllostomus discolor* | Ma_Pdi_P3 | UniProt |  | A0A6J2MY87 | N71 | N251 |
|  | *Monodelphis domestica* | Ma_Mdo_P3 | UniProt |  | A0A5F8GYX9 | N71 |  |
|  | *Bos mutus* | Ma_Bmu_P3 | UniProt |  | L8I524 | N71 |  |
|  | *Tupaia chinensis* | Ma_Tch_P3 | UniProt |  | L9LC01 | N71 |  |
|  | *Lynx pardinus* | Ma_Lpa_P3 | UniProt |  | A0A485NRD7 | N71 |  |
|  | *Muntiacus muntjak* | Ma_Mmn_P3 | UniProt |  | A0A5N3W7D5 | N71 |  |
|  | *Erinaceus europaeus* | Ma_Eeu_P3 | UniProt |  | A0A1S2ZKE6 | N71 |  |
|  | *Ornithorhynchus anatinus* | Ma_Oan_P3 | UniProt |  | F7FBT0 | N71 |  |
|  | *Phascolarctos cinereus* | Ma_Pci_P3 | UniProt |  | A0A6P5LE82 | N71 |  |
|  | *Artibeus jamaicensis* | Ma_Aja_P3 | NCBI |  | XP_036990154.1 | N71 | N251 |
|  | *Arvicanthis niloticus* | Ma_Ani_P3 | NCBI |  | XP_034347210.1 | N71 |  |
|  | *Balaenoptera musculus* | Ma_Bms_P3 | NCBI |  | XP_036717877.1 | N71 |  |
|  | *Bubalus bubalis* | Ma_Bbu_P3 | NCBI |  | XP_006057699.1 | N71 |  |
|  | *Camelus ferus* | Ma_Cfe_P3 | NCBI |  | EPY88774.1 | N61 |  |
|  | *Ceratotherium simum simum* | Ma_Csi_P3 | NCBI |  | XP_004438471.1 | N71 |  |
|  | *Chinchilla lanigera* | Ma_Cla_P3 | NCBI |  | XP_005378486.1 | N71 |  |
|  | *Desmodus rotundus* | Ma_Dro_P3 | NCBI |  | XP_024412909.1 | N71 |  |
|  | *Elephantulus edwardii* | Ma_Eed_P3 | NCBI |  | XP_006892860.1 | N71 |  |
|  | *Eptesicus fuscus* | Ma_Efu_P3 | NCBI |  | XP_008140807.1 | N71 |  |
|  | *Fukomys damarensis* | Ma_Fda_P3 | NCBI |  | XP_010627024.1 | N71 |  |
|  | *Grammomys surdaster* | Ma_Gsu_P3 | NCBI |  | XP_028611748.1 | N71 |  |
|  | *Hipposideros armiger* | Ma_Har_P3 | NCBI |  | XP_019514417.1 | N71 |  |
|  | *Hyaena hyaena* | Ma_Hhy_P3 | NCBI |  | XP_039106088.1 | N71 |  |
|  | *Hylobates moloch* | Ma_Hmo_P3 | NCBI |  | XP_032023672.1 | N71 |  |
|  | *Jaculus jaculus* | Ma_Jja_P3 | NCBI |  | XP_004670660.1 | N71 |  |
|  | *Lontra canadensis* | Ma_Lca_P3 | NCBI |  | XP_032724944.1 | N71 |  |
|  | *Manis javanica* | Ma_Mja_P3 | NCBI |  | XP_036856700.1 | N71 |  |
|  | *Manis pentadactyla* | Ma_Mpe_P3 | NCBI |  | XP_036762610.1 | N71 |  |
|  | *Marmota flaviventris* | Ma_Mfl_P3 | NCBI |  | XP_027808948.1 | N70 |  |
|  | *Mastomys coucha* | Ma_Mco_P3 | NCBI |  | XP_031200342.1 | N71 |  |
|  | *Microtus ochrogaster* | Ma_Moc_P3 | NCBI |  | XP_005347114.1 | N71 | N241 |
|  | *Miniopterus natalensis* | Ma_Mna_P3 | NCBI |  | XP_016059870.1 | N71 |  |
|  | *Molossus molossus* | Ma_Mml_P3 | NCBI |  | XP_036133125.1 | N71 |  |
|  | *Monodon monoceros* | Ma_Mmo_P3 | NCBI |  | XP_029065382.1 | N71 |  |
|  | *Mus pahari* | Ma_Mpa_P3 | NCBI |  | XP_021063549.1 | N71 |  |
|  | *Mustela erminea* | Ma_Mer_P3 | NCBI |  | XP_032215910.1 | N71 |  |
|  | *Myotis myotis* | Ma_Mmy_P3 | NCBI |  | XP_036209098.1 | N71 | N252 |
|  | *Nannospalax galili* | Ma_Nga_P3 | NCBI |  | XP_008833384.1 | N71 |  |
|  | *Ochotona princeps* | Ma_Opr_P3 | NCBI |  | XP_004597767.1 | N71 |  |
|  | *Octodon degus* | Ma_Ode_P3 | NCBI |  | XP_004641432.1 | N71 |  |
|  | *Onychomys torridus* | Ma_Oto_P3 | NCBI |  | XP_036047678.1 | N71 |  |
|  | *Orcinus orca* | Ma_Oor_P3 | NCBI |  | XP_004280746.1 | N70 |  |
|  | *Panthera tigris altaica* | Ma_Pal_P3 | NCBI |  | XP_007074112.1 | N71 |  |
|  | *Piliocolobus tephrosceles* | Ma_Pte_P3 | NCBI |  | XP_023064293.1 | N71 |  |
|  | *Pipistrellus kuhlii* | Ma_Pku_P3 | NCBI |  | XP_036304963.1 | N71 |  |
|  | *Rattus rattus* | Ma_Rra_P3 | NCBI |  | XP_032766411.1 | N71 |  |
|  | *Rousettus aegyptiacus* | Ma_Rae_P3 | NCBI |  | XP_015980403.2 | N71 |  |
|  | *Sarcophilus harrisii* | Ma_Sha_P3 | NCBI |  | XP_003764390.1 | N71 |  |
|  | *Sorex araneus* | Ma_Sar_P3 | NCBI |  | XP_004604838.1 | N71 |  |
|  | *Trachypithecus francoisi* | Ma_Tfr_P3 | NCBI |  | XP_033063462.1 | N71 |  |
|  | *Trichosurus vulpecula* | Ma_Tvu_P3 | NCBI |  | XP_036598858.1 | N71 |  |
|  | *Urocitellus parryii* | Ma_Upa_P3 | NCBI |  | XP_026260355.1 | N71 |  |
